# Supplementary material for: Targeting SMYD2 improves immunotherapy response in experimental hepatocellular carcinoma
Source: Mol Ther Oncol. 2026 May 20;34(2):201239. doi: 10.1016/j.omton.2026.201239 (PMC13266103; doi:10.1016/j.omton.2026.201239)
Supplement: Document S1. Figures S1–S13 and Table S1 [file mmc1.pdf]

OMTON, Volume *34*

Supplemental information

Targeting SMYD2 improves immunotherapy response in experimental hepatocellular carcinoma

Bárbara Bueloni, Mariel Fusco, Esteban Fiore, Mariana Malvicini, María Jose Cantero, Lucía Lameroli, Mailín Casadei, Brian Martinez Ruiz, Florencia Mercogliano, Eva Santamaría, Catalina Atorrasagasti, Josepmaria Argemi, Ali Canbay, Juan Bayo, and Guillermo Mazzolini

Supplemental materials and methods.

Bioinformatic analyses

For the initial assessment of SMYD2 expression, RSEM-normalized RNA-seq data from 15 independent HCC datasets were obtained from HCCDB v2, including their adjacent non-tumoral tissues. Additional adjacent non-tumoral tissues from The Cancer Genome Atlas (TCGA) were retrieved via Firebrowse (http://firebrowse.org), excluding tissue types with fewer than three samples to ensure consistency. Subsequent analyses focused on TCGA (n = 377, RNA-seq), the International Cancer Genome Consortium (ICGC, n = 203, RNA-seq), and GSE14520 (n = 225, microarray), as these represent well-characterized and widely used HCC transcriptomic datasets. These cohorts comprise patients with primary tumors who underwent surgical resection. Analyses were restricted to patients with a single tumor sample, considering only protein-coding genes expressed in at least one patient. For tumor versus non-tumoral comparisons, a subset of paired samples was selected (TCGA, n = 50; ICGC, n = 174; GSE14520, n = 214).

Gene expression values from RNA-seq datasets were log-transformed as log2(RSEM + 1). Differentially expressed genes (DEGs) were identified using paired t-tests with a false discovery rate (FDR) threshold of < 0.05. Gene ontology (GO) and pathway analyses were done using ToppGene (ToppFun) on DEGs with log2 fold change > |0.378| and FDR < 0.05. Upstream regulator analysis was carried out with Ingenuity Pathway Analysis (IPA®, Qiagen), on DEGs from tumor RNA-seq data (log2 fold change > |0.585|, FDR < 0.05), using the IPA z-score algorithm to predict regulator activation or inhibition based on curated molecular interactions. Immunophenoscores (IPS) for TCGA-LIHC samples were obtained from The Cancer Immunome Atlas (https://tcia.at) and integrated with SMYD2 expression data by matching sample identifiers. Samples were stratified into

SMYD2-high and SMYD2-low groups using the median SMYD2 expression value as cutoff. IPS values corresponding to different therapeutic scenarios ($CTLA4^{-}/PD1^{-}$, $CTLA4^{-}/PD1^{+}$, $CTLA4^{+}/PD1^{-}$, $CTLA4^{+}/PD1^{+}$) were analyzed. All analyses were performed in R, and graphs were generated using the *ggplot2* R package or GraphPad Prism.

In vivo HCC murine models

Male C3H/HeJ and female C57BL/6J mice (6–8 weeks old) were bred and housed in the Animal Resources Facilities at Austral University. All procedures were approved by the university's Animal Studies Committee and conducted in compliance with institutional and national regulations for animal research and welfare. Mice were monitored every other day for body weight and general health throughout all *in vivo* experiments. Tumor dissection, size measurement and weighing were performed in a blinded manner to minimize potential bias. For monotherapy experiments, animals received AZ-505 (40 mg/kg, MedKoo Biosciences), LLY-507 (10 mg/kg, MedKoo Biosciences) or vehicle (12.5% Cremophor EL, 12.5% DMSO in aqueous solution) via intraperitoneal (i.p.) injection three times per week for two weeks. In combination studies, anti-PD-1 antibody (Bio X Cell) was co-administered at 100 μg/mouse (100 μL of a 1 mg/mL solution in PBS) via i.p. injection, together with the first and fourth AZ-505 doses.

In the Hepa129 model, liver fibrosis was induced in C3H/HeJ mice by i.p. injection of thioacetamide (TAA, 200 mg/kg, Sigma-Aldrich) three times per week for four weeks. On day 30, 1.25×10^5 Hepa129 cells were injected subcapsularly into the left liver lobe via laparotomy. Treatment started six days later and followed the schedule described above. In the PM299L model, orthotopic tumors were generated by subcapsular injection of 5×10^4 PM299L cells into the left liver lobe and treatment began four days post-implantation. For both models, mice were euthanized after the sixth dose of AZ-505, and

liver samples were collected. Tumor burden was determined using caliper measurements with the formula: $(\pi/6) \times \text{length} \times (\text{width})^2$ for Hepa129 tumors and by tumor weight for PM299L tumors. Ascites grade was also assessed in the Hepa129 model.

Synergy between AZ-505 and anti–PD-1 was evaluated using the fractional tumor volume (FTV) method. For each treatment group, tumor volume was normalized to the mean tumor volume of the control group to obtain the FTV. Expected FTV values for the combination were calculated as the product of the FTVs observed with each monotherapy (FTV_expected = FTV_A × FTV_B). A synergy index was then derived as the ratio of the expected to the observed FTV (Index = FTV_expected / FTV_observed). An index > 1 indicates synergistic interaction, ≈ 1 indicates additivity, and < 1 indicates antagonism.[1]

Quantitative real-time PCR

Complementary DNA (cDNA) synthesis was performed from 500 ng of RNA using 200 U of SuperScript II Reverse Transcriptase (Invitrogen, USA) and 500 ng of Oligo(dT) primers. Transcript levels of IFNγ, IL-1β, CCL5, TGFβ, CD11c, CD8, CD4, iNOS, ARG1, CCND1, AXIN2, SP5, c-MYC and LEF1 were quantified by real-time PCR using SYBR® Green (Invitrogen), using primer sequences listed in Supplemental Table S1. PCR cycling conditions consisted of an initial denaturation at 95 °C for 10 min, followed by 45 cycles of 95 °C for 30 s, 60 °C for 30 s, and 72 °C for 1 min. Melt curve analysis was performed by increasing the temperature from 60 °C to 95 °C at a rate of 2 °C/min, recording fluorescence every 15 s. GAPDH and ACTB were used as internal controls for normalization. Relative gene expression was calculated using the ΔΔCt method and expressed as fold change relative to the control condition. All samples were run in technical triplicates, and a non-template control was included in every assay.

Macrophage stimulation with tumor-derived conditioned media

PM299L and NM53 G03 cells were seeded in 100-mm culture dishes at a density of 2 × 10^6 cells per dish in DMEM supplemented with 10% FBS. After 24 hours, cells were treated overnight with AZ-505 (5 µM) or vehicle (DMSO) in complete medium. The following day, cultures were washed twice with saline solution and incubated with serum-free DMEM for 24 hours. Supernatants were collected and centrifuged at 500 × g for 10 minutes to obtain the conditioned media (CM), which were used immediately or stored at -80 °C. In parallel, J774 macrophages were seeded in 6-well plates at a density of 4 × 10^5 cells per well in RPMI-1640 medium supplemented with 10% FBS. After 24 hours, macrophages were incubated for 20 hours with a 1:4 dilution of CM obtained from PM299L or NM53 cells (± AZ-505). Where indicated, co-treatments with the Wnt pathway modulators CHIR99021 (10 µM, GSK3 inhibitor) or DKK1 (200 ng/mL, Wnt inhibitor) were included during CM exposure. Control macrophages were incubated with fresh medium under the same conditions.

Isolation of splenocytes and tumor-infiltrating cells

Spleens and tumors were collected from PM299L tumor–bearing mice treated with vehicle, AZ-505, anti-PD-1, or the combination therapy. For splenocytes isolation, spleens were gently dissociated in a petri dish using the plunger of a disposable syringe, filtered through a cell strainer and centrifuged (1500 rpm, 5 min). Tumor lysates were prepared by enzymatic digestion with 0.05% collagenase I (Sigma–Aldrich, USA) at 37 °C for 45 min, mechanical dissociation with a syringe plunger through a 70 µm mesh, and centrifugation (2500 rpm, 5 min, 4 °C). For both preparations, red blood cells were lysed with ACK buffer for 5 min at room temperature, and cells were washed (1500 rpm, 5 min), resuspended in physiological saline, and kept on ice until use.

Statistical analyses

Statistical analyses were conducted using R software version 4.4.2 and GraphPad Prism version 8.0 (GraphPad Software, Carlsbad, USA). Data are presented as mean ± standard error of the mean (SEM). Depending on the distribution of the data, comparisons between groups were performed using either parametric (t-test, ANOVA) or non-parametric tests (Mann–Whitney U test, Kruskal–Wallis test). Normality was assessed using the D'Agostino–Pearson omnibus test. A p-value and FDR below 0.05 were considered statistically significant. Graphs were generated using either Prism or R (*ggplot2* package), and final figures were assembled in Adobe Illustrator (Adobe Systems, USA).

Supplemental figures

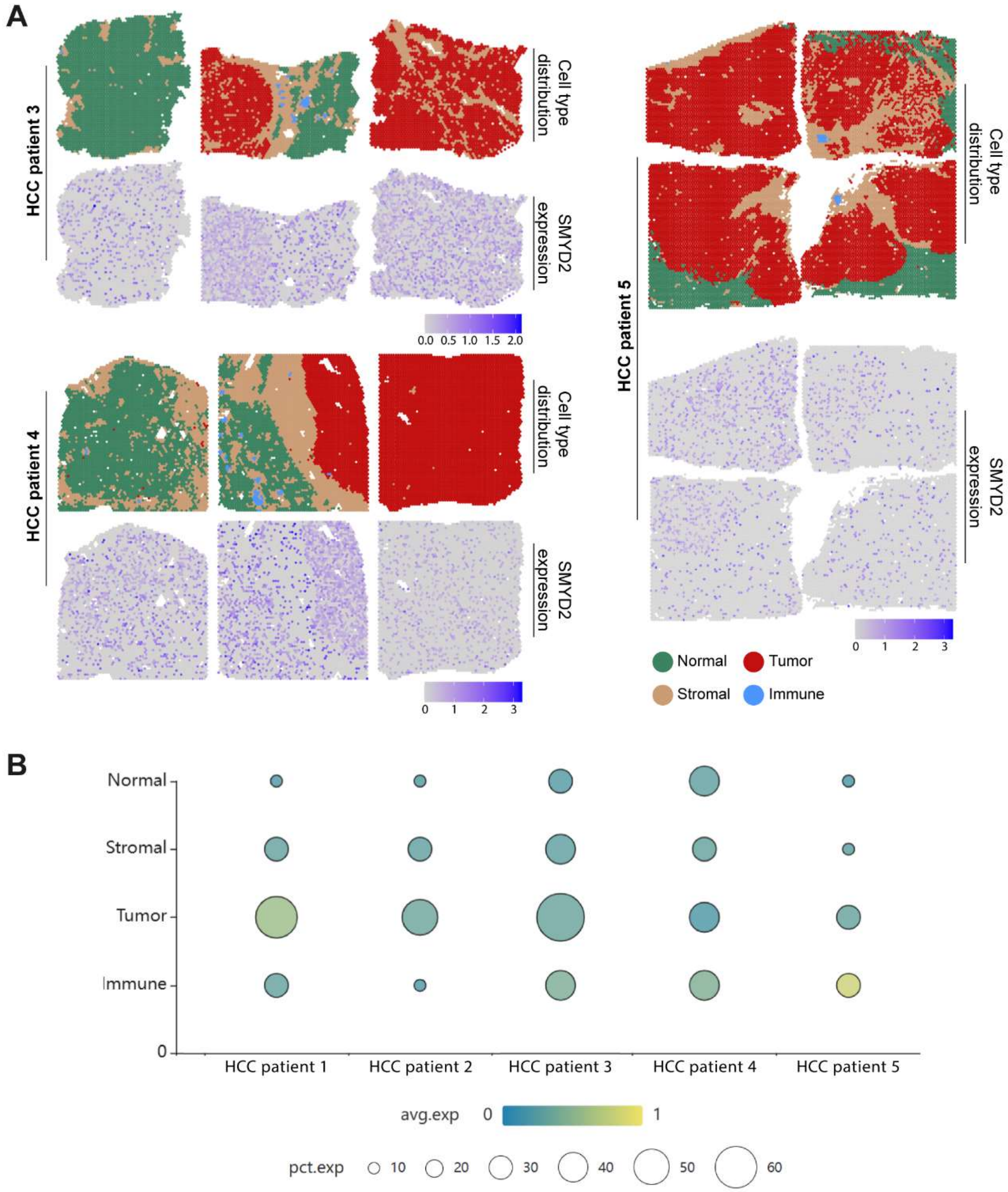

Figure S1. (A) Public spatial transcriptomic data showing SMYD2 expression in liver samples from three HCC patients. Top panels show cell type annotation; bottom panels display SMYD2 expression intensity across spatial coordinates. (B) SMYD2 expression in annotated cell populations. Patient identifiers from the HCCDB v2 database are as

follows: HCC Patient 1 corresponds to HCC-2, Patient 2 to HCC-4, Patient 3 to HCC-1, Patient 4 to HCC-3, and Patient 5 to HCC-4.

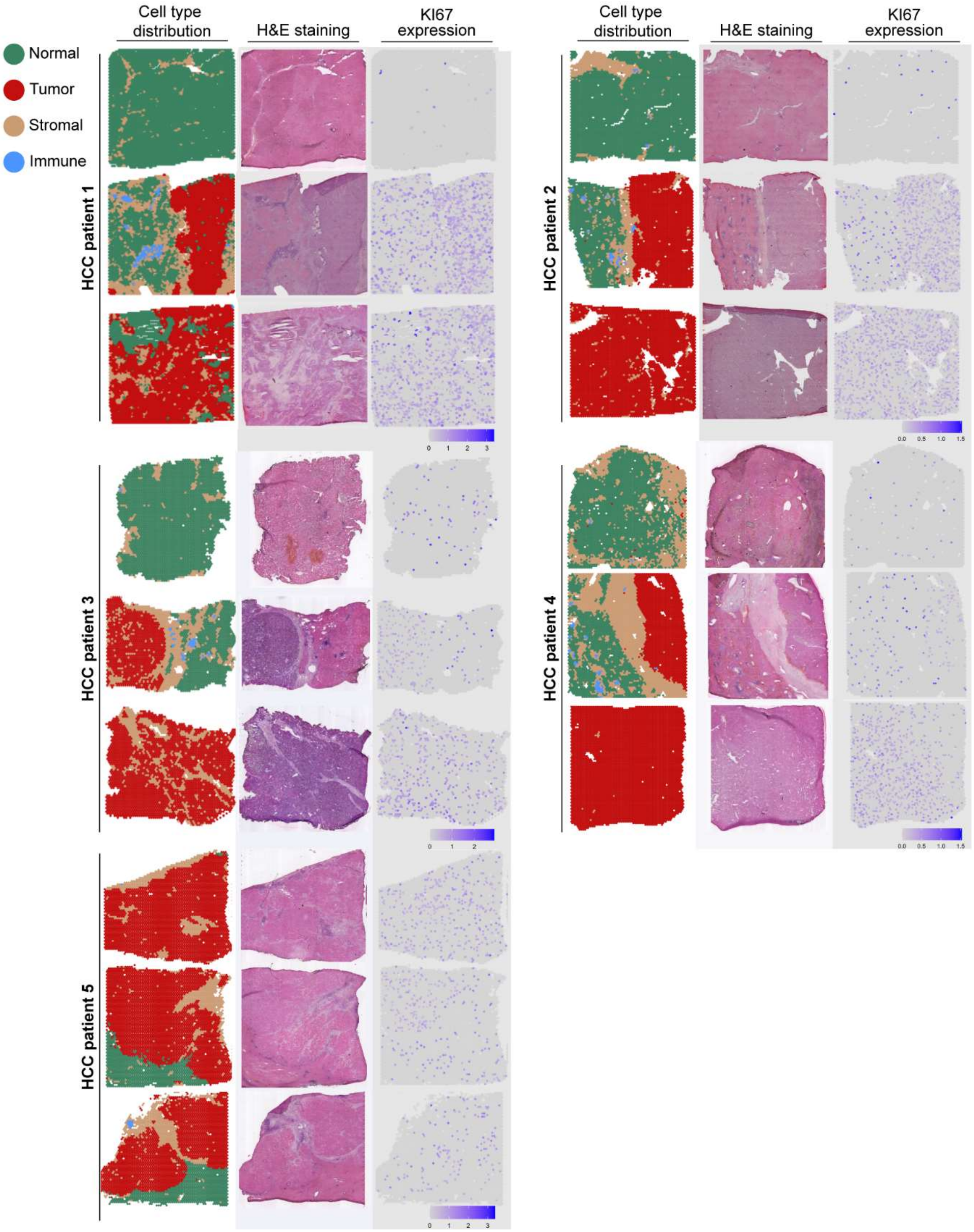

Figure S2. Public spatial transcriptomic data showing cell type annotation (left panels), corresponding H&E staining (middle panels), and Ki67 expression (right panels) in liver samples from HCC patients. Patient identifiers correspond to those described in Figure S1. The cell type distribution panels of patients 1 and 2 were previously presented in Figure 1B, whereas those for patients 3, 4, and 5 were previously presented in Figure S1.

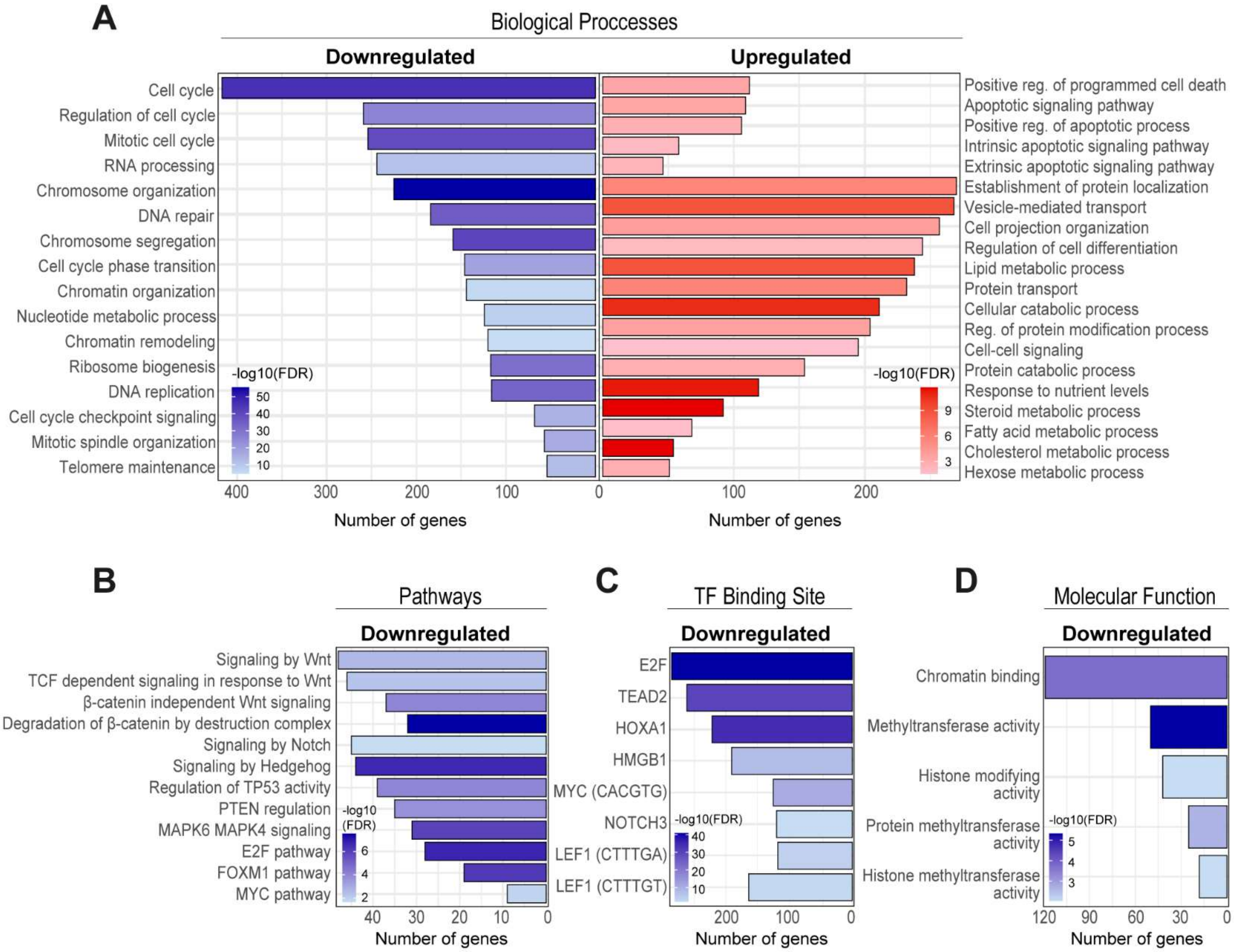

Figure S3. Functional enrichment analysis of DEGs in HuH7 cells treated with LLY-507. GO and pathways analysis of (A) biological processes, (B) pathways, (C) transcription factor binding sites, and (D) molecular function of upregulated or downregulated genes identified by RNA-seq.

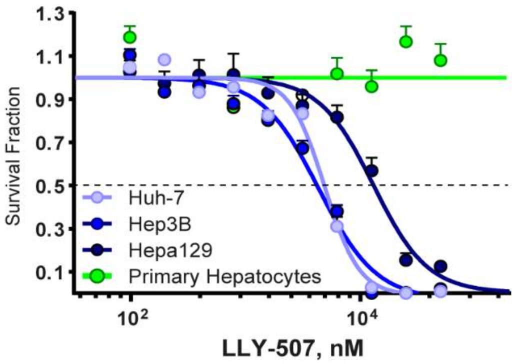

Figure S4. Dose-response curves of human (Huh7, Hep3B) and murine (Hepa129) HCC cell lines, and primary hepatocytes treated with LLY-507 for 72 h. Viability was assessed by MTT. Curves were fitted by non-linear regression and are representative of 2 independent experiments (4 replicates each, shown as mean ± SEM).

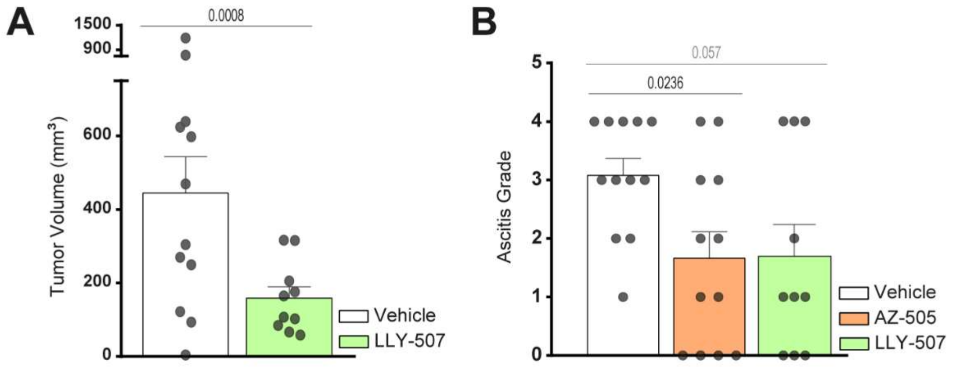

Figure S5. AZ-505 and LLY-507 reduce tumor burden and ascites in an orthotopic Hepa129 HCC model. (A) Tumor volume and (B) ascites grade (assessed visually) at endpoint in C3H/HeN mice bearing Hepa129 tumors treated with vehicle (n = 12), AZ-505 (n = 12) or LLY-507 (n = 10), as described in Fig. 3c. Bars represent mean ± SEM. Statistical analyses were performed using Mann-Whitney (vs. vehicle). Exact p-values are shown in the graphs.

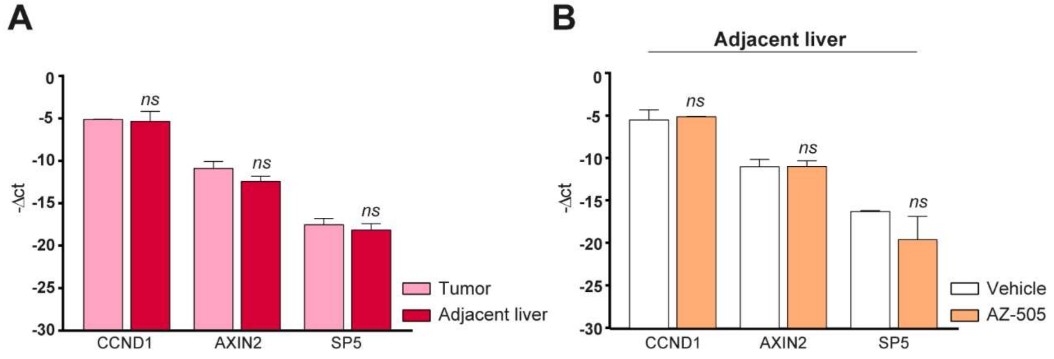

Figure S6. Wnt/β-catenin target gene expression in Hepa129 tumors and adjacent liver tissue. ΔCt values for Ccnd1, Axin2, and Sp5 measured by qPCR in (A) tumors and paired adjacent liver tissue, and (B) adjacent liver tissue from Hepa129-bearing mice treated with vehicle or AZ-505. Bars represent mean ± SEM. ns, not significant (t-test).

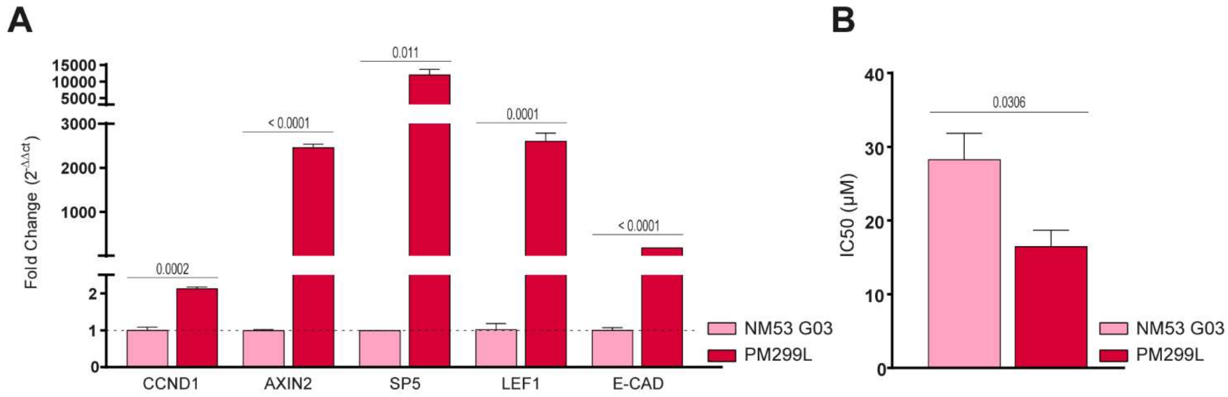

Figure S7. Basal expression of Wnt/β-catenin target genes and sensitivity to AZ-505 in murine HCC cell lines. (A) mRNA expression of Ccnd1, Axin2, Sp5, Lef1, and E-cadherin was assessed by qRT-PCR in PM299L and NM53 G03 cells under basal conditions. Data are shown as fold change (2^−ΔΔCt) relative to NM53 G03. Bars represent mean ± SEM of technical triplicates. (B) IC50 values were determined by MTT assay after 72 h of AZ-505 treatment in PM299L and NM53 G03 cells. Bars represent mean ± SEM of three (NM53 G03) or four (PM299L) independent experiments, each performed in technical quadruplicates. Statistical analyses were performed using t-test (vs. NM53 G03). Exact p-values are shown in the graphs.

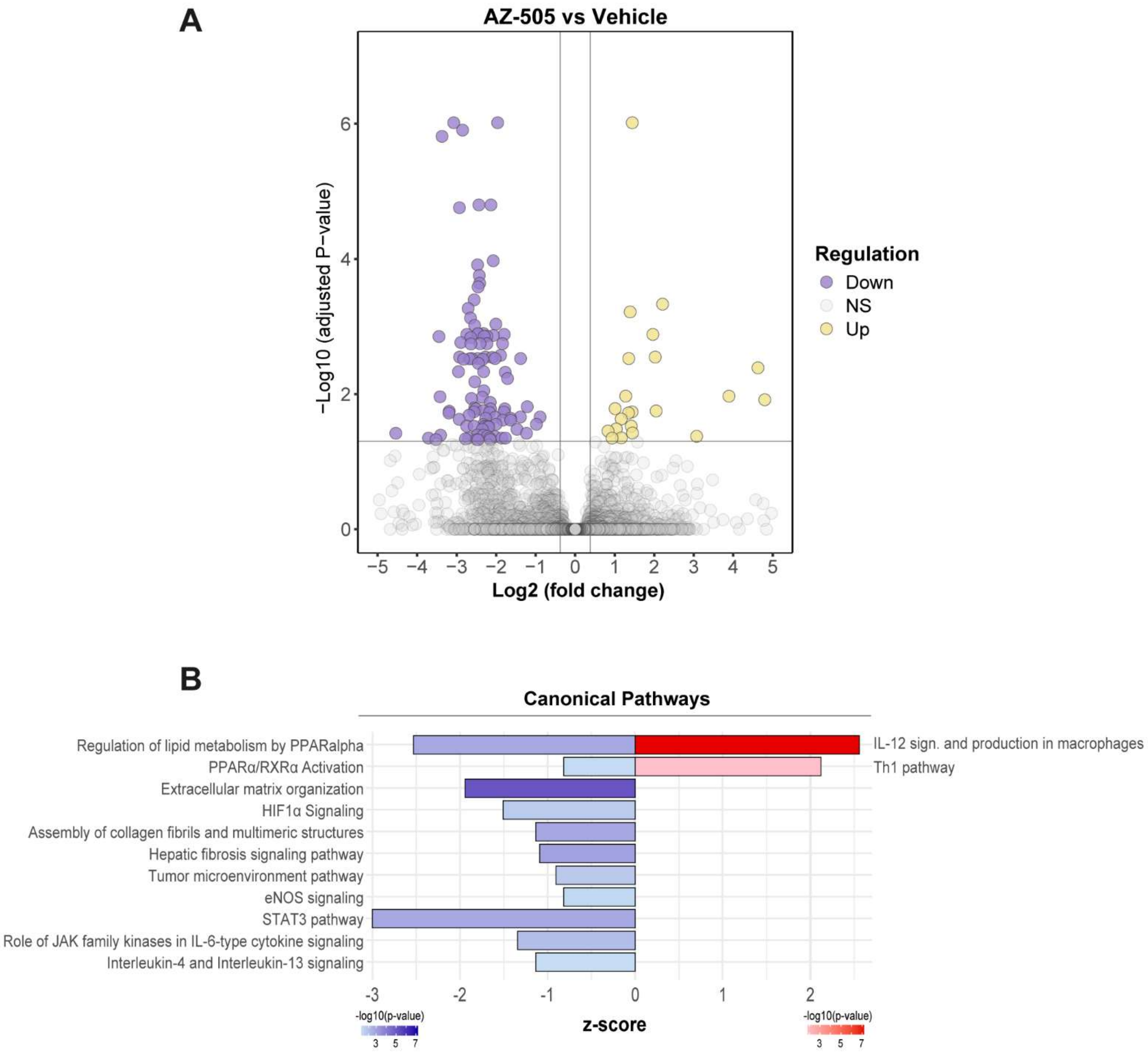

Figure S8. RNA-seq analysis of AZ-505-treated PM299L tumors. (A) Volcano plot showing differentially expressed genes (log2(FC)>|0.378|, FDR<0.05) in PM299L tumors from mice treated with vehicle or AZ-505. Yellow and purple dots indicate significantly up- or downregulated genes, respectively; gray dots indicate non-significant genes. (B) IPA-based pathway analysis of DEGs in PM299L tumors treated with AZ-505 versus vehicle.

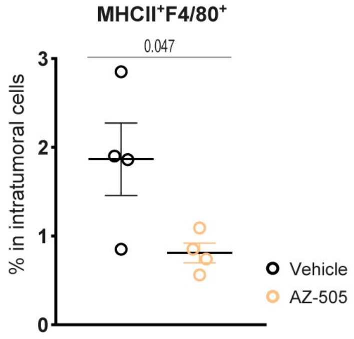

Figure S9. Frequency of MHCII$^+$F4/80$^+$ cells within the intratumoral compartment. Tumors from PM299L-bearing mice treated with vehicle or AZ-505 were dissociated and analyzed by flow cytometry. Bars represent mean ± SEM. Statistical analyses were performed using unpaired t-test (vs. vehicle). Exact p-values are shown in the graphs.

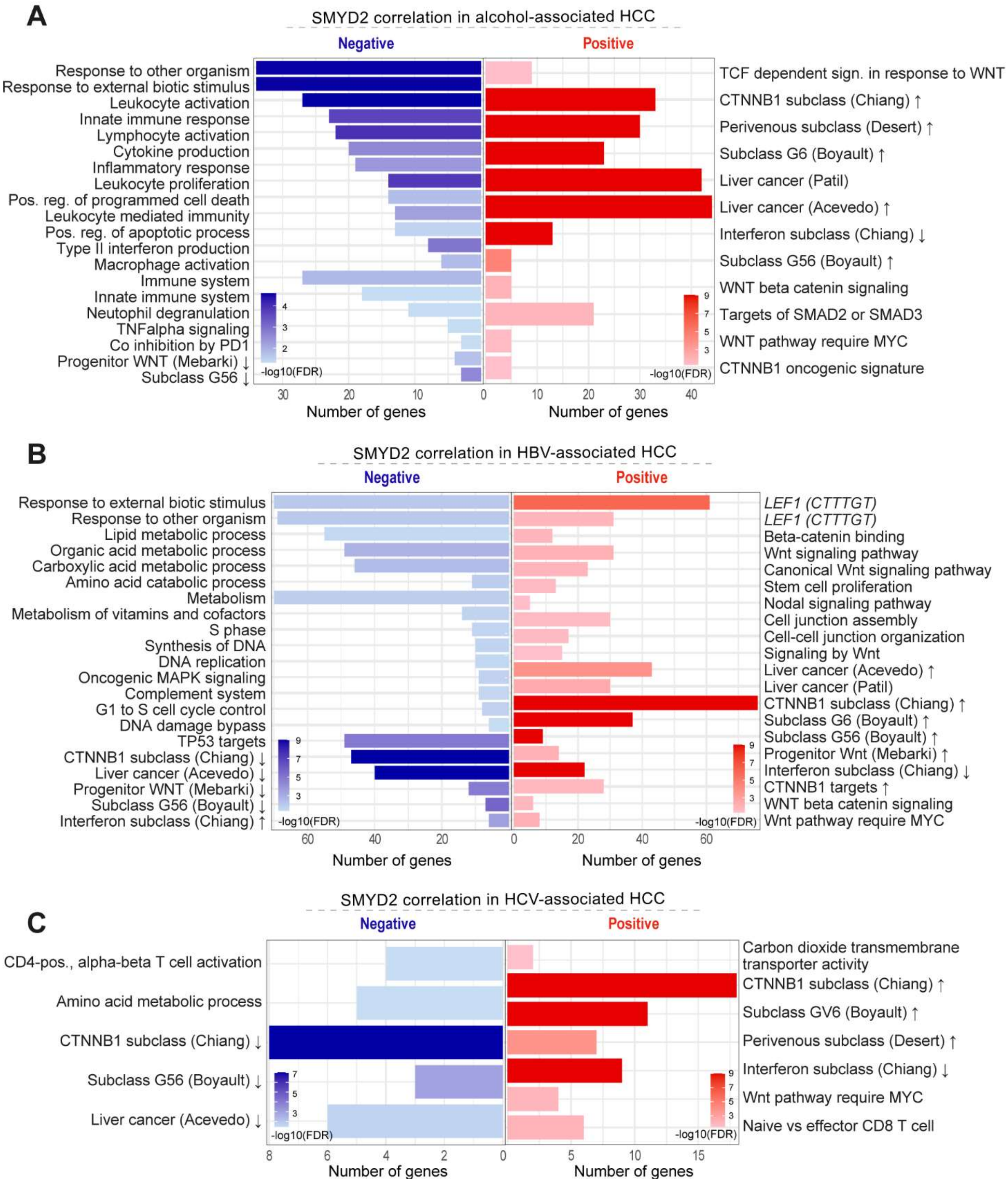

Figure S10. SMYD2-associated transcriptional programs across HCC etiologies. (A–C) Functional enrichment analysis of genes positively (red) and negatively (blue) correlated with SMYD2 expression in TCGA-LIHC patients stratified by etiology: alcohol-associated (A), HBV-associated (B), and HCV-associated HCC (C). Bar plots

show the number of genes contributing to each enriched term, with color intensity reflecting statistical significance (−log10 adjusted p-value) and arrows denoting regulation trends. Terms in *italics* indicate "Transcription Factor Binding Site" annotations; others belong to "Coexpression", "Biological process" and "Pathway" categories.

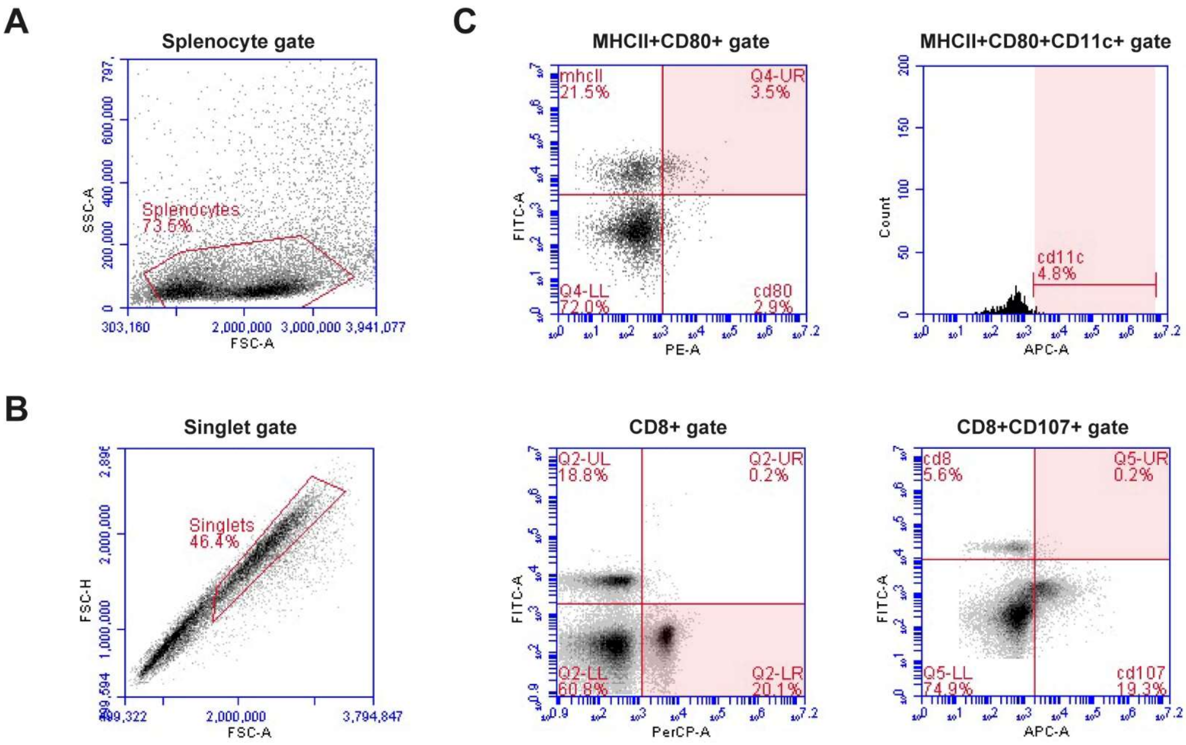

Figure S11. Gating strategy for flow cytometry analysis of splenocytes. (A) Initial gating based on FSC-A vs. SSC-A to select total splenocyte population. (B) Singlets were gated using FSC-A vs. FSC-H, applying a conservative gate to enrich for viable, single cells. (C) Representative dot plots used to define splenocyte subpopulations. Upper left: cells co-expressing MHCII and CD80 were identified. Upper right: within this population, $CD11c^+$ cells were gated. Lower left: $CD8^+$ T cells were identified; CD4 was also included in the staining panel but not analyzed. Lower right: $CD8^+CD107a^+$ cells were quantified from a separate set of splenocytes stimulated ex vivo. Red rectangles indicate the gated populations used for quantification.

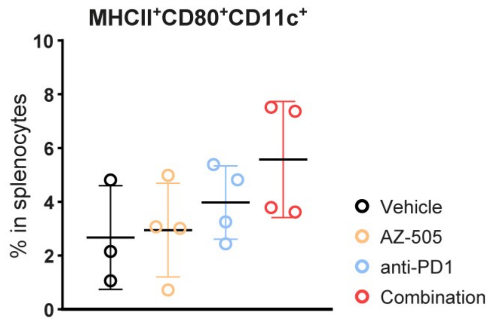

Figure S12. Frequency of splenic MHCII^{+}CD80^{+}CD11b^{+} antigen-presenting cells. Tumors from PM299L-bearing mice treated with vehicle, AZ-505, anti-PD-1, or the combination were dissociated and analyzed by flow cytometry.

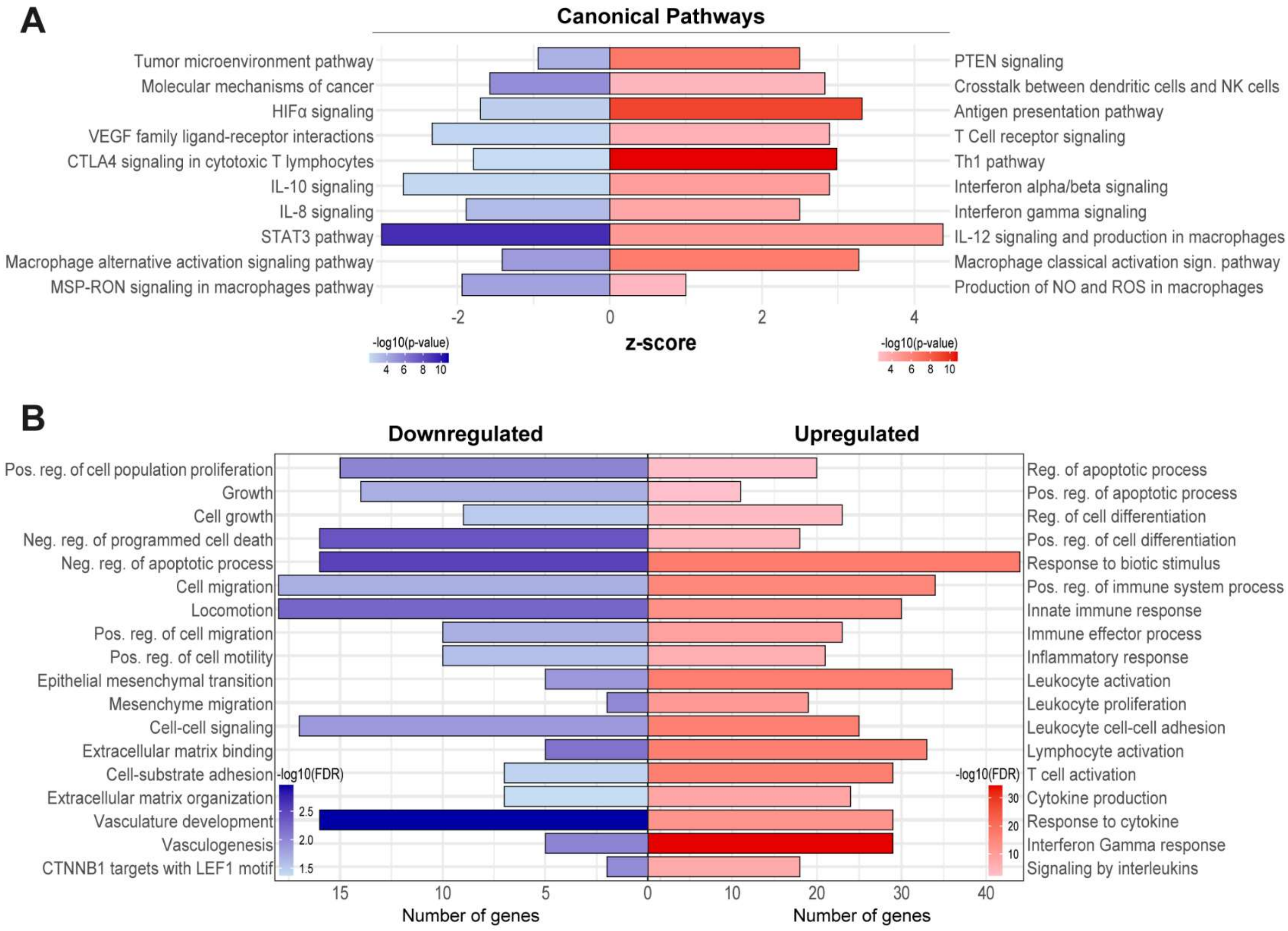

Figure S13. (A) IPA-based pathway analysis of DEGs in PM299L tumors treated with combination therapy versus vehicle. (B) Gene Ontology (GO) enrichment analysis (biological process and pathway categories) of upregulated and downregulated genes.

Supplemental tables

Table S1: oligonucleotides sequences for quantitative real-time PCR assays.

Gene	Forward	Reverse
GAPDH	5'GGGGCTGCCCAGAACATCAT	5'GCCTGCTTCACCACCTTCTTG
ACTB	5'CACTGTCGAGTCGCGTCC	5'CCTTCTGACCCATTCCCACC
SMYD2	5'TGGAATCCTTCGGAGACTGTG	5'CATTACAGTTCCACCTGGGCG
IFNγ	5'GGAACTGGCAAAAGGATGGTGA	5'TGACGCTTATGTTGTTGCTGA
IL-1β	5'TGACAGTGATGAGAATGACCTGTTC	5'TTGGAAGCAGCCCTTCATCT
CCL5	5' CCTGCTGCTTTGCCTACCTCTC	5' ACACACTTGGCGGTTCCTTCGA
TGFβ	5'ACCAACTATTGCTTCAGCTC	5'TGTTGGTTGTAGAGGGCAAG
CD11c	5'ACGTCAGTACAAGGAGATGTTGGA	5'ATCCTATTGCAGAATGCTTCTTTACC
CD8	5'CCGTTGACCCGCTTTCTGT	5'CGGCGTCCATTTTCTTTGGAA
CD4	5'AGGTGATGGGACCTACCTCTC	5'GGGGCCACCACTTGAACTAC
iNOS	5'AAGATGGCCTGGAGGAATGC	5'TGCTGTGCTACAGTTCCGAG
ARG1	5'CAGAAGAATGGAAGAGTCAG	5'CAGATATGCAGGGAGTCACC
CCND1	5'TCAAGTGTGACCCGGACTGC	5'CCTTGGGGTCGACGTTCTG
AXIN2	5'AATAAGCAGCCGTTCGCGATG	5'AGCCTCCTCTCTTTTACAGCAA
SP5	5'TCCAGACCAACAAACACACCA	5'AGTTTGCCGCTACCCAATCA
c-MYC	5'ATGCCCCTCAACGTGAACTTC	5'GTCGCAGATGAAATAGGGCTG
LEF1	5'GCCACCGATGAGATGATCCC	5'TTGATGTCGGCTAAGTCGCC

References

1. Cantero MJ, Bueloni B, Gonzalez Llamazares L, Fiore E, Lameroli L, Atorrasagasti C, et al. Modified mesenchymal stromal cells by in vitro transcribed mRNA: a therapeutic strategy for hepatocellular carcinoma. Stem Cell Res Ther. 2024 Jul 11;15(1):208.
