# Supplementary material for: Targeting SMYD2 improves immunotherapy response in experimental hepatocellular carcinoma
Source: Mol Ther Oncol. 2026 May 20;34(2):201239. doi: 10.1016/j.omton.2026.201239 (PMC13266103; doi:10.1016/j.omton.2026.201239)
Supplement: Document S2. Article plus supplemental information [file mmc2.pdf]

Molecular Therapy
Oncology
Original Article

Targeting SMYD2 improves immunotherapy response in experimental hepatocellular carcinoma

Bárbara Bueloni,[1,2,3] Mariel Fusco,[2,4,5] Esteban Fiore,[1,2,4] Mariana Malvicini,[2,4,5] María Jose Cantero,[1,2,4] Lucía Lameroli,[1,2,4] Mailín Casadei,[1,2,4] Brian Martinez Ruiz,[1,2,4] Florencia Mercogliano,[4,6] Eva Santamaría,[7,8] Catalina Atorrasagasti,[1,2,4] Josepmaria Argemi,[7,8,9,10] Ali Canbay,[11] Juan Bayo,[1,2,4,7,13] and Guillermo Mazzolini[1,2,4,12,13]

[1]Hepatology and Gene Therapy Program, Instituto de Investigaciones en Medicina Traslacional, CONICET - Universidad Austral, Av. Pte. Perón 1500, B1629AHJ, Pilar, Provincia de Buenos Aires, Buenos Aires, Argentina; [2]Facultad de Ciencias Biomédicas, Universidad Austral, Av. Pte. Perón 1500, B1629AHJ, Pilar, Buenos Aires, Argentina; [3]Spectrum Regenerative (HZ4 Liver Inc.), Miami, FL 33130, USA; [4]Consejo Nacional de Investigaciones Científicas y Técnicas (CONICET), Av. Rivadavia 1917, C1033AAJ, Buenos Aires, Argentina; [5]Cancer Immunobiology Laboratory, Instituto de Investigaciones en Medicina Traslacional (IIMT), CONICET-Universidad Austral, Av. Presidente Perón 1500, Derqui-Pilar B1629ODT, Buenos Aires, Argentina; [6]Laboratorio de Inmunología Tumoral. Instituto de Biología y Medicina Experimental (IBYME), CONICET, Vuelta de Obligado C1428ADN, Buenos Aires 2490, Argentina; [7]DNA and RNA Medicine Division, Applied Medical Research Center (CIMA), University of Navarre, 31008 Pamplona, Spain; [8]Centro de Investigación Biomédica en Red de Enfermedades Hepáticas y Digestivas (CIBER-EHD), Av. Monforte de Lemos, 3-5. Pabellón 11, Planta 0, 28029 Madrid, Spain; [9]Liver Unit, Clinica Universidad de Navarra, 31008 Pamplona, Spain; [10]Instituto de Investigación Sanitaria de Navarra (IdISNA), 31008 Pamplona, Spain; [11]Department of Internal Medicine, Ruhr-Universitat Bochum, 44801 Bochum, Germany; [12]Liver Unit, Hospital Universitario Austral, Universidad Austral, Av. Pte. Perón 1500, Pilar B1629AHJ, Buenos Aires, Argentina

Therapeutic options for hepatocellular carcinoma (HCC) remain scarce and limited by resistance, underscoring the need for novel approaches. The lysine methyltransferase SMYD2 is overexpressed in several cancers and regulates oncogenic signaling through histone and non-histone substrates. However, its role in HCC is incompletely defined. We integrated transcriptomic analyses of patient samples with *in vitro* assays and orthotopic murine models to evaluate the therapeutic potential of SMYD2 inhibition in HCC. *SMYD2* was enriched in intratumoral regions and associated with immunosuppressive and Wnt/β-catenin-driven programs. *In vivo*, SMYD2 pharmacological inhibition with AZ-505 reduced tumor burden and induced pro-inflammatory transcriptional changes. The antitumor activity of AZ-505 was also evident in Wnt/β-catenin-active tumors, which further displayed reduced expression of proliferative and immunosuppressive signatures. *In vitro*, SMYD2 inhibition decreased tumor-cell Wnt ligand transcription and shifted macrophage responses toward reduced *TGF-β* expression, in a pattern consistent with reduced Wnt/β-catenin-associated signaling. Finally, AZ-505 synergized with anti-PD-1 in Wnt/β-catenin-active tumors, expanding cytotoxic T cell and antigen-presenting populations. Transcriptomic analyses of combination-treated tumors showed suppression of Wnt/β-catenin- and TGF-β-pathway-associated signatures alongside activation of immune-stimulatory responses. These results position SMYD2 as a dual regulator of tumor growth and immune suppression, and as a promising target to overcome ICI resistance in HCC.

INTRODUCTION

Hepatocellular carcinoma (HCC) ranks as the third leading cause of cancer-related mortality worldwide.[1] Although prevention strategies have improved, HCC incidence continues to rise globally, with approximately 866,000 new cases reported in 2022.[1,2] Curative therapies such as surgical resection and liver transplantation offer favorable long-term outcomes but are restricted to early-stage disease.[3] In contrast, systemic therapies for advanced HCC provide modest survival benefits, with median overall survival ranging from approximately 12 to 24 months.[4] Immune checkpoint inhibitors (ICIs) have emerged as a breakthrough in oncology, yet their efficacy in HCC remains modest, with objective response rates typically below 30%.[5–9] One of the challenges

Received 20 January 2026; accepted 15 May 2026;
https://doi.org/10.1016/j.omton.2026.201239.

[13]Senior author

Correspondence: Juan Bayo, Instituto de Investigaciones en Medicina Traslacional, Facultad de Ciencias Biomédicas, CONICET-Universidad Austral, Avenida Perón 1500, Derqui, 1629, Buenos Aires, Argentina.
E-mail: jbayofina@austral.edu.ar
Correspondence: Guillermo Mazzolini, Instituto de Investigaciones en Medicina Traslacional, Facultad de Ciencias Biomédicas, CONICET-Universidad Austral, Avenida Perón 1500, Derqui, 1629, Buenos Aires, Argentina.
E-mail: gmazzoli@austral.edu.ar

© 2026 The Authors. Published by Elsevier Inc. on behalf of The American Society of Gene and Cell Therapy.
This is an open access article under the CC BY license (http://creativecommons.org/licenses/by/4.0/).

in improving immunotherapy outcomes is the lack of clinically validated biomarkers to predict treatment response.[3,10,11] However, increasing evidence suggests that the activation of certain signaling pathways, including Wnt/β-catenin, TGF-β, and PI3K/Akt/mTOR, is associated with immune-suppressive microenvironments that contribute to resistance to ICIs.[10,12–19] Accordingly, while several first-line therapies already combine ICIs with other agents, including anti-VEGF and tyrosine kinase inhibitors, ongoing research is exploring novel strategies to modulate these pathways alongside immunotherapy, aiming to improve clinical outcomes.[10,17] However, clinical responses remain modest, underscoring the urgent need for novel therapeutic approaches for advanced HCC.

SMYD2 (SET and MYND domain-containing protein 2), a lysine methyltransferase overexpressed in several cancers, including HCC,[20–22] has gained interest as a potential therapeutic target. It was initially characterized as an epigenetic modulator that methylates histone H3 at lysines 4 and 36 (H3K4 and H3K36). However, SMYD2 is now recognized to localize predominantly in the cytoplasm, where it methylates a broad range of non-histone proteins, including p53, PTEN, STAT3, RB1, and HSP90.[21,23] Many of these non-histone targets are frequently altered in HCC, yet they remain challenging to exploit therapeutically,[24] underscoring the relevance of enzymes that regulate their activity. As a central upstream regulator of multiple signaling pathways, SMYD2 modulates key oncogenic processes such as cell proliferation, apoptosis, DNA damage response, and stress signaling.[23,25–29] For example, SMYD2-mediated methylation of p53 at K370 and pRb at K860 impairs their tumor-suppressive activities, reinforcing oncogenic proliferation and survival.[21] This broad array of substrates suggests that targeting SMYD2 may influence multiple oncogenic pathways simultaneously, an attractive strategy in a highly heterogeneous disease such as HCC.[3] Moreover, the development of selective small-molecule inhibitors of SMYD2 has further enabled mechanistic studies and opened avenues for exploring its potential as a therapeutic target.[21] However, the precise role of SMYD2 in HCC remains insufficiently understood, with prior research primarily focusing on its intrinsic functions within tumor cells. Epigenetic regulators are increasingly recognized as relevant modulators of the tumor microenvironment (TME).[30,31] In HCC, histone-modifying enzymes have been shown to influence hepatic stellate cell activation, the polarization of tumor-associated macrophages (TAMs), angiogenesis, and extracellular matrix (ECM) remodeling,[30] thereby contributing to tumor progression and therapeutic resistance. Notably, SMYD2 has been reported to promote hepatic stellate cell activation in liver fibrosis through the upregulation of *Tlr4* expression,[32] directly linking this enzyme to hepatic stromal remodeling. In line with this, computational analyses have associated SMYD2 expression with immune- and stromal-related gene signatures across several cancer types,[33,34] supporting a potential role in shaping the hepatic TME.

This study aims to evaluate the therapeutic potential of SMYD2 inhibition in experimental HCC, assessing both its direct antitumor effects and its potential impact on the TME.

RESULTS

SMYD2 is upregulated in HCC and associates with tumor-specific transcriptional programs

To gain insight into SMYD2 potential as a therapeutic target, we first analyzed publicly available transcriptomic datasets to assess its expression across tumor and non-tumor tissues. We found that *SMYD2* is consistently upregulated in HCC tumors compared to adjacent non-tumoral tissue across 15 independent datasets (Figure 1A), supporting its widespread overexpression in HCC. To further characterize its spatial distribution within tumor-bearing livers, we analyzed spatial transcriptomic datasets from HCC patients available in the HCCDB v2 repository. *SMYD2* is predominantly expressed in tumor regions across 5 patients (Figures 1B and S1A), with lower expression in normal, immune, and stromal areas. Quantification across tissue compartments further confirmed this pattern (Figure S1B), highlighting the tumor-associated localization of *SMYD2* in the HCC microenvironment. To provide histological context and support tumor identity, corresponding H&E staining and *Mki67* expression derived from the same datasets are shown in Figure S2.

To assess the tissue specificity of SMYD2 expression beyond the liver, we analyzed its levels in HCC tumors versus a panel of adjacent non-tumoral tissues from multiple organs within the TCGA cohort, including both digestive and non-digestive tissues (Figure 1C). SMYD2 expression was consistently lower in all histologically normal tissues compared with HCC tumors (Figure 1B). This tumor-enriched expression pattern suggests a degree of cancer specificity that may reduce the risk of off-target toxicity when therapeutically targeted.

To complement these analyses, we examined whether *SMYD2* expression was associated with broader transcriptional changes in HCC. We intersected DEGs between tumors and adjacent tissue with those whose expression correlates with *SMYD2* across multiple datasets (Figure 1D). We found a strong overlap between tumor-upregulated genes and those positively correlated with *SMYD2*, while negatively correlated genes largely matched those downregulated in tumors. These results indicate that *SMYD2* expression is closely associated with the transcriptional landscape of HCC, reinforcing its relevance as a potential therapeutic target.

SMYD2 inhibition in HCC cells reverses oncogenic expression programs dysregulated in patients with HCC

To characterize the biological pathways associated with *SMYD2* expression in HCC, we performed GO analysis on genes correlated with *SMYD2* across patient datasets. Genes positively correlated with *SMYD2* are enriched in pathways characteristic of highly proliferative cells such as biosynthetic and nuclear processes, including translation, ribonucleoprotein complex biogenesis, RNA metabolic activity and histone lysine methylation (Figure 2A).[35–37] In contrast, negatively correlated genes are enriched in pathways related to programmed cell death, antitumor immune responses involving leukocyte-mediated immunity, cytokine signaling and macrophage activation, and hepatic metabolic functions such as

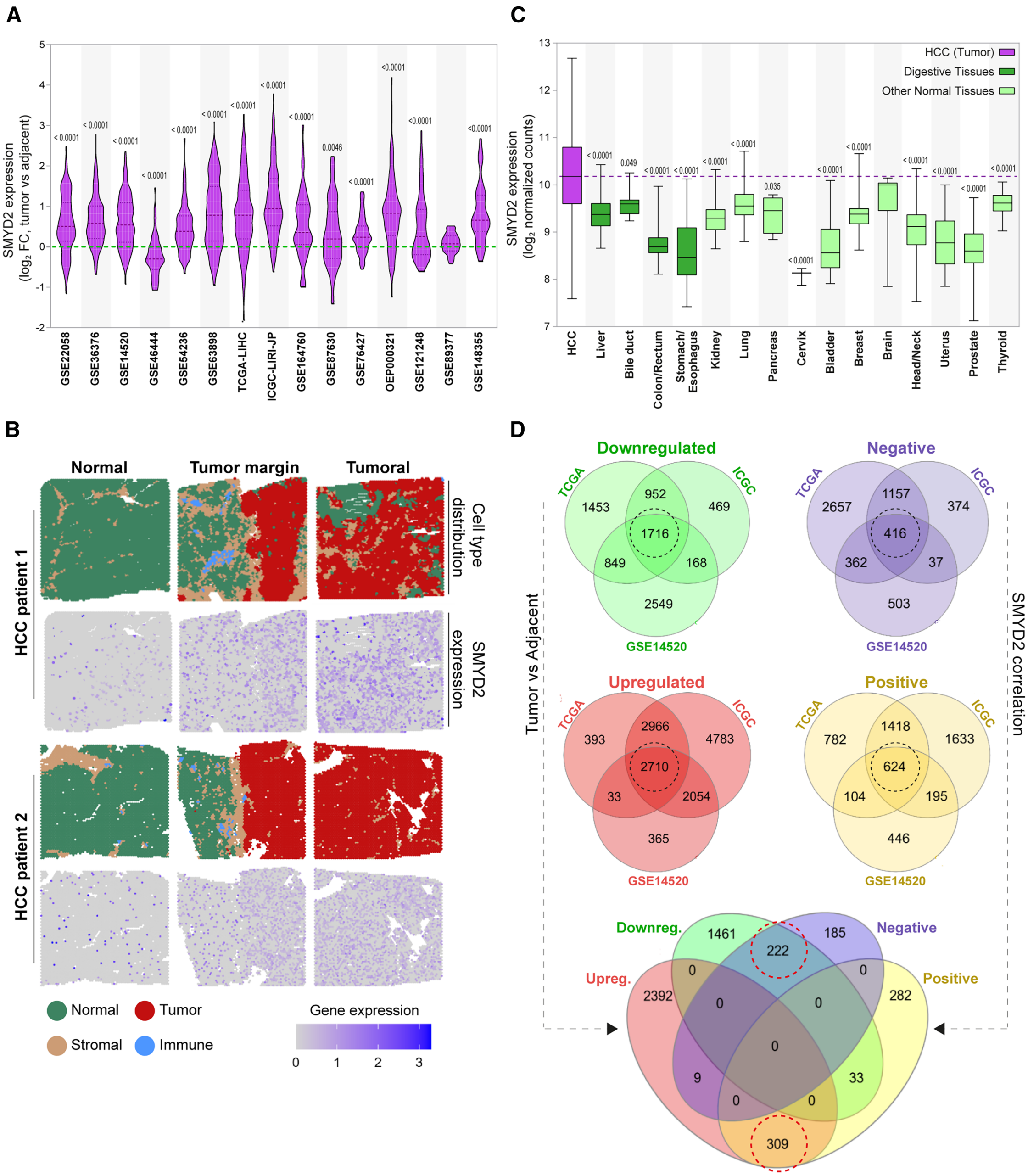

Figure 1. *SMYD2* is overexpressed in HCC, enriched in tumor regions, and correlates with dysregulated transcriptional programs

(A) Violin plots of *SMYD2* expression in tumor relative to adjacent tissue (log_2 fold change) across HCC datasets.

(B) Representative spatial transcriptomic maps from publicly available data of two patients illustrating *SMYD2* expression and cell type distribution. Samples labeled as HCC Patient 1 and HCC Patient 2 correspond to HCCDB v2 samples HCC-2 and HCC-4, respectively.

(C) Boxplots of *SMYD2* expression (log_2-normalized) in HCC compared to non-tumor tissues from digestive and non-digestive organs from TCGA; dashed line indicates median expression in HCC.

(D) Venn diagrams showing overlap between DEGs in HCC vs. adjacent liver and genes correlated with *SMYD2* across TCGA, ICGC, and GSE14520.

Box plots show the median (center line), interquartile range (box), and minimum-to-maximum values (whiskers). Statistical analyses were performed using Wilcoxon signed-rank (vs. adjacent tissue, A) and Mann-Whitney or unpaired t test (vs. normal tissues, C). Exact p-values are shown in the graphs.

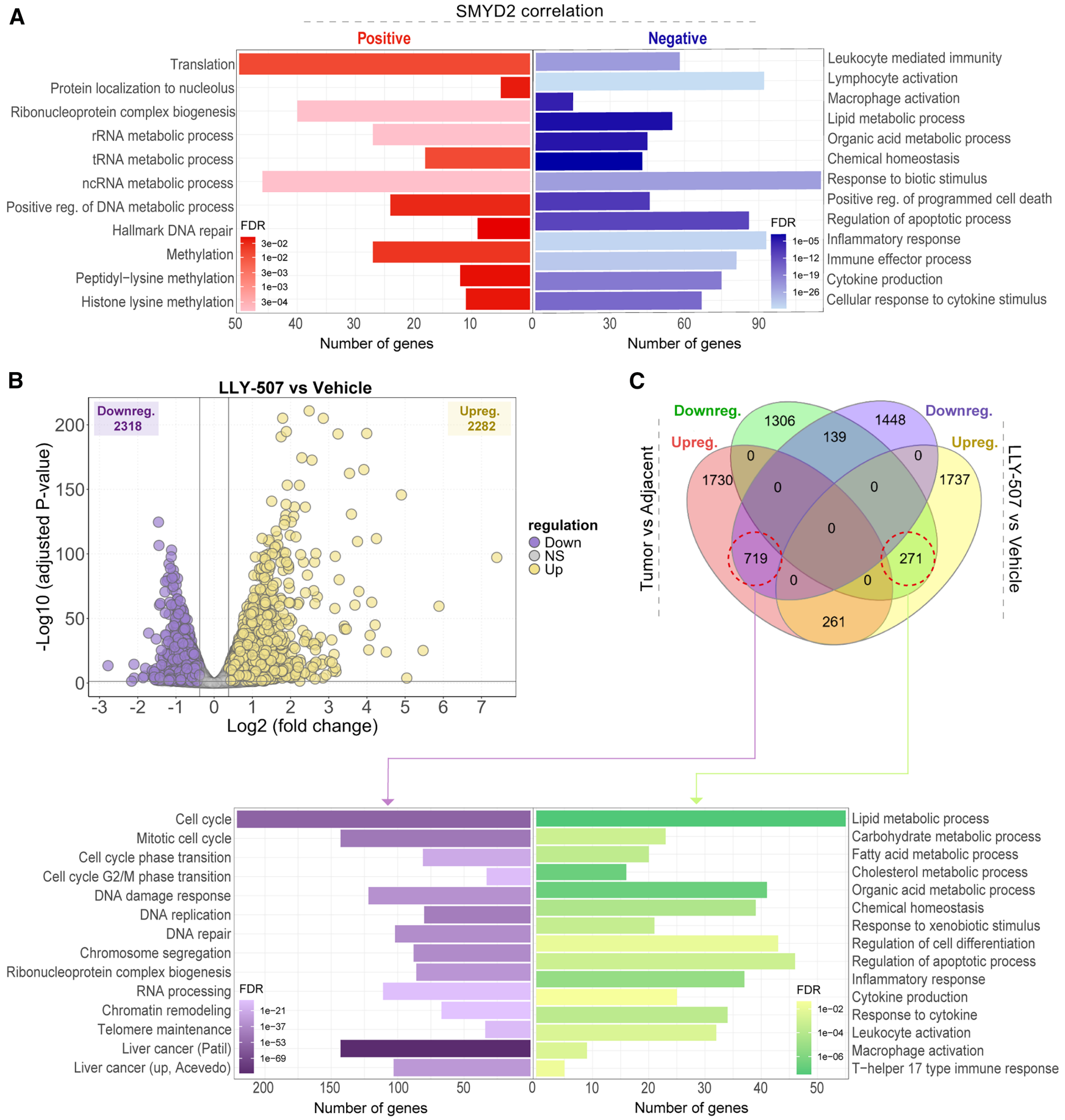

Figure 2. *In vitro* SMYD2 inhibition partially reverses oncogenic transcriptional programs in human HCC cells

(A) GO analysis of genes positively and negatively correlated with *SMYD2* expression in HCC datasets (TCGA, ICGC, and GSE14520).

(B) Volcano plot of DEGs in HuH7 cells treated with LLY-507 (10 μM, 24 h, $log_2FC > |0.378|$, FDR <0.05). Upregulated genes are shown in yellow, downregulated in purple, and non-significant genes in gray.

(C) Venn diagram displaying overlap between genes modulated by LLY-507 and those dysregulated in HCC. The lower panel shows functional enrichment analysis of genes upregulated in HCC and downregulated by LLY-507 (left) or downregulated in HCC and upregulated by LLY-507 (right).

lipid metabolism and chemical homeostasis.[38] These findings suggest that elevated *SMYD2* expression may contribute to the repression of immune surveillance, apoptosis, and normal hepatocyte differentiation programs in HCC.

To further explore SMYD2-associated transcriptional programs in experimental models, we performed RNA-seq analysis on HuH7 cells treated with the SMYD2 inhibitor LLY-507. After 24 h of treatment, a broad transcriptional reprogramming was evident, with 2,282 genes significantly upregulated and 2,518 downregulated (Figure 2B). GO analysis of upregulated genes revealed enrichment in pro-apoptotic pathways and in metabolic processes associated with differentiated liver function, including lipid and nutrient metabolism, vesicle transport, and cell differentiation (Figure S3A). Conversely, downregulated genes were enriched in oncogenic biological processes, such as cell cycle progression, DNA replication

and repair, chromatin remodeling, and RNA processing. These changes were accompanied by the suppression of major tumor-promoting signaling cascades, including the MAPK, FOXM1, E2F, and MYC pathways (Figure S3B), and by the enrichment of genes containing binding motifs for E2F and MYC, among others (Figure S3C). Moreover, GO molecular function analysis identified significant enrichment in terms related to chromatin binding and histone methyltransferase activity (Figure S3D).

To investigate whether these transcriptional changes reflect clinically relevant programs, we intersected genes modulated by LLY-507 with those differentially expressed between tumoral and matched non-tumoral liver tissue in HCC cohorts. Notably, 719 genes overexpressed in human HCC were downregulated by LLY-507 in HuH7 cells and are associated with cell cycle control and DNA metabolism (Figure 2C). In contrast, 271 genes with reduced expression in tumors were upregulated upon SMYD2 inhibition, recapitulating the metabolic and immunological programs previously found to be negatively associated with *SMYD2* expression.

Collectively, these results indicate that SMYD2 is associated with oncogenic, immunosuppressive, and dedifferentiated transcriptional states in HCC. Its pharmacological inhibition disrupts these malignant programs and partially restores expression patterns associated with immune competence and functional hepatocytes.

SMYD2 targeting exerts tumor-selective antitumor activity *in vitro*, suppresses tumor growth, and induces immunomodulatory transcriptional changes in HCC

To translate the transcriptomic insights obtained with LLY-507 into *in vivo* studies, we employed AZ-505, a structurally distinct, well-characterized, and highly selective SMYD2 inhibitor with validated *in vivo* activity and pharmacokinetic properties suitable for systemic administration.[22,39–41] *In vitro*, AZ-505 markedly reduced the viability of human (HuH7, Hep3B) and murine (Hepa129) HCC cell lines while sparing primary hepatocytes (Figure 3A). Consistent results were obtained with LLY-507, further supporting a tumor-selective effect (Figure S4).

Based on this selective impact on tumor cell viability, we assessed the therapeutic potential of SMYD2 inhibition in an immunocompetent orthotopic HCC model with underlying fibrosis, generated by injecting Hepa129 cells. In this model, *Smyd2* expression was significantly higher in tumors than in adjacent non-tumoral liver (Figure 3B), in line with patient data (Figure 1A), providing a biologically relevant platform for therapeutic testing. Treatment with AZ-505 resulted in a robust reduction in tumor growth (Figure 3C) and a significant decrease in ascitic fluid (Figure S5B). LLY-507 exerted comparable effects (Figure S5), further supporting that SMYD2 inhibition confers both antitumor efficacy and beneficial impact on tumor-associated complications *in vivo*.

Next, the inverse correlation between *SMYD2* and immune-related gene programs in patient datasets (Figure 2A) prompted us to examine whether its inhibition could affect the tumor immune microenvironment. Tumors from AZ-505-treated mice showed increased mRNA expression of the pro-inflammatory cytokines IFNγ, IL1β, and CCL5, together with reduced levels of the immunosuppressive cytokine TGF-β (Figure 3D). Moreover, mRNA levels of CD11c and CD8 were elevated (Figure 3D), changes suggestive of a more inflammatory and potentially immune-permissive TME.

Considering the central role of TAMs in the immune landscape of HCC, where they frequently display immunosuppressive features that limit effective T cell responses,[30,42–44] we wondered whether SMYD2 inhibition could affect macrophage polarization. Notably, treatment of J774 macrophages with AZ-505 induced transcriptional changes consistent with a pro-inflammatory shift, including upregulation of *IL1β*, *CCL5*, and *iNOS*, together with downregulation of *ARG1* and *TGF-β*, canonical markers of alternatively activated (M2-like) macrophages (Figure 3E).

To assess whether these associations were reflected in patient tumors, we examined the relationship between SMYD2 expression and immune-cell composition through immune deconvolution of three independent HCC transcriptomic datasets. In line with our experimental data, *SMYD2* expression negatively correlated with the estimated abundance of several key immune effector populations, including $CD8^+$ T cells, M1-like macrophages, γδ T cells, and $CD4^+$ memory T cells (Figure 3F). In contrast, positive correlations were observed with monocytes, M0-like macrophages, resting NK cells, resting dendritic cells (DCs), naive $CD4^+$ T cells, and resting mast cells, cell types typically associated with immature or non-effector immune states. The consistency of this pattern across cohorts supports a model in which high *SMYD2* expression aligns with immune states characterized by reduced effector activity. Together, these results support a potential dual role for SMYD2 in sustaining tumor growth and fostering immunosuppressive features within the TME in HCC.

AZ-505 reduces tumor burden and modulates Wnt/β-catenin and immunoregulatory transcriptional programs in HCC

To investigate the mechanisms underlying the immune effects of SMYD2 inhibition, we first analyzed our HuH7 RNA-seq dataset to identify immune-related pathways potentially regulated by SMYD2. Functional enrichment analyses revealed multiple Wnt/β-catenin-related terms and LEF/TCF binding motifs among the most significantly downregulated categories (Figures S3B and S3C). This is consistent with previous reports implicating SMYD2 in the regulation of Wnt/β-catenin components, including APC2 silencing and β-catenin methylation.[45–47] Given the association between Wnt/β-catenin activation and immunosuppression in HCC,[14,15,48] we first assessed whether this pathway is affected by SMYD2 inhibition *in vivo*. In the Hepa129 model, AZ-505 reduced the expression of canonical β-catenin targets in tumors but not in adjacent tissue (Figures 4A and S6), indicating a tumor-restricted repression of β-catenin-associated transcription. β-catenin target

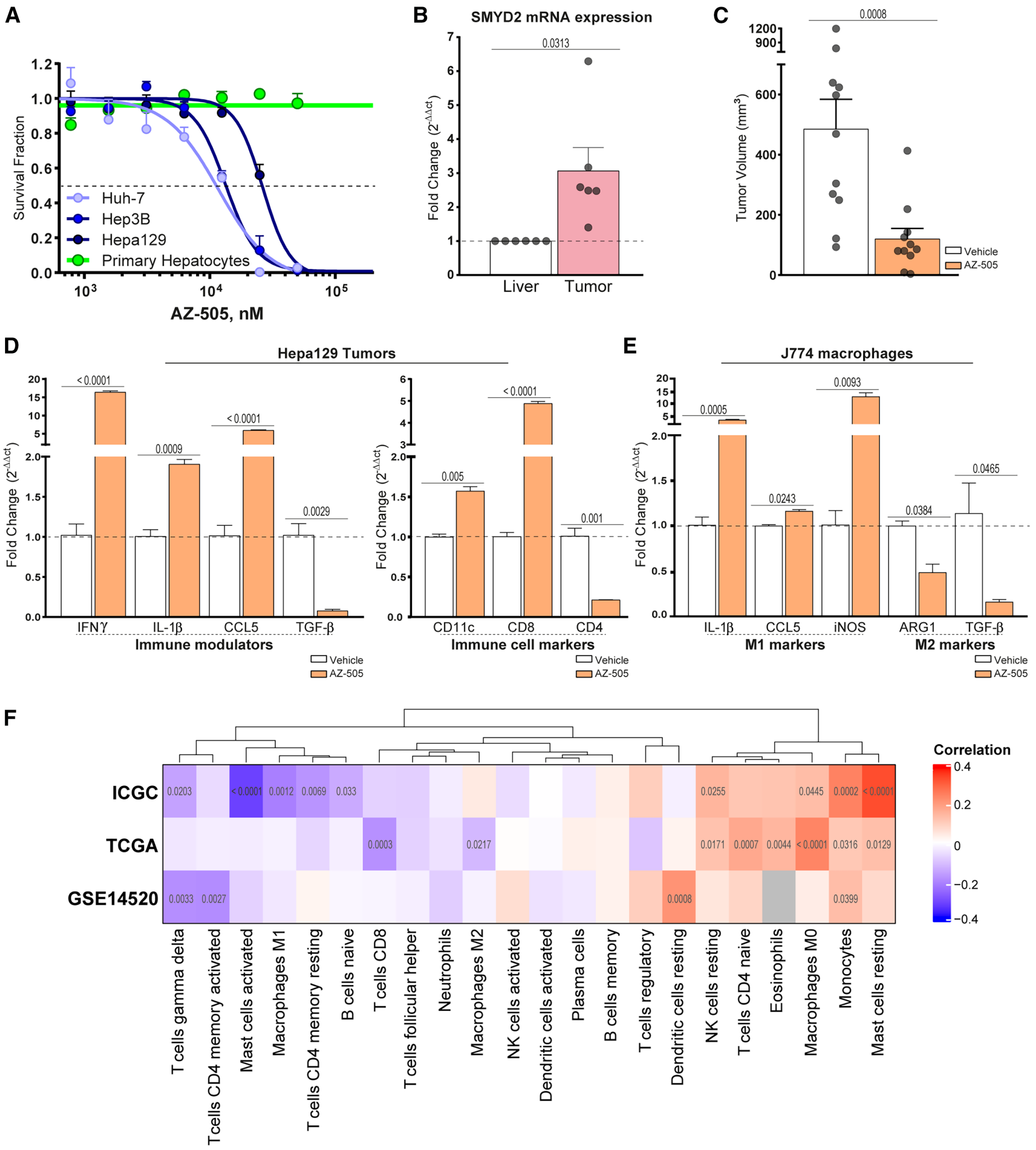

Figure 3. AZ-505 reduces HCC cell viability, decreases tumor burden, and modulates immune-related gene expression *in vitro* and *in vivo*

(A) Dose-response curves of human (HuH7, Hep3B) and murine (Hepa129) HCC cell lines, and primary hepatocytes treated with AZ-505 for 72 h. Viability was assessed by MTT. Curves were fitted by non-linear regression and are representative of 2–3 independent experiments (4 replicates each, shown as mean ± SEM).

(B,C) An orthotopic Hepa129 HCC model was established in fibrotic livers. One week after tumor implantation, mice were treated with AZ-505 (n = 11) or vehicle (n = 11) for six doses over 10 days. b *Smyd2* mRNA levels in paired tumor versus adjacent liver (n = 6), measured by qRT-PCR.

(C) Tumor volume at endpoint.

(D,E) qRT-PCR of (D) immune modulators and immune cell markers in Hepa129 tumors and (E) M1/M2 polarization markers in J774 macrophages treated with AZ-505 (5 μM, 24 h).

(F) Heatmap showing correlations between *SMYD2* expression and immune cell fractions inferred by the MIXTURE algorithm in HCC datasets.

Bars represent mean ± SEM. Statistical analyses were performed using Wilcoxon matched-pairs signed rank (vs. adjacent tissue, B), Mann-Whitney (vs. vehicle, C), unpaired t test (vs. vehicle, D, E), and Spearman's rank correlation (F). Exact p values are shown in the graphs.

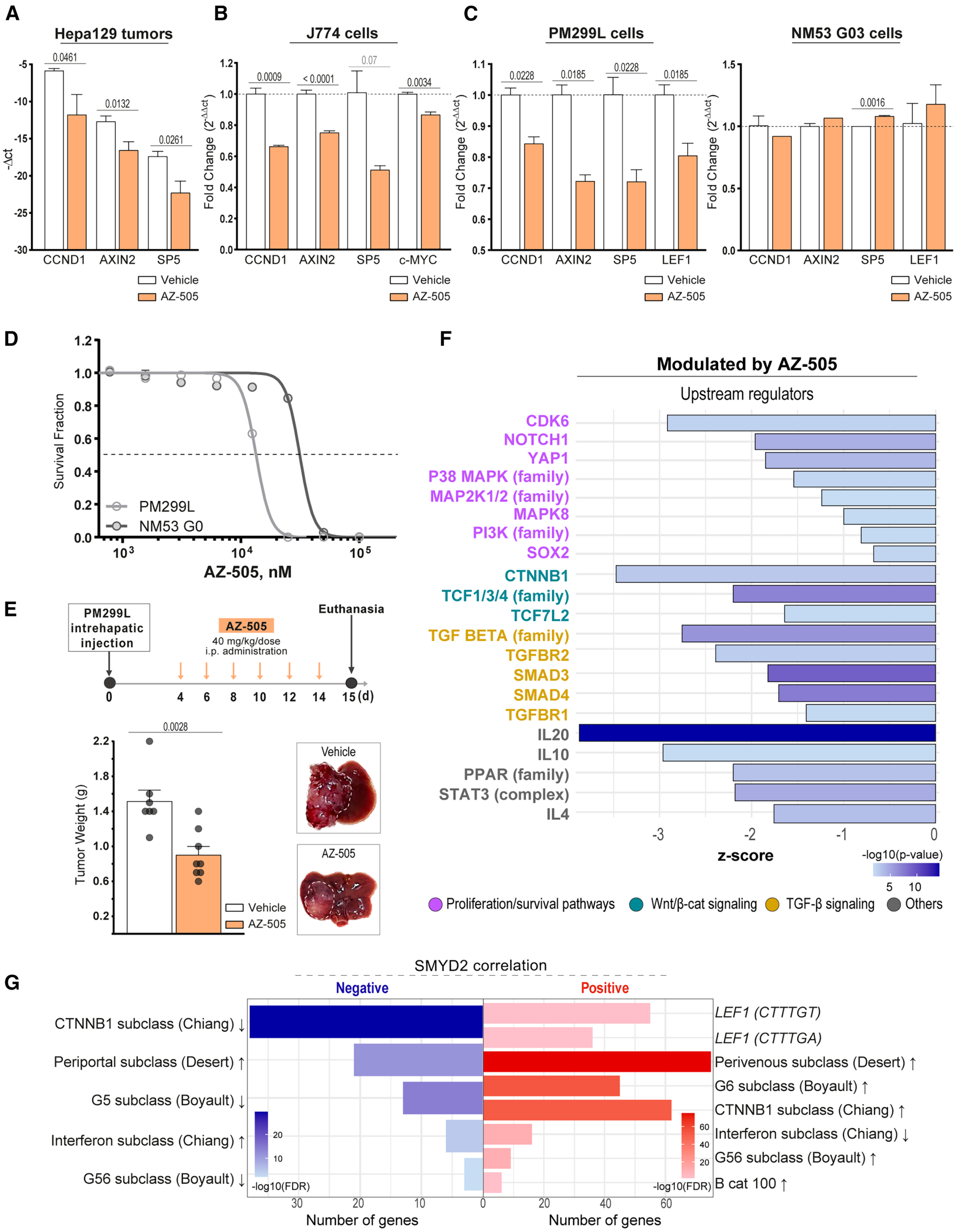

Figure 4. SMYD2 inhibition reduces tumor growth and β-catenin target gene expression in a Wnt/β-catenin-active HCC model, with associated changes in proliferative and immunosuppressive transcriptional signatures

(A–C) qRT-PCR of β-catenin target genes in (A) Hepa129 tumors from mice treated with AZ-505 or vehicle (*n* = 3 per group), (B) J774 macrophages, and (C) PM299L and NM53 G03 HCC cells treated with AZ-505 (5 μM, 24 h). *In vitro* experiments are representative of two independent assays.

(D) Dose-response viability curves in PM299L and NM53 G03 cells after AZ-505 treatment (72 h), assessed by MTT.

(legend continued on next page)

gene repression was also observed in J774 macrophages treated with AZ-505 (Figure 4B), suggesting that SMYD2 inhibition affects both tumor and immune compartments.

To further explore the link between SMYD2 and Wnt/β-catenin, we employed two murine HCC cell lines generated in immunocompetent C57BL/6 mice via hydrodynamic tail vein injection of oncogenic plasmids, as previously described.[15,49] In murine models, a single oncogenic event is typically insufficient to induce hepatocarcinogenesis, requiring synergism with additional driver alterations.[15,50,51] Accordingly, PM299L cells combine a constitutively active β-catenin mutant, modeling Wnt/β-catenin-active HCC, with c-MYC overexpression, a combination that frequently co-occurs in human tumors.[15] Meanwhile, NM53 G03 cells, which retain wild-type β-catenin, also overexpress c-MYC but additionally lack functional p53, one of the most prevalent genetic alterations in HCC.[3,15] These two models thus represent distinct, clinically relevant contexts with differential Wnt/β-catenin activity, providing a suitable framework to assess the pathway-specific effects of SMYD2 inhibition.

Consistent with their genomic background, PM299L cells displayed markedly higher basal expression of β-catenin target genes than NM53 G03 cells (Figure S7A), confirming differential pathway activation. Upon AZ-505 treatment, PM299L cells showed strong downregulation of *Axin2*, *Sp5*, *Ccnd1*, and *Lef1*, while no significant changes were observed in NM53 G03 cells (Figure 4C). Furthermore, PM299L cells were more sensitive to AZ-505 in viability assays, with markedly lower IC_{50} values (Figures 4D and S7B).

To evaluate the *in vivo* therapeutic potential of SMYD2 inhibition in a Wnt/β-catenin-active context, we employed an orthotopic HCC model generated by intrahepatic injection of PM299L cells. This aggressive model exhibited rapid tumor progression, with untreated mice reaching endpoint within 2 weeks. Treatment with AZ-505 significantly reduced tumor weight (Figure 4E), demonstrating robust antitumor activity in tumors bearing Wnt/β-catenin signaling hyperactivation. To gain mechanistic insights, we performed RNA-seq on tumor samples followed by differential expression analysis (Figure S8A). Pathway analysis of DEGs using IPA predicted inhibition of multiple oncogenic and immunosuppressive programs, including STAT3 signaling, IL-4/IL-13 signaling, and ECM remodeling pathways, such as collagen fibril assembly and hepatic fibrosis signaling (Figure S8B). Upstream regulator analysis further identified suppression of key drivers of proliferation and survival, such as CDK6, NOTCH1, YAP1, and members of the MAPK and PI3K families (Figure 4F). Notably, Wnt/β-catenin-related regulators (CTNNB1, TCF1/3/4, TCF7L2) were also among the most inhibited, consistent with the transcriptional effects observed *in vitro*. In addition, immunosuppressive regulators such as TGFβ, IL10, and STAT3 were predicted to be suppressed. Given their established roles in sustaining TAM-mediated immunosuppression in HCC,[43,52,53] we assessed TAM infiltration by flow cytometry. AZ-505 treatment markedly reduced TAM frequency (Figure S9), consistent with a less TAM-permissive microenvironment upon SMYD2 inhibition.

To assess the clinical relevance of these findings, we performed functional enrichment analysis on SMYD2-correlated genes identified in HCC patient transcriptomes. Notably, genes positively correlated with SMYD2 expression were significantly enriched in molecular subtypes associated with Wnt/β-catenin activation and immunosuppression, including the Boyault G5/G6, Chiang *CTNNB1* and perivenous Desert subclasses (Figure 4G). Consistently, these genes were also enriched in the β-catenin (*CTNNB1*) 100 signature and in genes containing LEF1 binding sites, a canonical effector of β-catenin-mediated transcription. In contrast, genes inversely correlated with SMYD2 were enriched in interferon-related subclasses and inflammatory pathways, as well as in gene sets downregulated in G5/G6 and Desert tumors. This inverse enrichment pattern supports a model in which high SMYD2 expression delineates a transcriptional program linked to Wnt/β-catenin activation and immunosuppression, whereas low SMYD2 levels are associated with transcriptional features of immune-active tumors. Notably, these associations were largely preserved across major HCC etiologies (Figure S10), suggesting that the SMYD2-linked transcriptional programs are not restricted to a specific etiological context.

Together, these findings point to SMYD2 as a contributor to proliferative and immunosuppressive programs in HCC, with a potential mechanistic link to Wnt/β-catenin signaling and a particular relevance in molecular subtypes characterized by Wnt/β-catenin hyperactivation and intact p53 function.

SMYD2 inhibition in Wnt-active tumor cells limits paracrine cues and attenuates the induction of TGF-β expression in macrophages

We next investigated how the downregulation of Wnt/β-catenin signaling in tumor cells upon SMYD2 inhibition might influence the immune microenvironment (Figure 5A). In multiple cancer types, Wnt/β-catenin activation reinforces itself through the β-catenin/TCF-dependent induction of Wnt ligands such as WNT3A and WNT5A, which can act in an autocrine and paracrine

(E) Tumor weight at endpoint in PM299L-bearing mice treated with vehicle (n = 7) or AZ-505 (n = 8). Representative gross liver images (ventral view) are shown; dashed lines outline tumor regions.

(F) Upstream regulators inferred by IPA of DEGs in AZ-505-treated tumors vs. vehicle.

(G) Functional enrichment analysis of *SMYD2*-correlated genes across TCGA, ICGC, and GSE14520 datasets, with arrows denoting regulation trends. Terms in *italics* indicate "Transcription Factor Binding Site" annotations; others belong to "Coexpression" category.

Bars represent mean ± SEM. Statistical analyses were performed using unpaired t test (vs. vehicle, A–C) and Mann-Whitney (vs. vehicle, E). Exact p values are shown in the graphs.

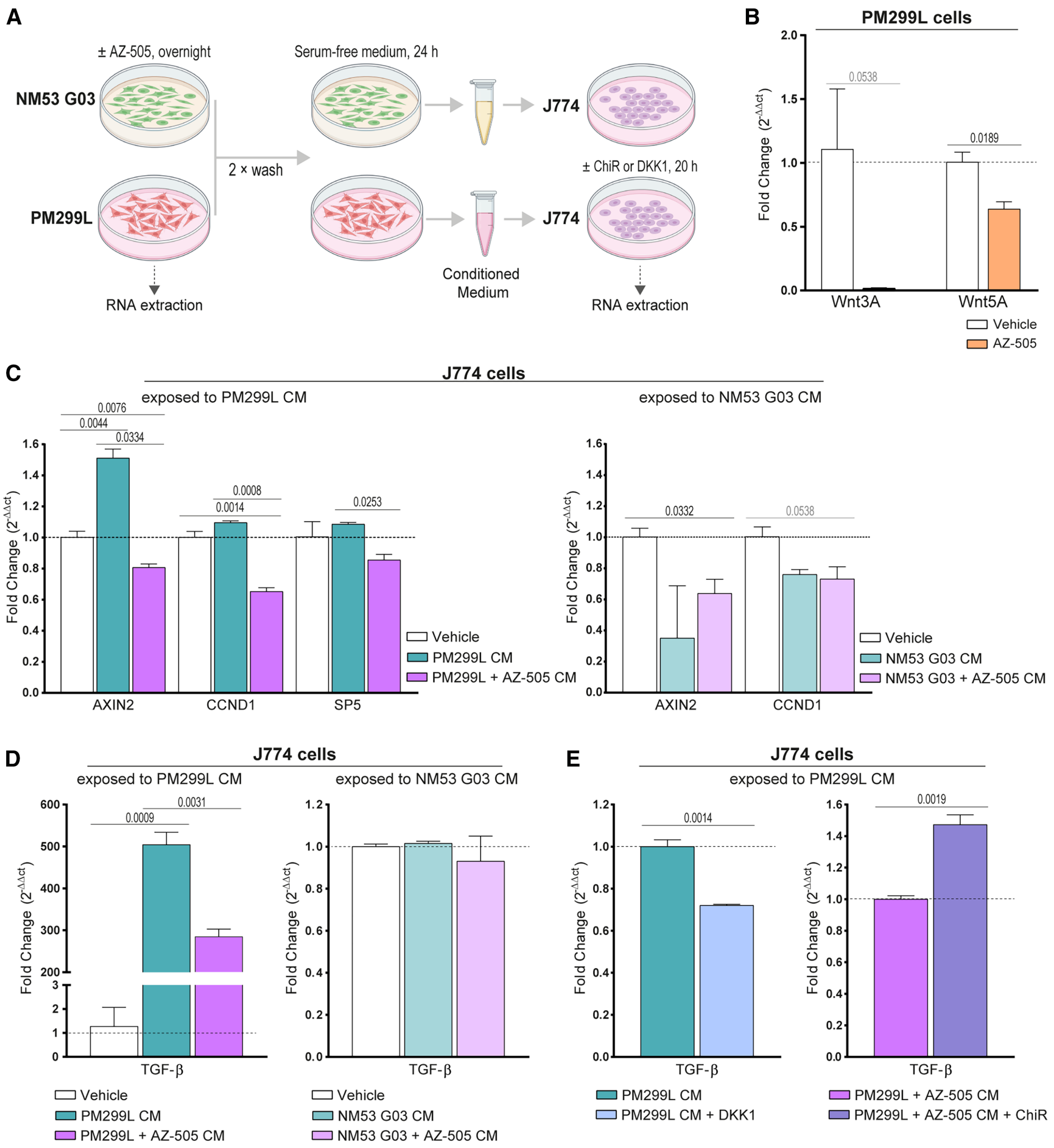

Figure 5. SMYD2 inhibition in Wnt/β-catenin-hyperactive PM299L cells attenuates tumor-driven immunosuppressive macrophage responses

(A) Schematic representation of the experimental design.

(B–E) mRNA expression of (B) Wnt ligands in PM299L cells and of (C) β-catenin targets and (D, E) *TGF-β* in J774 macrophages exposed to CM from PM299L or NM53 G03 cells pretreated with AZ-505 or vehicle, with or without CHIR (Wnt/β-catenin activator) or DKK1 (Wnt/β-catenin inhibitor).

Graphs represent mean ± SEM of one representative of two independent experiments. Statistical analyses were performed using unpaired *t* test (vs. vehicle). Exact *p* values are shown in the graphs.

manner.[54–57] Several of these ligands have been implicated in maintaining an immune-excluded TME, including the induction of immunoregulatory signaling pathways in stromal and immune cells.[58–60] Based on this, we wondered whether SMYD2 inhibition in HCC cells could alter Wnt ligand transcription. In PM299L cells, AZ-505 treatment significantly reduced *Wnt3a* and *Wnt5a* expression (Figure 5B). In line with this, conditioned medium (CM) from untreated PM299L cells increased the expression of Wnt/β-catenin target genes in J774 macrophages (Figure 5C), indicating that soluble tumor-derived factors can activate this pathway in recipient macrophages. This effect was abolished when tumor cells were pretreated with AZ-505, suggesting that SMYD2 activity in tumor cells contributes to the production of such factors. In contrast, CM from NM53 G03 cells, which lack Wnt/β-catenin hyperactivation,

failed to elicit such effects, further linking this response to tumor-intrinsic Wnt/β-catenin activity. Given prior evidence that tumor-derived Wnt ligands can reprogram macrophages toward an immunosuppressive M2-like state in HCC,[58–60] we next wondered whether these CM could influence macrophage phenotype. Remarkably, exposure to CM from PM299L cells led to a pronounced upregulation of *TGF-β* expression in J774 macrophages, a hallmark of immunosuppressive reprogramming (Figure 5D). This effect was impaired when tumor cells were pretreated with AZ-505, whereas CM from NM53 G03 cells had no impact on *TGF-β* expression. These results suggest that the ability of tumor-derived factors to trigger immunosuppressive changes in macrophages is linked to tumor-intrinsic Wnt/β-catenin and SMYD2 activity.

Finally, to assess whether Wnt/β-catenin signaling in macrophages contributes to the observed TGF-β upregulation, we modulated this pathway during CM stimulation. Co-incubation of J774 cells with DKK1, a specific Wnt ligand antagonist, attenuated *TGF-β* expression in response to PM299L-derived CM (Figure 5E, left). Conversely, adding the Wnt/β-catenin activator CHIR-99021 (CHIR) to J774 cells exposed to CM from AZ-505-treated PM299L cells partially restored TGF-β levels (Figure 5E, right). These results support a role for macrophage Wnt/β-catenin signaling activation in the *TGF-β* induction elicited by tumor-secreted factors.

Together, these findings support a model in which SMYD2 contributes to a tumor-macrophage signaling axis involving Wnt ligand production and macrophage Wnt/β-catenin activation, ultimately associated with higher *TGF-β* expression. Disrupting this circuit through SMYD2 inhibition may redirect macrophage responses away from immunosuppression, potentially fostering a more immune-permissive microenvironment.

SMYD2 inhibition synergizes with anti-PD-1 to enhance antitumor response in Wnt/β-catenin-active HCC

Aberrant activation of Wnt/β-catenin signaling has been associated with reduced response to immune checkpoint blockade in HCC,[12–16,19] raising the question of whether SMYD2 inhibition could improve responsiveness to anti-PD-1 in this context. In mice bearing orthotopic PM299L tumors, combining AZ-505 with anti-PD-1 led to the greatest reduction in tumor weight compared to either monotherapy or vehicle (Figure 6A). A synergy index of 1.73, calculated using the fractional product (FTV) method,[61] indicated a synergistic interaction.

To understand the basis of this enhanced response, we analyzed tumors and spleens from treated animals by flow cytometry (Figure S11). Given the observed effects of SMYD2 inhibition on macrophage-associated programs, we hypothesized that these alterations could influence antigen-presenting and T cell-mediated immunity *in vivo*. Interestingly, intratumoral analysis revealed that both anti-PD-1 and combination treatment increased the frequency of $CD8^+$ T cells to a similar extent (Figure 6B), indicating that improved efficacy in the combination group cannot be explained by additional $CD8^+$ T cell accumulation in the tumor. However, only the combination induced systemic immune activation, evidenced by increased frequencies of splenic $CD8^+$ T cells and $MHCII^+CD80^+$ antigen-presenting cells, with a trend toward higher levels of $MHCII^+CD80^+CD11c^+$ DCs (Figure S12). These findings aligned with enhanced cytotoxic potential, as splenocytes from combination-treated mice displayed enhanced $CD8^+$ T cell degranulation upon *ex vivo* stimulation with PM299L cells, as reflected by increased $CD8^+CD107^+$ frequencies (Figure 6B). On the other hand, such functional improvements were absent in either monotherapy group, indicating that the added efficacy of the combination stems from systemic activation and heightened functional competence of $CD8^+$ T cells, rather than further increases in their intratumoral abundance.

To gain deeper mechanistic insight into the effects of the combination treatment, we profiled tumors by RNA-seq. IPA of the 202 DEGs identified predicted robust activation of immune-stimulatory pathways, including IL-12 signaling, Th1 polarization, T cell receptor signaling, interferon-γ signaling, and macrophage classical activation (Figure S13A). At the same time, tumor-promoting and immunosuppressive axes, such as IL-10 signaling, STAT3 signaling, and pathways associated with alternatively activated macrophages, were predicted to be inhibited. Upstream regulator analysis further identified strong activation of pro-inflammatory mediators, including IFNG, IRF1, TNF, IL1B, and TLR4, along with suppression of IL10 and key components of the TGF-β pathway, such as SMAD2, SMAD3, and TGFBR1/2 (Figure 6C). GO and pathway analysis reinforced these findings, showing upregulation of genes involved in T cell activation, leukocyte proliferation, cytokine production, and interferon-γ signaling (Figure S13B). Conversely, downregulated genes were enriched in pathways associated with tumor progression, such as positive regulation of proliferation, epithelial-mesenchymal transition (EMT), vasculogenesis, and ECM remodeling (Figure S13B). In line with the effects observed *in vitro* and in monotherapy, β-catenin (*CTNNB1*) targets with LEF1 motifs were also suppressed, supporting the impact of SMYD2 inhibition on Wnt/β-catenin-driven transcription (Figure S13B).

Reinforcing the translational relevance of these findings, analysis of TCGA and ICGC cohorts revealed that SMYD2 expression inversely correlates with multiple immune-related signatures previously associated with response to anti-PD1 therapy,[62] including antigen-presenting, interferon-γ, inflammatory, and cytolytic activity signatures (Figure 6D). Additionally, the immunophenoscore (IPS) of TCGA HCC samples, obtained from TCIA, was evaluated as a predictor of response to immune checkpoint blockade in patients.[63] This analysis showed that tumors with high SMYD2 expression displayed significantly reduced IPS values compared to SMYD2-low tumors in settings predictive of response to PD-1 blockade ($CTLA4^-/PD1^+$), with a similar trend observed in the $CTLA4^+/PD1^+$ group (Figure 6E). No significant differences were detected under baseline conditions or in CTLA-4 monotherapy settings. These patient-derived data are consistent with a less immunogenic tumor

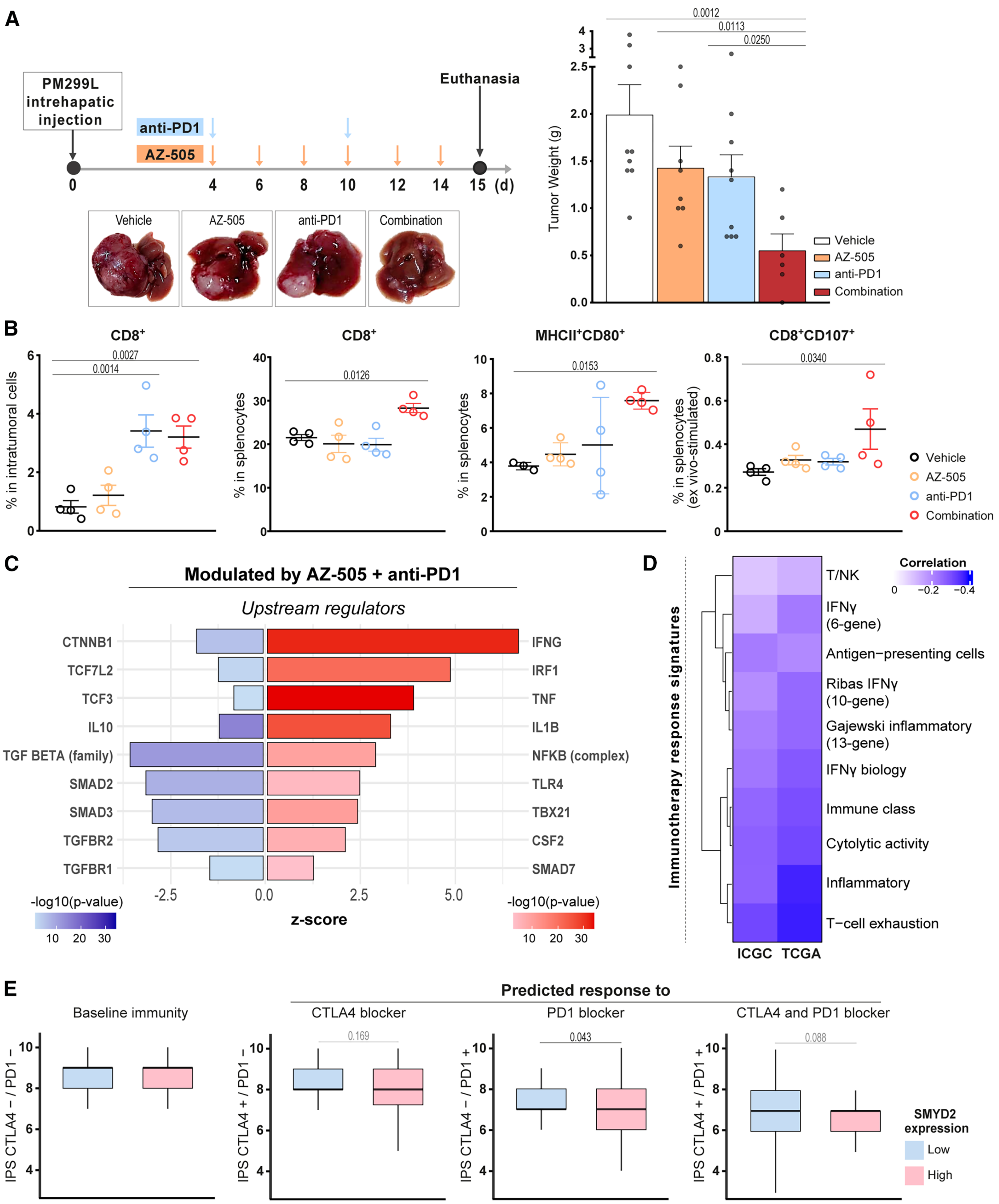

Figure 6. AZ-505 synergizes with anti-PD-1 and increases CD8+CD107+ effector responses in a Wnt/β-catenin-active HCC model

(A) Tumor weight at endpoint in PM299L-bearing mice treated with vehicle ($n = 6$), AZ-505 ($n = 8$), anti-PD-1 ($n = 9$), or combination therapy ($n = 10$). Representative liver images are shown.

(B) Flow cytometry of intratumoral $CD8^+$ T cells; splenic $MHCII^+CD80^+$ antigen-presenting cells; and *ex vivo*-stimulated $CD8^+CD107^+$ T cells. Samples derive from mice described in (A).

(C) Upstream regulators analysis of DEGs from tumors upon combination treatment.

(D) Heatmap showing correlations between SMYD2 expression and immunotherapy response signatures across ICGC and TCGA datasets.

(E) Boxplots of IPS scores obtained from TCIA (TCGA cohort), stratified by SMYD2 expression.

Bars represent mean ± SEM. Statistical analyses were performed using Mann-Whitney (vs. combination group, A; vs. low, E) and one-way ANOVA with Dunnett's multiple comparisons (vs. combination group, B). Exact p values are shown in the graphs.

phenotype associated with elevated SMYD2 expression and further support its link to reduced responsiveness to ICI.

Overall, our findings indicate that SMYD2 inhibition may enhance the immune responsiveness of Wnt/β-catenin-active tumors, enabling anti-PD-1 to elicit a more effective antitumor response.

DISCUSSION

HCC remains a major clinical challenge, with few effective options available for patients with advanced disease. Although ICIs have reshaped management paradigms, objective responses are modest and are largely restricted to a subset of patients.[64–67] These limitations underscore a persistent unmet need for molecularly informed strategies, particularly for tumors harboring features that confer primary or acquired resistance to immunotherapy.

Here, we identify SMYD2 as an oncogenic regulator and potential therapeutic target in HCC. Originally described as a histone methyltransferase, SMYD2 is increasingly recognized to localize mainly to the cytoplasm, where its oncogenic functions appear to be driven primarily by methylation of non-histone substrates.[21,68] When overexpressed, SMYD2 promotes key tumor-associated programs, including cell-cycle dysregulation, apoptosis resistance, EMT, and metabolic reprogramming.[21,22,29,39,69–71] Because these pathways are comparatively quiescent in normal hepatocytes, SMYD2 targeting could be expected to exert preferential effects on malignant cells.

Our data support this notion at several levels. Across multiple patient datasets, *SMYD2* is consistently overexpressed in HCC and shows a predominantly tumor-localized spatial pattern, whereas its expression is lower in multiple histologically normal tissues. Functionally, SMYD2 pharmacological inhibition did not affect viability of primary hepatocytes at concentrations that suppressed HCC cell growth and produced no overt toxicity *in vivo*. These findings, together with prior studies indicating that loss of SMYD2 function mitigates diet-induced steatosis, reduces hepatic lipid accumulation,[72] and limits hepatic stellate cell activation in fibrosis models,[32] support the concept that SMYD2 targeting in HCC may be compatible with underlying chronic liver disease and could display a favorable therapeutic index.

In vivo, we demonstrate that SMYD2 inhibition with AZ-505 exerts a potent antitumoral effect in two orthotopic mouse models. In the fibrosis-associated Hepa129 model, AZ-505 not only significantly reduced tumor burden but also limited accumulation of ascites.[73–75] Supporting translational relevance, inhibiting SMYD2 in HCC cells partially reversed transcriptional programs dysregulated in patients, downregulating proliferative and oncogenic signaling while restoring signatures of differentiated hepatocyte function and immune competence. Several of the affected pathways involve well-known SMYD2 substrates, providing mechanistic plausibility for the observed transcriptional rewiring. The modulation of pathways linked to known SMYD2 substrates (E2F, TP53, STAT3[21,23,29,70]) provides mechanistic plausibility for the observed transcriptional rewiring.

A major aspect emerging from these analyses was the connection between SMYD2 and Wnt/β-catenin signaling. Extensive evidence links aberrant Wnt/β-catenin activation to immunotherapy resistance in HCC.[19,76–78] This pathway is frequently activated in tumors with either immune-excluded phenotypes, marked by scarce T cell infiltration, or immune-exhausted phenotypes characterized by TGFβ-driven dysfunction of infiltrating T cells.[12,13,16,19] However, direct targeting of Wnt/β-catenin remains challenging due to its essential roles in tissue regeneration and homeostasis, raising concerns about potential systemic toxicity.[19,79–82] Our findings suggest that SMYD2 inhibition could represent an alternative approach to modulate Wnt/β-catenin-driven transcription in a tumor-selective manner. Previous studies provide mechanistic context for this observation, showing that SMYD2 can promote β-catenin nuclear accumulation through direct methylation.[83] Additionally, SMYD2 may also modulate the pathway indirectly by repressing APC2, a component of the β-catenin destruction complex, potentially through DNMT1-mediated promoter methylation.[84] In the Hepa129 model, AZ-505 reduced the expression of canonical β-catenin target genes in tumors but not in the surrounding fibrotic liver, despite comparable baseline expression of these targets in both compartments. This pattern suggests that the preferential expression of SMYD2 in tumors may allow selective modulation of oncogenic Wnt/β-catenin signaling while preserving its physiological role in the liver.

Notably, this effect was evident in the Wnt/β-catenin-hyperactive PM299L cells but not in the NM53 G03 counterpart, which lacks both Wnt/β-catenin hyperactivation and functional p53.[15] These cell lines share a common genetic background and MYC-driven transformation, yet displayed differential sensitivity to AZ-505, suggesting that SMYD2 dependency is determined by specific oncogenic alterations. In PM299L cells, SMYD2 likely supports proliferative capacity through repression of p53, one of its best-characterized non-histone substrates,[21,29] while also maintaining Wnt/β-catenin-dependent transcription. Conversely, the absence of both functional p53 and Wnt/β-catenin activation in NM53 G03 cells may reduce their reliance on SMYD2, thereby limiting the impact of its inhibition. This context-specific susceptibility has clinical relevance, as *CTNNB1* and *TP53* mutations occur predominantly in a mutually exclusive manner in HCC.[85–87] Together with the observed *in vivo* response of PM299L tumors to AZ-505, these findings highlight a subset of HCC with coexisting Wnt/β-catenin activation and intact p53 that may be particularly vulnerable to SMYD2-targeted therapies.

Beyond its tumor-intrinsic functions, SMYD2 has been implicated in immune regulation. Previous studies show that its inhibition can enhance antitumor immunity by impairing DNA repair,[88] while its overexpression in macrophages dampens pro-inflammatory activation and cytokine production.[89] Nevertheless, its contribution to the immune landscape of HCC had not been clearly defined. This study reveals that SMYD2 contributes to macrophage immunosuppression through a combination of cell-intrinsic effects and tumor-driven paracrine cues in HCC. We demonstrate that soluble signals

from Wnt/β-catenin-active tumor cells are able to induce a TGF-β-expressing phenotype in macrophages, mediated by Wnt/β-catenin signaling in the recipient cells. Further, we show that this response is mitigated by SMYD2 inhibition in tumor cells. These factors may include the Wnt ligands *Wnt5a* and *Wnt3a, as* their expression is reduced by AZ-505 and they can activate Wnt/β-catenin signaling in multiple immune cell populations, including TAMs, where they predominantly promote immunosuppressive states.[60,78,90–92] These considerations offer a mechanistically coherent explanation for the observed effects.

Importantly, the immunological consequences of these mechanisms were reflected *in vivo*. In both orthotopic models, SMYD2 targeting was associated with the transcriptional downregulation of *TGF-β* and its downstream mediators. However, the overall immunological outcomes varied between models. In Hepa129 tumors, this reduction was accompanied by a shift in the expression of immune mediators, indicative of a more pro-inflammatory TME. Conversely, in PM299L tumors the predominant effect was the attenuation of pathways associated with TGF-β-driven immune exclusion, including those linked to ECM remodeling and collagen deposition,[93–96] together with a reduction in TAM abundance, but without clear evidence of effector activation. In patient cohorts, SMYD2 expression aligned with an immune landscape enriched in immature or non-effector populations and low levels of cytotoxic and proinflammatory cells. Collectively, these findings support a model in which SMYD2 helps sustain a myeloid- and stroma-driven immunosuppressive milieu, particularly in Wnt/β-catenin-active tumors, and suggest that its inhibition may alleviate key barriers that limit responsiveness to immune checkpoint blockade.

Consistent with this rationale, we demonstrated that SMYD2 inhibition enhances the efficacy of anti-PD-1 immunotherapy in Wnt/β-catenin-active tumors. Although the combination did not further increase intratumoral $CD8^+$ T cell abundance beyond anti-PD-1 alone, it induced marked systemic immune activation, with higher frequencies of splenic $CD8^+$ T cells and activated antigen-presenting cells, together with enhanced $CD8^+$ T cell degranulation upon *ex vivo* stimulation. Such systemic activation is consistent with improved effector competence, which could contribute to antitumor activity even without additional $CD8^+$ recruitment to the tumor.

Transcriptomic profiling of combination-treated tumors provided additional mechanistic insight. Predicted activation of IL-12/Th1-associated and macrophage-classical-activation programs is compatible with the increase in splenic APC activation and $CD8^+$ T cell functionality. This is consistent with the well-established role of IL-12 and Th1 cytokines in promoting inflammatory myeloid states and supporting cytotoxic T cell responses.[45–47] Importantly, several of the pathways activated in combination-treated tumors correspond to programs typically suppressed by TAM-derived TGF-β and IL-10 in HCC, an axis linked to immunotherapy resistance.[10,97,98] This pattern suggests that SMYD2 inhibition may help relieve this myeloid-driven immunosuppressive pressure. Consistently, this was accompanied by the suppression of pathways involved in ECM remodeling, STAT3 signaling, EMT, vasculogenesis, and alternative macrophage activation. While these pathways are not direct targets of PD-1 blockade, their suppression reflects the effects of AZ-505 monotherapy, underscoring the non-redundant contribution of SMYD2 inhibition to the remodeling of the TME. Overall, this transcriptional profile suggests a shift away from a TAM-associated immunosuppressive state and toward a more classically activated, pro-inflammatory myeloid compartment, generating a TME more supportive of Th1-skewed, cytotoxic immunity. Together with enhanced systemic T cell activation, these findings suggest that AZ-505 helps alleviate stromal and myeloid barriers, thereby enabling PD-1 blockade to induce more effective cytotoxic T cell responses.

In summary, our findings position SMYD2 as a potential dual-action therapeutic target in HCC, with roles in sustaining tumor growth and shaping an immunosuppressive microenvironment. Pharmacological SMYD2 inhibition was effective as monotherapy and, notably, synergized with immune checkpoint blockade in a Wnt/β-catenin-active, immunotherapy-refractory setting. Supporting clinical relevance, *SMYD2* expression correlated positively with immunosuppressive HCC subtypes, including *CTNNB1*-mutant and Boyault G5/6 tumors, and inversely with immune signatures predictive of ICI responsiveness in patients.[62] Consistently, analysis of IPS in TCGA samples revealed lower predicted responsiveness to PD-1 and combined PD-1/CTLA-4 blockade in tumors with high *SMYD2* expression, whereas no differences were observed under baseline conditions or in CTLA-4 monotherapy settings. It should be noted that IPS represents a composite immunogenomic score rather than a direct measure of clinical response.[63] In this context, the observed pattern suggests that *SMYD2* expression could be primarily associated not with global tumor immunogenicity or with mechanisms regulating T cell priming and early activation, including CTLA4-mediated inhibitory signaling, but instead with immune states that could become relevant during the effector phase of the antitumor response, where PD-1-dependent inhibitory pathways play a central role. This is consistent with our experimental data, in which SMYD2 inhibition enhanced the efficacy of anti-PD-1 therapy without markedly increasing intratumoral $CD8^+$ T cell abundance. Therefore, SMYD2-driven mechanisms within the tumor microenvironment may preferentially impact pathways linked to T cell dysfunction, thereby shaping conditions that are less permissive to PD-1-based immunotherapy.

Through combined effects on tumor-intrinsic Wnt/β-catenin signaling, myeloid immunosuppressive circuits, and systemic T cell activation, SMYD2 inhibition may render Wnt-active HCC more responsive to ICIs, providing rationale to explore SMYD2-targeted strategies in this clinically challenging subgroup. Prospective studies should evaluate whether SMYD2 expression or derived transcriptional signatures can serve as predictive biomarkers to stratify candidates for SMYD2-targeted therapies and optimize rational ICI combinations.

MATERIALS AND METHODS

Bioinformatic analyses

Patient transcriptomic data were obtained from HCCDB v2 and the Firebrowse portal.[99,100] *SMYD2* expression in tumor and adjacent non-tumoral tissues was analyzed. Non-tumoral tissues correspond to histologically non-tumorous liver tissue adjacent to the tumor, obtained from the same patients at the time of surgical resection. The terms ‘normal’ and ‘adjacent non-tumoral’ tissues are used throughout the manuscript to refer to these samples. Gene expression values were log-transformed, and differential expression was assessed in paired tumor-normal samples with false discovery rate (FDR) < 0.05. Correlation analyses were carried out using Pearson’s coefficients. Functional enrichment of differentially expressed genes was performed using ToppGene (ToppFun), and immune cell composition was inferred from bulk transcriptomes using the MIXTURE deconvolution algorithm. Immunophenoscores (IPS) for TCGA-LIHC samples were obtained from The Cancer Immunome Atlas (TCIA), and patients were classified into *SMYD2*-high and *SMYD2*-low groups based on the median expression value. Upstream regulator analyses were conducted using Ingenuity Pathway Analysis (IPA®, Qiagen).

Cell culture, primary hepatocyte isolation, and viability assay

The murine HCC cell line Hepa129 (syngeneic to C3H/HeJ mice) was generously provided by Dr. Volker Schmitz (University of Bonn, Germany). PM299L murine HCC cells (provided by Dr. Lujambio, New York, USA) were originally obtained from C57BL/6 mice after hydrodynamic tail vein injection of pT3-EF1a-Myc-Lu-cOS (encoding c-Myc, luciferase, and the SIYRYYGL, SIINFEKL, and OVA323-339 epitopes), pT3-N90-β-catenin (constitutively active *CTNNB1*), and CMV-SB13 (Sleeping Beauty transposase).[49] The NM53 G03 cell line was developed by delivering pT3-EF1a-Myc (encoding c-Myc and luciferase), px330-sg-p53 (CRISPR-Cas9 system targeting Trp53), and CMV-SB13 into C57BL/6 mice, followed by *ex vivo* culture and *in vivo* passaging, according to the protocol established for the PM299L model.[49] Human HCC cell lines were kindly provided by Dr. Ian Corbin (UT Southwestern Medical Center, USA). Murine J774 macrophages were kindly provided by Dr. Juan Ugalde (IIBio, CONICET-UNSAM, Argentina). J774 and Hepa129 were maintained in RPMI, and HuH7, Hep3B, PM299L, and NM53 G03 cells in DMEM (Sigma-Aldrich, St. Louis, MO, USA), each supplemented with 10% heat-inactivated fetal bovine serum (FBS), 2 mM L-glutamine, 100 U/mL penicillin, and 100 μg/mL streptomycin. Cultures were incubated at 37°C in a humidified atmosphere containing 5% CO_2. Mycoplasma contamination was routinely tested using the e-Myco kit (Boca Scientific).

Primary hepatocytes were isolated by portal vein perfusion with collagenase (Sigma-Aldrich, St. Louis, MO, USA), followed by a 30 min decantation and two centrifugation steps (50 × g for 10 min). Viable cells were seeded in 6-well plates at a density of 2.5×10^5 cells per well in phenol red-free DMEM supplemented with 10% FBS, 2 mM glutamine, 100 μg/mL streptomycin, and 100 μg/mL penicillin. After 3 h, the medium was replaced with DMEM/F12 (70% DMEM + 30% F12) containing 10% FBS.

For viability assays, cells were seeded in 96-well plates at a density of 1,500–2,000 cells per well. Twenty-four hours later, cells were treated with increasing concentrations of the SMYD2 inhibitors AZ-505 and LLY-507, and standard MTT assays (Sigma-Aldrich, St. Louis, MO, USA) were performed after 3 days. Absorbance at 570 nm was measured on a Thermo Multiskan FC plate reader. Results were normalized to vehicle-treated controls (defined as 100% viability), and dose-response curves were generated by nonlinear regression analysis using GraphPad Prism software to calculate IC_{50} values.

In vivo HCC models

All procedures were approved by the university’s Animal Studies Committee and conducted in compliance with institutional and national regulations for animal research and welfare. Orthotopic tumors were generated by subcapsular injection of 1.25×10^5 Hepa129 cells or 5×10^4 PM299L cells into the left liver lobe of C3H/HeJ and C57BL/6J mice, respectively, via laparotomy. In the Hepa129 model, liver fibrosis was previously induced by thioacetamide administration.[101–103] Animals received AZ-505 or vehicle by i.p. injection. For the combination treatment, anti-PD-1 antibody was co-administered with the first and fourth AZ-505 doses. Tumor burden, ascites, and liver tissues were analyzed after six doses of AZ-505. Tumor dissection, size measurement and weighing were performed in a blinded manner to minimize potential bias. Details are described in the supplemental information and materials and methods section.

RNA extraction and RT-qPCR

Total RNA was extracted from J774, PM299L, and NM53 G03 cell lines and Hepa129 tumors using TRIzol reagent (Sigma-Aldrich, St. Louis, MO, USA). cDNA was synthesized with SuperScript II Reverse Transcriptase (Invitrogen, Carlsbad, CA, USA). Quantitative PCR (qPCR) reactions were performed using SYBR® Green (Invitrogen, Carlsbad, CA, USA), and primer sequences are listed in Table S1. Relative gene expression was calculated using the ΔΔCt method.[104]

Macrophage stimulation with tumor-derived CM

PM299L and NM53 G03 tumor cells were cultured in DMEM with 10% FBS and treated overnight with AZ-505 (5 μM) or vehicle (DMSO). After washing, cells were incubated for 24 h in serum-free medium, and the resulting conditioned media (CM) were collected. J774 macrophages were then exposed for 20 h to a 1:4 dilution of CM derived from PM299L or NM53 G03 cells (±AZ-505). Where indicated, macrophages were co-treated with the Wnt pathway modulators CHIR99021 (10 μM) or DKK1 (200 ng/mL) during CM stimulation. Control macrophages received fresh medium. Gene expression was assessed by RT-qPCR.

Flow cytometry and degranulation assays

Splenocytes were obtained by mechanical dissociation of spleens from tumor-bearing mice and either analyzed immediately or

re-stimulated *ex vivo* for degranulation assays. Tumors were enzymatically dissociated with collagenase and processed in parallel.

For surface marker analysis, splenocytes were stained with antibodies against MHCII (FITC; 553623), CD11c (PE; 553802), and CD80 (APC; 558703) for the identification of antigen-presenting cells, or with CD8α (PE-Cy5; 553034) and CD4 (PE; 553650) for T lymphocytes. For tumor-infiltrating lymphocytes, the same CD8α/CD4 staining strategy was applied. Tumor-associated macrophages were defined as $MHCII^+$ (PE; 107606) $F4/80^+$ (FITC; ab105155).

For degranulation assays, 1×10^7 splenocytes from each mouse were co-cultured with 5×10^5 PM299L cells in 6-well plates (RPMI + 10% FBS) for 4 days. On day 4, 1×10^6 splenocytes were re-stimulated with 1×10^5 PM299L cells in 24-well plates for 24 h. On day 5, cells were harvested and stained for surface CD8α (Alexa Fluor 488; 557668) and CD107a (APC; 121614). Staining was performed by incubating cells with the appropriate antibody combinations for 25 min on ice in the dark, followed by two washes with FACS buffer. Samples were acquired on a BD Accuri™ C6 Plus flow cytometer and analyzed using BD Accuri™ C6 software.

RNA sequencing

HuH7 cells (2×10^6) were seeded in p150 dishes and treated for 24 h with either the SMYD2 inhibitor LLY-507 (10 μM) or DMSO vehicle. For *in vivo* RNA-seq, three to four PM299L tumors per group were collected from two independent experiments, one comparing vehicle with AZ-505 and the other comparing vehicle with AZ-505 plus anti-PD-1. Total RNA was extracted from cell samples using the RNeasy Plus Mini Kit (Qiagen), which includes a genomic DNA elimination step, and from tumor samples using TRIzol reagent followed by ethanol/sodium acetate precipitation. RNA integrity was assessed using the Agilent 2100 Bioanalyzer, and only samples with RIN ≥8 were processed. RNA concentrations were measured using a Qubit fluorometer (Invitrogen). Libraries were prepared using the TruSeq Stranded Total RNA LT Sample Prep Kit (Illumina) and sequenced on an Illumina NextSeq 500 platform (McDermott Sequencing Core, UT Southwestern, for *in vitro* samples) and an Illumina NextSeq 2000 platform (Genomics Unit, CIMA, Universidad de Navarra, for *in vivo* samples).

Transcript alignment for cell samples was performed with TopHat and counts per million mapped reads (CPM) and differential expression analysis were conducted with the *edgeR* package (Bioconductor). For tumor samples, transcript quantification was performed with *kallisto* using the Mus musculus GRCm39 reference transcriptome, and differential expression analysis was conducted with DESeq2.

All RNA-seq datasets will be deposited in the Gene Expression Omnibus (GEO).

Statistical analysis

Statistical analysis was performed using 'R' software version 4.4.2 and PRISM V.8.0 software. Depending on data distribution, parametric or non-parametric tests were applied, and statistical significance was defined as $p < 0.05$ or FDR <0.05.

DATA AND CODE AVAILABILITY

The RNA-seq datasets generated in this study will be made available upon reasonable request and will be deposited in a public repository (e.g., GEO) upon publication.

ACKNOWLEDGMENTS

We thank Paula Roselló, Tobias Giovannetti, Anabel Cañete, Franco Puebla, Santiago Cabrera, and Guillermo Gastón for their professional technical assistance, and Norma Montalbetti for administrative support. This research was funded by grants from Agencia Nacional de Prmoción Científica y Tecnológica: PICT-2021-I-A-00975 (GM, JB), PICT-2018-1036 (JB) and PICT-2021- CAT-II-0012 (GM, JB).

AUTHOR CONTRIBUTIONS

B.B. performed experiments, analyzed data, and wrote the manuscript; M.F., E.F., and M.M. assisted with *in vivo* experiments, whereas M.J.C., M.C., B.M.R., F.M., and C.A. contributed to *in vitro* experiments; E.S. and J.A. contributed to the development and characterization of the NM53 G03 model; L.L. helped with the transcriptomic analysis; A.C. and J.A. provided support in manuscript writing; G.M. and J.B. conceptualized and guided the study, analyzed data, wrote the manuscript, and obtained funding. All authors revised and approved the manuscript.

DECLARATION OF INTERESTS

B.B. is an employee of HZ4 Liver Inc./Spectrum, 33130 Miami, FL, USA.

SUPPLEMENTAL INFORMATION

Supplemental information can be found online at https://doi.org/10.1016/j.omton.2026.201239.

REFERENCES

1. Bray, F., Laversanne, M., Sung, H., Ferlay, J., Siegel, R.L., Soerjomataram, I., and Jemal, A. (2024). Global cancer statistics 2022: GLOBOCAN estimates of incidence and mortality worldwide for 36 cancers in 185 countries. CA Cancer J. Clin. *74*, 229–263. https://doi.org/10.3322/caac.21834.
2. Sangro, B., Argemi, J., Ronot, M., Paradis, V., Meyer, T., Mazzaferro, V., Jepsen, P., Golfieri, R., Galle, P., Dawson, L., and Reig, M. (2025). EASL Clinical Practice Guidelines on the management of hepatocellular carcinoma. J. Hepatol. *82*, 315–374. https://doi.org/10.1016/j.jhep.2024.08.028.
3. Llovet, J.M., Kelley, R.K., Villanueva, A., Singal, A.G., Pikarsky, E., Roayaie, S., Lencioni, R., Koike, K., Zucman-Rossi, J., and Finn, R.S. (2021). Hepatocellular carcinoma. Nat. Rev. Dis. Primers *7*, 6. https://doi.org/10.1038/s41572-020-00240-3.
4. Yau, T., Galle, P.R., Decaens, T., Sangro, B., Qin, S., da Fonseca, L.G., Karachiwala, H., Blanc, J.-F., Park, J.-W., Gane, E., et al. (2025). Nivolumab plus ipilimumab versus lenvatinib or sorafenib as first-line treatment for unresectable hepatocellular carcinoma (CheckMate 9DW): an open-label, randomised, phase 3 trial. Lancet *405*, 1851–1864. https://doi.org/10.1016/S0140-6736(25)00403-9.
5. Fizazi, K., Maillard, A., Penel, N., Baciarello, G., Allouache, D., Daugaard, G., Van de Wouw, A., Soler, G., Vauleon, E., Chaigneau, L., et al. (2019). A phase III trial of empiric chemotherapy with cisplatin and gemcitabine or systemic treatment tailored by molecular gene expression analysis in patients with carcinomas of an unknown primary (CUP) site (GEFCAPI 04). Ann. Oncol. *30*, v851. https://doi.org/10.1093/annonc/mdz394.
6. Finn, R.S., Ryoo, B.-Y., Merle, P., Kudo, M., Bouattour, M., Lim, H.Y., Breder, V., Edeline, J., Chao, Y., Ogasawara, S., et al. (2020). Pembrolizumab As Second-Line Therapy in Patients With Advanced Hepatocellular Carcinoma in KEYNOTE-240: A Randomized, Double-Blind, Phase III Trial. J. Clin. Oncol. *38*, 193–202. https://doi.org/10.1200/JCO.19.01307.
7. Sangro, B., Gomez-Martin, C., de la Mata, M., Iñarrairaegui, M., Garralda, E., Barrera, P., Riezu-Boj, J.I., Larrea, E., Alfaro, C., Sarobe, P., et al. (2013). A clinical trial of CTLA-4 blockade with tremelimumab in patients with hepatocellular carcinoma and chronic hepatitis C. J. Hepatol. *59*, 81–88. https://doi.org/10.1016/j.jhep.2013.02.022.

8. Duffy, A.G., Ulahannan, S.V., Makorova-Rusher, O., Rahma, O., Wedemeyer, H., Pratt, D., Davis, J.L., Hughes, M.S., Heller, T., ElGindi, M., et al. (2017). Tremelimumab in combination with ablation in patients with advanced hepatocellular carcinoma. J. Hepatol. *66*, 545–551. https://doi.org/10.1016/j.jhep.2016.10.029.

9. Finn, R.S., Qin, S., Ikeda, M., Galle, P.R., Ducreux, M., Kim, T.-Y., Kudo, M., Breder, V., Merle, P., Kaseb, A.O., et al. (2020). Atezolizumab plus Bevacizumab in Unresectable Hepatocellular Carcinoma. N. Engl. J. Med. Overseas. Ed. *382*, 1894–1905. https://doi.org/10.1056/NEJMoa1915745.

10. Xie, Q., Zhang, P., Wang, Y., Mei, W., and Zeng, C. (2022). Overcoming resistance to immune checkpoint inhibitors in hepatocellular carcinoma: Challenges and opportunities. Front. Oncol. *12*, 958720. https://doi.org/10.3389/fonc.2022.958720.

11. Rizzo, A., and Brandi, G. (2021). Biochemical predictors of response to immune checkpoint inhibitors in unresectable hepatocellular carcinoma. Cancer Treat. Res. Commun. *27*, 100328. https://doi.org/10.1016/j.ctarc.2021.100328.

12. Liang, Z., Li, S., Wang, Z., Zhou, J., Huang, Z., Li, J., Bao, H., Yam, J.W.P., and Xu, Y. (2025). Unraveling the Role of the Wnt Pathway in Hepatocellular Carcinoma: From Molecular Mechanisms to Therapeutic Implications. J. Clin. Transl. Hepatol. *13*, 315. https://doi.org/10.14218/JCTH.2024.00401.

13. Ma, S., Meng, G., Liu, T., You, J., He, R., Zhao, X., and Cui, Y. (2024). The Wnt signaling pathway in hepatocellular carcinoma: Regulatory mechanisms and therapeutic prospects. Biomed. Pharmacother. *180*, 117508. https://doi.org/10.1016/j.biopha.2024.117508.

14. Harding, J.J., Nandakumar, S., Armenia, J., Khalil, D.N., Albano, M., Ly, M., Shia, J., Hechtman, J.F., Kundra, R., El Dika, I., et al. (2019). Prospective Genotyping of Hepatocellular Carcinoma: Clinical Implications of Next-Generation Sequencing for Matching Patients to Targeted and Immune Therapies. Clin. Cancer Res. *25*, 2116–2126. https://doi.org/10.1158/1078-0432.CCR-18-2293.

15. Ruiz de Galarreta, M., Bresnahan, E., Molina-Sánchez, P., Lindblad, K.E., Maier, B., Sia, D., Puigvehi, M., Miguela, V., Casanova-Acebes, M., Dhainaut, M., et al. (2019). β-Catenin Activation Promotes Immune Escape and Resistance to Anti–PD-1 Therapy in Hepatocellular Carcinoma. Cancer Discov. *9*, 1124–1141. https://doi.org/10.1158/2159-8290.CD-19-0074.

16. Pinyol, R., Sia, D., and Llovet, J.M. (2019). Immune Exclusion-Wnt/CTNNB1 Class Predicts Resistance to Immunotherapies in HCC. Clin. Cancer Res. *25*, 2021–2023. https://doi.org/10.1158/1078-0432.CCR-18-3778.

17. De Lorenzo, S., Tovoli, F., and Trevisani, F. (2022). Mechanisms of Primary and Acquired Resistance to Immune Checkpoint Inhibitors in Patients with Hepatocellular Carcinoma. Cancers (Basel) *14*, 4616. https://doi.org/10.3390/cancers14194616.

18. Shrestha, R., Bridle, K.R., Crawford, D.H.G., and Jayachandran, A. (2021). Immune checkpoint molecules are regulated by transforming growth factor (TGF)- *β* 1-induced epithelial-to-mesenchymal transition in hepatocellular carcinoma. Int. J. Med. Sci. *18*, 2466–2479. https://doi.org/10.7150/ijms.54239.

19. Li, X., Xiang, Y., Li, F., Yin, C., Li, B., and Ke, X. (2019). WNT/β-Catenin Signaling Pathway Regulating T Cell-Inflammation in the Tumor Microenvironment. Front. Immunol. *10*, 2293. https://doi.org/10.3389/fimmu.2019.02293.

20. Abu-Farha, M., Lambert, J.-P., Al-Madhoun, A.S., Elisma, F., Skerjanc, I.S., and Figeys, D. (2008). The Tale of Two Domains. Mol. Cell. Proteomics *7*, 560–572. https://doi.org/10.1074/mcp.M700271-MCP200.

21. Yi, X., Jiang, X.-J., and Fang, Z.-M. (2019). Histone methyltransferase SMYD2: ubiquitous regulator of disease. Clin. Epigenetics *11*, 112. https://doi.org/10.1186/s13148-019-0711-4.

22. Xia, K., Zhou, Y., Xie, Y., and Cai, Y. (2025). Role of SMYD2 in gastrointestinal cancer progression (Review). Oncol. Lett. *29*, 1–12. https://doi.org/10.3892/ol.2025.15028.

23. Han, T.-S., Kim, D.-S., Son, M.-Y., and Cho, H.-S. (2024). SMYD family in cancer: epigenetic regulation and molecular mechanisms of cancer proliferation, metastasis, and drug resistance. Exp. Mol. Med. *56*, 2325–2336. https://doi.org/10.1038/s12276-024-01326-8.

24. Zucman-Rossi, J., Villanueva, A., Nault, J.-C., and Llovet, J.M. (2015). Genetic Landscape and Biomarkers of Hepatocellular Carcinoma. Gastroenterology *149*, 1226–1239.e4. https://doi.org/10.1053/j.gastro.2015.05.061.

25. Komatsu, S., Imoto, I., Tsuda, H., Kozaki, K.i., Muramatsu, T., Shimada, Y., Aiko, S., Yoshizumi, Y., Ichikawa, D., Otsuji, E., and Inazawa, J. (2009). Overexpression of SMYD2 relates to tumor cell proliferation and malignant outcome of esophageal squamous cell carcinoma. Carcinogenesis *30*, 1139–1146. https://doi.org/10.1093/carcin/bgp116.

26. Komatsu, S., Ichikawa, D., Hirajima, S., Nagata, H., Nishimura, Y., Kawaguchi, T., Miyamae, M., Okajima, W., Ohashi, T., Konishi, H., et al. (2015). Overexpression of SMYD2 contributes to malignant outcome in gastric cancer. Br. J. Cancer *112*, 357–364. https://doi.org/10.1038/bjc.2014.543.

27. Zeng, Y., Qiu, R., Yang, Y., Gao, T., Zheng, Y., Huang, W., Gao, J., Zhang, K., Liu, R., Wang, S., et al. (2019). Regulation of EZH2 by SMYD2-Mediated Lysine Methylation Is Implicated in Tumorigenesis. Cell Rep. *29*, 1482–1498.e4. https://doi.org/10.1016/j.celrep.2019.10.004.

28. Brown, M.A., Sims, R.J., Gottlieb, P.D., and Tucker, P.W. (2006). Identification and characterization of Smyd2: a split SET/MYND domain-containing histone H3 lysine 36-specific methyltransferase that interacts with the Sin3 histone deacetylase complex. Mol. Cancer *5*, 26. https://doi.org/10.1186/1476-4598-5-26.

29. Huang, J., Perez-Burgos, L., Placek, B.J., Sengupta, R., Richter, M., Dorsey, J.A., Kubicek, S., Opravil, S., Jenuwein, T., and Berger, S.L. (2006). Repression of p53 activity by Smyd2-mediated methylation. Nature *444*, 629–632. https://doi.org/10.1038/nature05287.

30. Wang, F., Malnassy, G., and Qiu, W. (2021). The Epigenetic Regulation of Microenvironment in Hepatocellular Carcinoma. Front. Oncol. *11*, 653037. https://doi.org/10.3389/fonc.2021.653037.

31. Yang, J., Xu, J., Wang, W., Zhang, B., Yu, X., and Shi, S. (2023). Epigenetic regulation in the tumor microenvironment: molecular mechanisms and therapeutic targets. Signal Transduct. Targeted Ther. *8*, 210. https://doi.org/10.1038/s41392-023-01480-x.

32. Ding, K., Xie, R., Han, B., Zheng, H., and Tian, T. (2025). Histone methyltransferase SMYD2 regulates the activation of hepatic stellate cells by activating TLR4 signaling. Sci. Rep. *15*, 13166. https://doi.org/10.1038/s41598-025-96699-9.

33. Wang, S., Zhao, C., Li, D., Liu, Q., Mao, C., Ding, S., Zhang, S., and Shang, W. (2025). Identification of SMYD2 as a candidate diagnostic and prognostic biomarker for gastric cancer. Front. Oncol. *15*, 1617971. https://doi.org/10.3389/fonc.2025.1617971.

34. Yadav, A.K., and Singh, T.R. (2023). Computational approach for assessing the involvement of SMYD2 protein in human cancers using TCGA data. J. Genet. Eng. Biotechnol. *21*, 122. https://doi.org/10.1186/s43141-023-00594-7.

35. Park, S., Cho, J.H., Kim, J.-H., and Kim, J.-A. (2024). Histone lysine methylation modifiers controlled by protein stability. Exp. Mol. Med. *56*, 2127–2144. https://doi.org/10.1038/s12276-024-01329-5.

36. Jiao, L., Liu, Y., Yu, X.-Y., Pan, X., Zhang, Y., Tu, J., Song, Y.-H., and Li, Y. (2023). Ribosome biogenesis in disease: new players and therapeutic targets. Signal Transduct. Targeted Ther. *8*, 15. https://doi.org/10.1038/s41392-022-01285-4.

37. Elhamamsy, A.R., Metge, B.J., Alsheikh, H.A., Shevde, L.A., and Samant, R.S. (2022). Ribosome Biogenesis: A Central Player in Cancer Metastasis and Therapeutic Resistance. Cancer Res. *82*, 2344–2353. https://doi.org/10.1158/0008-5472.CAN-21-4087.

38. Rui, L. (2014). Energy Metabolism in the Liver. In Comprehensive Physiology, pp. 177–197. https://doi.org/10.1002/cphy.c130024.

39. Li, L.X., Zhou, J.X., Calvet, J.P., Godwin, A.K., Jensen, R.A., and Li, X. (2018). Lysine methyltransferase SMYD2 promotes triple negative breast cancer progression. Cell Death Dis. *9*, 326. https://doi.org/10.1038/s41419-018-0347-x.

40. Ferguson, A.D., Larsen, N.A., Howard, T., Pollard, H., Green, I., Grande, C., Cheung, T., Garcia-Arenas, R., Cowen, S., Wu, J., et al. (2011). Structural Basis of Substrate Methylation and Inhibition of SMYD2. Structure *19*, 1262–1273. https://doi.org/10.1016/j.str.2011.06.011.

41. Xu, T., Cui, B., Liu, F., Liu, M., Hou, X., Hong, X., and Qi, H. (2025). A Novel Inhibitor of Methyltransferase SMYD2 , AZ505 Protects Against Peritoneal Fibrosis in Mice. Clin. Exp. Pharmacol. Physiol. *52*, e70035. https://doi.org/10.1111/1440-1681.70035.

42. Kwantwi, L.B. (2023). Overcoming anti-PD-1/PD-L1 immune checkpoint blockade resistance: the role of macrophage, neutrophils and mast cells in the

tumor microenvironment. Clin. Exp. Med. *23*, 3077–3091. https://doi.org/10.1007/s10238-023-01059-4.

43. Zhou, D., Luan, J., Huang, C., and Li, J. (2021). Tumor-Associated Macrophages in Hepatocellular Carcinoma: Friend or Foe? Gut Liver *15*, 500–516. https://doi.org/10.5009/gnl20223.

44. Zheng, H., Peng, X., Yang, S., Li, X., Huang, M., Wei, S., Zhang, S., He, G., Liu, J., Fan, Q., et al. (2023). Targeting tumor-associated macrophages in hepatocellular carcinoma: biology, strategy, and immunotherapy. Cell Death Discov. *9*, 65. https://doi.org/10.1038/s41420-023-01356-7.

45. Martynova, E., Rizvanov, A., Urbanowicz, R.A., and Khaiboullina, S. (2022). Inflammasome Contribution to the Activation of Th1, Th2, and Th17 Immune Responses. Front. Microbiol. *13*, 851835. https://doi.org/10.3389/fmicb.2022.851835.

46. Watford, W.T., Moriguchi, M., Morinobu, A., and O'Shea, J.J. (2003). The biology of IL-12: coordinating innate and adaptive immune responses. Cytokine Growth Factor Rev. *14*, 361–368. https://doi.org/10.1016/S1359-6101(03)00043-1.

47. Wang, H., Ruan, G., Li, Y., and Liu, X. (2025). The Role and Potential Application of IL-12 in the Immune Regulation of Tuberculosis. Int. J. Mol. Sci. *26*, 3106. https://doi.org/10.3390/ijms26073106.

48. Sia, D., Jiao, Y., Martinez-Quetglas, I., Kuchuk, O., Villacorta-Martin, C., Castro de Moura, M., Putra, J., Camprecios, G., Bassaganyas, L., Akers, N., et al. (2017). Identification of an Immune-specific Class of Hepatocellular Carcinoma, Based on Molecular Features. Gastroenterology *153*, 812–826. https://doi.org/10.1053/j.gastro.2017.06.007.

49. Silva, L., Egea, J., Villanueva, L., Ruiz, M., Llopiz, D., Repáraz, D., Aparicio, B., Lasarte-Cia, A., Lasarte, J.J., Ruiz de Galarreta, M., et al. (2020). Cold-Inducible RNA Binding Protein as a Vaccination Platform to Enhance Immunotherapeutic Responses against Hepatocellular Carcinoma. Cancers (Basel) *12*, 3397. https://doi.org/10.3390/cancers12113397.

50. Chen, X., and Calvisi, D.F. (2014). Hydrodynamic Transfection for Generation of Novel Mouse Models for Liver Cancer Research. Am. J. Pathol. *184*, 912–923. https://doi.org/10.1016/j.ajpath.2013.12.002.

51. MOON, H., PARK, H., and RO, S.W. (2021). c-Myc-driven Hepatocarcinogenesis. Anticancer Res. *41*, 4937–4946. https://doi.org/10.21873/anticanres.15307.

52. Feng, H., Zhuo, Y., Zhang, X., Li, Y., Li, Y., Duan, X., Shi, J., Xu, C., Gao, Y., and Yu, Z. (2022). Tumor Microenvironment in Hepatocellular Carcinoma: Key Players for Immunotherapy. J. Hepatocell. Carcinoma *9*, 1109–1125. https://doi.org/10.2147/JHC.S381764.

53. Liu, S., Jia, M., and Dai, R. (2023). Deciphering the tumour immune microenvironment of hepatocellular carcinoma. Scand. J. Immunol. *98*, e13327. https://doi.org/10.1111/sji.13327.

54. Alok, A., Lei, Z., Jagannathan, N.S., Kaur, S., Harmston, N., Rozen, S.G., Tucker-Kellogg, L., and Virshup, D.M. (2017). Wnt proteins synergize to activate β-catenin signaling. J. Cell Sci. *130*, 1532–1544. https://doi.org/10.1242/jcs.198093.

55. Arensman, M.D., Kovochich, A.N., Kulikauskas, R.M., Lay, A.R., Yang, P.-T., Li, X., Donahue, T., Major, M.B., Moon, R.T., Chien, A.J., and Dawson, D.W. (2014). WNT7B mediates autocrine Wnt/β-catenin signaling and anchorage-independent growth in pancreatic adenocarcinoma. Oncogene *33*, 899–908. https://doi.org/10.1038/onc.2013.23.

56. Akiri, G., Cherian, M.M., Vijayakumar, S., Liu, G., Bafico, A., and Aaronson, S.A. (2009). Wnt pathway aberrations including autocrine Wnt activation occur at high frequency in human non-small-cell lung carcinoma. Oncogene *28*, 2163–2172. https://doi.org/10.1038/onc.2009.82.

57. Benhaj, K., Akcali, K., and Ozturk, M. (2006). Redundant expression of canonical Wnt ligands in human breast cancer cell lines. Oncol. Rep. *15*, 701–707. https://doi.org/10.3892/or.15.3.701.

58. Pacella, I., Cammarata, I., Focaccetti, C., Miacci, S., Gulino, A., Tripodo, C., Ravà, M., Barnaba, V., and Piconese, S. (2018). Wnt3a Neutralization Enhances T-cell Responses through Indirect Mechanisms and Restrains Tumor Growth. Cancer Immunol. Res. *6*, 953–964. https://doi.org/10.1158/2326-6066.CIR-17-0713.

59. Feng, Y., Liang, Y., Zhu, X., Wang, M., Gui, Y., Lu, Q., Gu, M., Xue, X., Sun, X., He, W., et al. (2018). The signaling protein Wnt5a promotes TGFβ1-mediated macrophage polarization and kidney fibrosis by inducing the transcriptional regulators Yap/Taz. J. Biol. Chem. *293*, 19290–19302. https://doi.org/10.1074/jbc.RA118.005457.

60. Liu, Q., Yang, C., Wang, S., Shi, D., Wei, C., Song, J., Lin, X., Dou, R., Bai, J., Xiang, Z., et al. (2020). Wnt5a-induced M2 polarization of tumor-associated macrophages via IL-10 promotes colorectal cancer progression. Cell Commun. Signal. *18*, 51. https://doi.org/10.1186/s12964-020-00557-2.

61. Cantero, M.J., Bueloni, B., Gonzalez Llamazares, L., Fiore, E., Lameroli, L., Atorrasagasti, C., Mazzolini, G., Malvicini, M., Bayo, J., and García, M.G. (2024). Modified mesenchymal stromal cells by in vitro transcribed mRNA: a therapeutic strategy for hepatocellular carcinoma. Stem Cell Res. Ther. *15*, 208. https://doi.org/10.1186/s13287-024-03806-0.

62. Sangro, B., Melero, I., Wadhawan, S., Finn, R.S., Abou-Alfa, G.K., Cheng, A.-L., Yau, T., Furuse, J., Park, J.-W., Boyd, Z., et al. (2020). Association of inflammatory biomarkers with clinical outcomes in nivolumab-treated patients with advanced hepatocellular carcinoma. J. Hepatol. *73*, 1460–1469. https://doi.org/10.1016/j.jhep.2020.07.026.

63. Charoentong, P., Finotello, F., Angelova, M., Mayer, C., Efremova, M., Rieder, D., Hackl, H., and Trajanoski, Z. (2017). Pan-cancer Immunogenomic Analyses Reveal Genotype-Immunophenotype Relationships and Predictors of Response to Checkpoint Blockade. Cell Rep. *18*, 248–262. https://doi.org/10.1016/j.celrep.2016.12.019.

64. Akbulut, Z., Aru, B., Aydın, F., and Yanıkkaya Demirel, G. (2024). Immune checkpoint inhibitors in the treatment of hepatocellular carcinoma. Front. Immunol. *15*. https://doi.org/10.3389/fimmu.2024.1379622.

65. Carloni, R., Sabbioni, S., Rizzo, A., Ricci, A.D., Palloni, A., Petrarota, C., Cusmai, A., Tavolari, S., Gadaleta-Caldarola, G., and Brandi, G. (2023). Immune-Based Combination Therapies for Advanced Hepatocellular Carcinoma. J. Hepatocell. Carcinoma *10*, 1445–1463. https://doi.org/10.2147/JHC.S390963.

66. Yu, J., Li, M., Ren, B., Cheng, L., Wang, X., Ma, Z., Yong, W.P., Chen, X., Wang, L., and Goh, B.C. (2023). Unleashing the efficacy of immune checkpoint inhibitors for advanced hepatocellular carcinoma: factors, strategies, and ongoing trials. Front. Pharmacol. *14*, 1261575. https://doi.org/10.3389/fphar.2023.1261575.

67. Kwee, S.A., and Tiirikainen, M. (2021). Beta-catenin activation and immunotherapy resistance in hepatocellular carcinoma: mechanisms and biomarkers. Hepatoma Res. *7*, 8. https://doi.org/10.20517/2394-5079.2020.124.

68. Hamamoto, R., Saloura, V., and Nakamura, Y. (2015). Critical roles of non-histone protein lysine methylation in human tumorigenesis. Nat. Rev. Cancer *15*, 110–124. https://doi.org/10.1038/nrc3884.

69. Nakakido, M., Deng, Z., Suzuki, T., Dohmae, N., Nakamura, Y., and Hamamoto, R. (2015). Dysregulation of AKT Pathway by SMYD2-Mediated Lysine Methylation on PTEN. Neoplasia *17*, 367–373. https://doi.org/10.1016/j.neo.2015.03.002.

70. Saddic, L.A., West, L.E., Aslanian, A., Yates, J.R., Rubin, S.M., Gozani, O., and Sage, J. (2010). Methylation of the Retinoblastoma Tumor Suppressor by SMYD2. J. Biol. Chem. *285*, 37733–37740. https://doi.org/10.1074/jbc.M110.137612.

71. Cho, H.-S., Hayami, S., Toyokawa, G., Maejima, K., Yamane, Y., Suzuki, T., Dohmae, N., Kogure, M., Kang, D., Neal, D.E., et al. (2012). RB1 Methylation by SMYD2 Enhances Cell Cycle Progression through an Increase of RB1 Phosphorylation. Neoplasia *14*, 476. https://doi.org/10.1593/neo.12656.

72. Wang, Z., Zhu, S., Jia, Y., Wang, Y., Kubota, N., Fujiwara, N., Gordillo, R., Lewis, C., Zhu, M., Sharma, T., et al. (2023). Positive selection of somatically mutated clones identifies adaptive pathways in metabolic liver disease. Cell *186*, 1968–1984.e20. https://doi.org/10.1016/j.cell.2023.03.014.

73. Gomaa, A.I., Al-Khatib, A., Abdel-Razek, W., Hashim, M.S., and Waked, I. (2015). Ascites and alpha-fetoprotein improve prognostic performance of Barcelona Clinic Liver Cancer staging. World J. Gastroenterol. *21*, 5654–5662. https://doi.org/10.3748/wjg.v21.i18.5654.

74. Müller, L., Bender, D., Gairing, S.J., Foerster, F., Weinmann, A., Mittler, J., Stoehr, F., Halfmann, M.C., Mähringer-Kunz, A., Galle, P.R., et al. (2024). Amount of ascites impacts survival in patients with hepatocellular carcinoma undergoing transarterial chemoembolization advocating for volumetric assessment. Sci. Rep. *14*, 16550. https://doi.org/10.1038/s41598-024-67312-2.

75. Liao, J.-I., Ho, S.-Y., Liu, P.-H., Hsu, C.-Y., Huang, Y.-H., Su, C.-W., Hou, M.-C., and Huo, T.-I. (2023). Prognostic Prediction for Patients with Hepatocellular

Carcinoma and Ascites: Role of Albumin-Bilirubin (ALBI) Grade and Easy (EZ)-ALBI Grade. Cancers (Basel) *15*, 753. https://doi.org/10.3390/cancers15030753.

76. Martin-Orozco, E., Sanchez-Fernandez, A., Ortiz-Parra, I., and Ayala-San Nicolas, M. (2019). WNT Signaling in Tumors: The Way to Evade Drugs and Immunity. Front. Immunol. *10*, 2854. https://doi.org/10.3389/fimmu.2019.02854.
77. Yuan, Y., Wu, D., Hou, Y., Zhang, Y., Tan, C., Nie, X., Zhao, Z., and Hou, J. (2024). Wnt signaling: Modulating tumor-associated macrophages and related immunotherapeutic insights. Biochem. Pharmacol. *223*, 116154. https://doi.org/10.1016/j.bcp.2024.116154.
78. Yang, Y., Ye, Y.-C., Chen, Y., Zhao, J.-L., Gao, C.-C., Han, H., Liu, W.-C., and Qin, H.-Y. (2018). Crosstalk between hepatic tumor cells and macrophages via Wnt/β-catenin signaling promotes M2-like macrophage polarization and reinforces tumor malignant behaviors. Cell Death Dis. *9*, 793. https://doi.org/10.1038/s41419-018-0818-0.
79. de Pellegars-Malhortie, A., Picque Lasorsa, L., Mazard, T., Granier, F., and Prévostel, C. (2024). Why Is Wnt/β-Catenin Not Yet Targeted in Routine Cancer Care? Pharmaceuticals *17*, 949. https://doi.org/10.3390/ph17070949.
80. Liu, J., Xiao, Q., Xiao, J., Niu, C., Li, Y., Zhang, X., Zhou, Z., Shu, G., and Yin, G. (2022). Wnt/β-catenin signalling: function, biological mechanisms, and therapeutic opportunities. Signal Transduct. Targeted Ther. *7*, 3. https://doi.org/10.1038/s41392-021-00762-6.
81. Muto, S., Enta, A., Maruya, Y., Inomata, S., Yamaguchi, H., Mine, H., Takagi, H., Ozaki, Y., Watanabe, M., Inoue, T., et al. (2023). Wnt/β-Catenin Signaling and Resistance to Immune Checkpoint Inhibitors: From Non-Small-Cell Lung Cancer to Other Cancers. Biomedicines *11*, 190. https://doi.org/10.3390/biomedicines11010190.
82. Dantzer, C., Dif, L., Vaché, J., Basbous, S., Billottet, C., and Moreau, V. (2024). Specific features of ß-catenin-mutated hepatocellular carcinomas. Br. J. Cancer *131*, 1871–1880. https://doi.org/10.1038/s41416-024-02849-7.
83. Deng, X., Hamamoto, R., Vougiouklakis, T., Wang, R., Yoshioka, Y., Suzuki, T., Dohmae, N., Matsuo, Y., Park, J.-H., and Nakamura, Y. (2017). Critical roles of SMYD2-mediated β-catenin methylation for nuclear translocation and activation of Wnt signaling. Oncotarget *8*, 55837–55847. https://doi.org/10.18632/oncotarget.19646.
84. Meng, F., Liu, X., Lin, C., Xu, L., Liu, J., Zhang, P., Zhang, X., Song, J., Yan, Y., Ren, Z., et al. (2020). SMYD2 suppresses APC2 expression to activate the Wnt/β-catenin pathway and promotes epithelial-mesenchymal transition in colorectal cancer. Am. J. Cancer Res. *10*, 997–1011.
85. Biterge Süt, B. (2020). Computational analysis of TP53 vs. CTNNB1 mutations in hepatocellular carcinoma suggests distinct cancer subtypes with differential gene expression profiles and chromatin states. Comput. Biol. Chem. *89*, 107404. https://doi.org/10.1016/j.compbiolchem.2020.107404.
86. Ally, A., Balasundaram, M., Carlsen, R., Chuah, E., Clarke, A., Dhalla, N., Holt, R.A., Jones, S.J.M., Lee, D., Ma, Y., et al. (2017). Comprehensive and Integrative Genomic Characterization of Hepatocellular Carcinoma. Cell *169*, 1327–1341.e23. https://doi.org/10.1016/j.cell.2017.05.046.
87. Kancherla, V., Abdullazade, S., Matter, M.S., Lanzafame, M., Quagliata, L., Roma, G., Hoshida, Y., Terracciano, L.M., Ng, C.K.Y., and Piscuoglio, S. (2018). Genomic Analysis Revealed New Oncogenic Signatures in TP53-Mutant Hepatocellular Carcinoma. Front. Genet. *9*, 2. https://doi.org/10.3389/fgene.2018.00002.
88. Tang, M., Chen, G., Tu, B., Hu, Z., Huang, Y., DuFort, C.C., Wan, X., Mao, Z., Liu, Y., Zhu, W.-G., and Lu, W. (2023). SMYD2 inhibition–mediated hypomethylation of Ku70 contributes to impaired nonhomologous end joining repair and antitumor immunity. Sci. Adv. *9*, eade6624. https://doi.org/10.1126/sciadv.ade6624.
89. Xu, G., Liu, G., Xiong, S., Liu, H., Chen, X., and Zheng, B. (2015). The Histone Methyltransferase Smyd2 Is a Negative Regulator of Macrophage Activation by Suppressing Interleukin 6 (IL-6) and Tumor Necrosis Factor α (TNF-α) Production. J. Biol. Chem. *290*, 5414–5423. https://doi.org/10.1074/jbc.M114.610345.
90. Bergenfelz, C., Medrek, C., Ekström, E., Jirström, K., Janols, H., Wullt, M., Bredberg, A., and Leandersson, K. (2012). Wnt5a Induces a Tolerogenic Phenotype of Macrophages in Sepsis and Breast Cancer Patients. J. Immunol. *188*, 5448–5458. https://doi.org/10.4049/jimmunol.1103378.
91. Mehmeti, M., Bergenfelz, C., Källberg, E., Millrud, C.R., Björk, P., Ivars, F., Johansson-Lindbom, B., Kjellström, S., André, I., and Leandersson, K. (2019). Wnt5a is a TLR2/4-ligand that induces tolerance in human myeloid cells. Commun. Biol. *2*, 176. https://doi.org/10.1038/s42003-019-0432-4.
92. Zhao, F., Xiao, C., Evans, K.S., Theivanthiran, T., DeVito, N., Holtzhausen, A., Liu, J., Liu, X., Boczkowski, D., Nair, S., et al. (2018). Paracrine Wnt5a-β-Catenin Signaling Triggers a Metabolic Program that Drives Dendritic Cell Tolerization. Immunity *48*, 147–160.e7. https://doi.org/10.1016/j.immuni.2017.12.004.
93. Cheng, D., Chai, J., Wang, H., Fu, L., Peng, S., and Ni, X. (2021). Hepatic macrophages: Key players in the development and progression of liver fibrosis. Liver Int. *41*, 2279–2294. https://doi.org/10.1111/liv.14940.
94. Lee, J.J., Ng, K.Y., and Bakhtiar, A. (2025). Extracellular matrix: unlocking new avenues in cancer treatment. Biomark. Res. *13*, 78. https://doi.org/10.1186/s40364-025-00757-3.
95. Ghahremanifard, P., Chanda, A., Bonni, S., and Bose, P. (2020). TGF-β Mediated Immune Evasion in Cancer—Spotlight on Cancer-Associated Fibroblasts. Cancers (Basel) *12*, 3650. https://doi.org/10.3390/cancers12123650.
96. Roy, A.M., Iyer, R., and Chakraborty, S. (2023). The extracellular matrix in hepatocellular carcinoma: Mechanisms and therapeutic vulnerability. Cell Rep. Med. *4*, 101170. https://doi.org/10.1016/j.xcrm.2023.101170.
97. Manfredi, G.F., Celsa, C., John, C., Jones, C., Acuti, N., Scheiner, B., Fulgenzi, C.A.M., Korolewicz, J., Pinter, M., Gennari, A., et al. (2023). Mechanisms of Resistance to Immunotherapy in Hepatocellular Carcinoma. J. Hepatocell. Carcinoma *10*, 1955–1971. https://doi.org/10.2147/JHC.S291553.
98. Chen, J., Gingold, J.A., and Su, X. (2019). Immunomodulatory TGF-β Signaling in Hepatocellular Carcinoma. Trends Mol. Med. *25*, 1010–1023. https://doi.org/10.1016/j.molmed.2019.06.007.
99. Jiang, Z., Wu, Y., Miao, Y., Deng, K., Yang, F., Xu, S., Wang, Y., You, R., Zhang, L., Fan, Y., et al. (2024). HCCDB v2.0: Decompose Expression Variations by Single-cell RNA-seq and Spatial Transcriptomics in HCC. Genom. Proteom. Bioinform. *22*, qzae011. https://doi.org/10.1093/gpbjnl/qzae011.
100. FireBrowse Portal (Broad Institute TCGA GDAC)..
101. Rodríguez, M.M., Onorato, A., Cantero, M.J., Domínguez, L., Bayo, J., Fiore, E., García, M., Atorrasagasti, C., Canbay, A., Malvicini, M., and Mazzolini, G.D. (2021). 4-methylumbelliferone-mediated polarization of M1 macrophages correlate with decreased hepatocellular carcinoma aggressiveness in mice. Sci. Rep. *11*, 6310. https://doi.org/10.1038/s41598-021-85491-0.
102. Ciarlantini, M.S., Barquero, A., Bayo, J., Wetzler, D., Dodes Traian, M.M., Bucci, H.A., Fiore, E.J., Gandolfi Donadío, L., Defelipe, L., Turjanski, A., et al. (2021). Development of an Improved Guanidine-Based Rac1 Inhibitor with in vivo Activity against Non-Small Cell Lung Cancer. ChemMedChem *16*, 1011–1021. https://doi.org/10.1002/cmdc.202000763.
103. Bayo, J., Fiore, E.J., Dominguez, L.M., Cantero, M.J., Ciarlantini, M.S., Malvicini, M., Atorrasagasti, C., Garcia, M.G., Rossi, M., Cavasotto, C., et al. (2021). Bioinformatic analysis of RHO family of GTPases identifies RAC1 pharmacological inhibition as a new therapeutic strategy for hepatocellular carcinoma. Gut *70*, 1362–1374. https://doi.org/10.1136/gutjnl-2020-321454.
104. Sukowati, C.H.C., Anfuso, B., Fiore, E., Ie, S.I., Raseni, A., Vascotto, F., Avellini, C., Mazzolini, G., and Tiribelli, C. (2019). Hyaluronic acid inhibition by 4-methylumbelliferone reduces the expression of cancer stem cells markers during hepatocarcinogenesis. Sci. Rep. *9*, 4026. https://doi.org/10.1038/s41598-019-40436-6.

OMTON, Volume *34*

Supplemental information

Targeting SMYD2 improves immunotherapy response in experimental hepatocellular carcinoma

Bárbara Bueloni, Mariel Fusco, Esteban Fiore, Mariana Malvicini, María Jose Cantero, Lucía Lameroli, Mailín Casadei, Brian Martinez Ruiz, Florencia Mercogliano, Eva Santamaría, Catalina Atorrasagasti, Josepmaria Argemi, Ali Canbay, Juan Bayo, and Guillermo Mazzolini

Supplemental materials and methods.

Bioinformatic analyses

For the initial assessment of SMYD2 expression, RSEM-normalized RNA-seq data from 15 independent HCC datasets were obtained from HCCDB v2, including their adjacent non-tumoral tissues. Additional adjacent non-tumoral tissues from The Cancer Genome Atlas (TCGA) were retrieved via Firebrowse (http://firebrowse.org), excluding tissue types with fewer than three samples to ensure consistency. Subsequent analyses focused on TCGA (n = 377, RNA-seq), the International Cancer Genome Consortium (ICGC, n = 203, RNA-seq), and GSE14520 (n = 225, microarray), as these represent well-characterized and widely used HCC transcriptomic datasets. These cohorts comprise patients with primary tumors who underwent surgical resection. Analyses were restricted to patients with a single tumor sample, considering only protein-coding genes expressed in at least one patient. For tumor versus non-tumoral comparisons, a subset of paired samples was selected (TCGA, n = 50; ICGC, n = 174; GSE14520, n = 214).

Gene expression values from RNA-seq datasets were log-transformed as log2(RSEM + 1). Differentially expressed genes (DEGs) were identified using paired t-tests with a false discovery rate (FDR) threshold of < 0.05. Gene ontology (GO) and pathway analyses were done using ToppGene (ToppFun) on DEGs with log2 fold change > |0.378| and FDR < 0.05. Upstream regulator analysis was carried out with Ingenuity Pathway Analysis (IPA®, Qiagen), on DEGs from tumor RNA-seq data (log2 fold change > |0.585|, FDR < 0.05), using the IPA z-score algorithm to predict regulator activation or inhibition based on curated molecular interactions. Immunophenoscores (IPS) for TCGA-LIHC samples were obtained from The Cancer Immunome Atlas (https://tcia.at) and integrated with SMYD2 expression data by matching sample identifiers. Samples were stratified into

SMYD2-high and SMYD2-low groups using the median SMYD2 expression value as cutoff. IPS values corresponding to different therapeutic scenarios ($CTLA4^{-}/PD1^{-}$, $CTLA4^{-}/PD1^{+}$, $CTLA4^{+}/PD1^{-}$, $CTLA4^{+}/PD1^{+}$) were analyzed. All analyses were performed in R, and graphs were generated using the *ggplot2* R package or GraphPad Prism.

In vivo HCC murine models

Male C3H/HeJ and female C57BL/6J mice (6–8 weeks old) were bred and housed in the Animal Resources Facilities at Austral University. All procedures were approved by the university's Animal Studies Committee and conducted in compliance with institutional and national regulations for animal research and welfare. Mice were monitored every other day for body weight and general health throughout all *in vivo* experiments. Tumor dissection, size measurement and weighing were performed in a blinded manner to minimize potential bias. For monotherapy experiments, animals received AZ-505 (40 mg/kg, MedKoo Biosciences), LLY-507 (10 mg/kg, MedKoo Biosciences) or vehicle (12.5% Cremophor EL, 12.5% DMSO in aqueous solution) via intraperitoneal (i.p.) injection three times per week for two weeks. In combination studies, anti-PD-1 antibody (Bio X Cell) was co-administered at 100 μg/mouse (100 μL of a 1 mg/mL solution in PBS) via i.p. injection, together with the first and fourth AZ-505 doses.

In the Hepa129 model, liver fibrosis was induced in C3H/HeJ mice by i.p. injection of thioacetamide (TAA, 200 mg/kg, Sigma-Aldrich) three times per week for four weeks. On day 30, 1.25×10^{5} Hepa129 cells were injected subcapsularly into the left liver lobe via laparotomy. Treatment started six days later and followed the schedule described above. In the PM299L model, orthotopic tumors were generated by subcapsular injection of 5×10^{4} PM299L cells into the left liver lobe and treatment began four days post-implantation. For both models, mice were euthanized after the sixth dose of AZ-505, and

liver samples were collected. Tumor burden was determined using caliper measurements with the formula: $(\pi/6) \times \text{length} \times (\text{width})^2$ for Hepa129 tumors and by tumor weight for PM299L tumors. Ascites grade was also assessed in the Hepa129 model.

Synergy between AZ-505 and anti–PD-1 was evaluated using the fractional tumor volume (FTV) method. For each treatment group, tumor volume was normalized to the mean tumor volume of the control group to obtain the FTV. Expected FTV values for the combination were calculated as the product of the FTVs observed with each monotherapy (FTV_expected = FTV_A × FTV_B). A synergy index was then derived as the ratio of the expected to the observed FTV (Index = FTV_expected / FTV_observed). An index > 1 indicates synergistic interaction, ≈ 1 indicates additivity, and < 1 indicates antagonism.[1]

Quantitative real-time PCR

Complementary DNA (cDNA) synthesis was performed from 500 ng of RNA using 200 U of SuperScript II Reverse Transcriptase (Invitrogen, USA) and 500 ng of Oligo(dT) primers. Transcript levels of IFNγ, IL-1β, CCL5, TGFβ, CD11c, CD8, CD4, iNOS, ARG1, CCND1, AXIN2, SP5, c-MYC and LEF1 were quantified by real-time PCR using SYBR® Green (Invitrogen), using primer sequences listed in Supplemental Table S1. PCR cycling conditions consisted of an initial denaturation at 95 °C for 10 min, followed by 45 cycles of 95 °C for 30 s, 60 °C for 30 s, and 72 °C for 1 min. Melt curve analysis was performed by increasing the temperature from 60 °C to 95 °C at a rate of 2 °C/min, recording fluorescence every 15 s. GAPDH and ACTB were used as internal controls for normalization. Relative gene expression was calculated using the ΔΔCt method and expressed as fold change relative to the control condition. All samples were run in technical triplicates, and a non-template control was included in every assay.

Macrophage stimulation with tumor-derived conditioned media

PM299L and NM53 G03 cells were seeded in 100-mm culture dishes at a density of 2 × 10^6 cells per dish in DMEM supplemented with 10% FBS. After 24 hours, cells were treated overnight with AZ-505 (5 µM) or vehicle (DMSO) in complete medium. The following day, cultures were washed twice with saline solution and incubated with serum-free DMEM for 24 hours. Supernatants were collected and centrifuged at 500 × g for 10 minutes to obtain the conditioned media (CM), which were used immediately or stored at -80 °C. In parallel, J774 macrophages were seeded in 6-well plates at a density of 4 × 10^5 cells per well in RPMI-1640 medium supplemented with 10% FBS. After 24 hours, macrophages were incubated for 20 hours with a 1:4 dilution of CM obtained from PM299L or NM53 cells (± AZ-505). Where indicated, co-treatments with the Wnt pathway modulators CHIR99021 (10 µM, GSK3 inhibitor) or DKK1 (200 ng/mL, Wnt inhibitor) were included during CM exposure. Control macrophages were incubated with fresh medium under the same conditions.

Isolation of splenocytes and tumor-infiltrating cells

Spleens and tumors were collected from PM299L tumor–bearing mice treated with vehicle, AZ-505, anti-PD-1, or the combination therapy. For splenocytes isolation, spleens were gently dissociated in a petri dish using the plunger of a disposable syringe, filtered through a cell strainer and centrifuged (1500 rpm, 5 min). Tumor lysates were prepared by enzymatic digestion with 0.05% collagenase I (Sigma–Aldrich, USA) at 37 °C for 45 min, mechanical dissociation with a syringe plunger through a 70 µm mesh, and centrifugation (2500 rpm, 5 min, 4 °C). For both preparations, red blood cells were lysed with ACK buffer for 5 min at room temperature, and cells were washed (1500 rpm, 5 min), resuspended in physiological saline, and kept on ice until use.

Statistical analyses

Statistical analyses were conducted using R software version 4.4.2 and GraphPad Prism version 8.0 (GraphPad Software, Carlsbad, USA). Data are presented as mean ± standard error of the mean (SEM). Depending on the distribution of the data, comparisons between groups were performed using either parametric (t-test, ANOVA) or non-parametric tests (Mann–Whitney U test, Kruskal–Wallis test). Normality was assessed using the D'Agostino–Pearson omnibus test. A p-value and FDR below 0.05 were considered statistically significant. Graphs were generated using either Prism or R (*ggplot2* package), and final figures were assembled in Adobe Illustrator (Adobe Systems, USA).

Supplemental figures

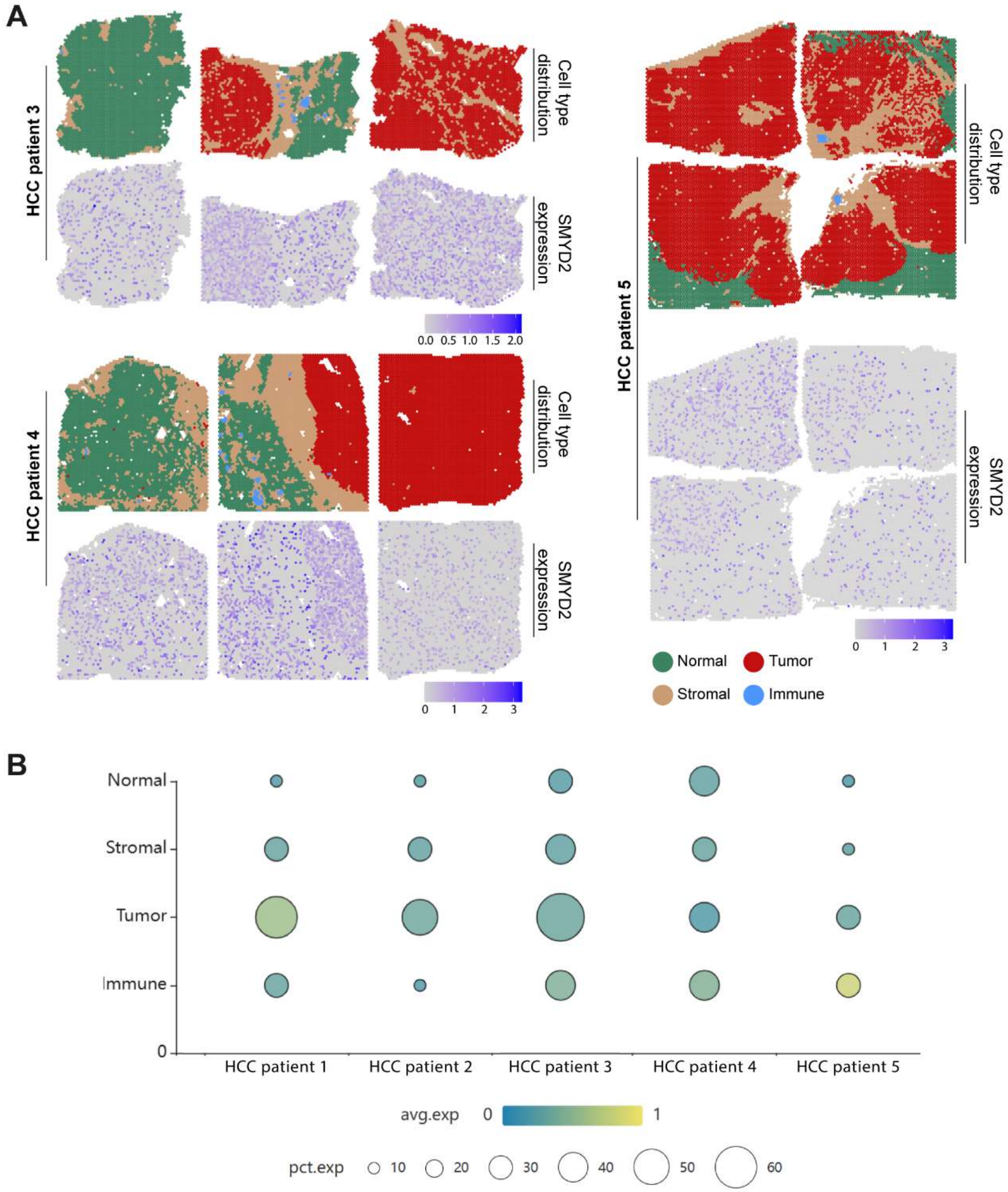

Figure S1. (A) Public spatial transcriptomic data showing SMYD2 expression in liver samples from three HCC patients. Top panels show cell type annotation; bottom panels display SMYD2 expression intensity across spatial coordinates. (B) SMYD2 expression in annotated cell populations. Patient identifiers from the HCCDB v2 database are as

follows: HCC Patient 1 corresponds to HCC-2, Patient 2 to HCC-4, Patient 3 to HCC-1, Patient 4 to HCC-3, and Patient 5 to HCC-4.

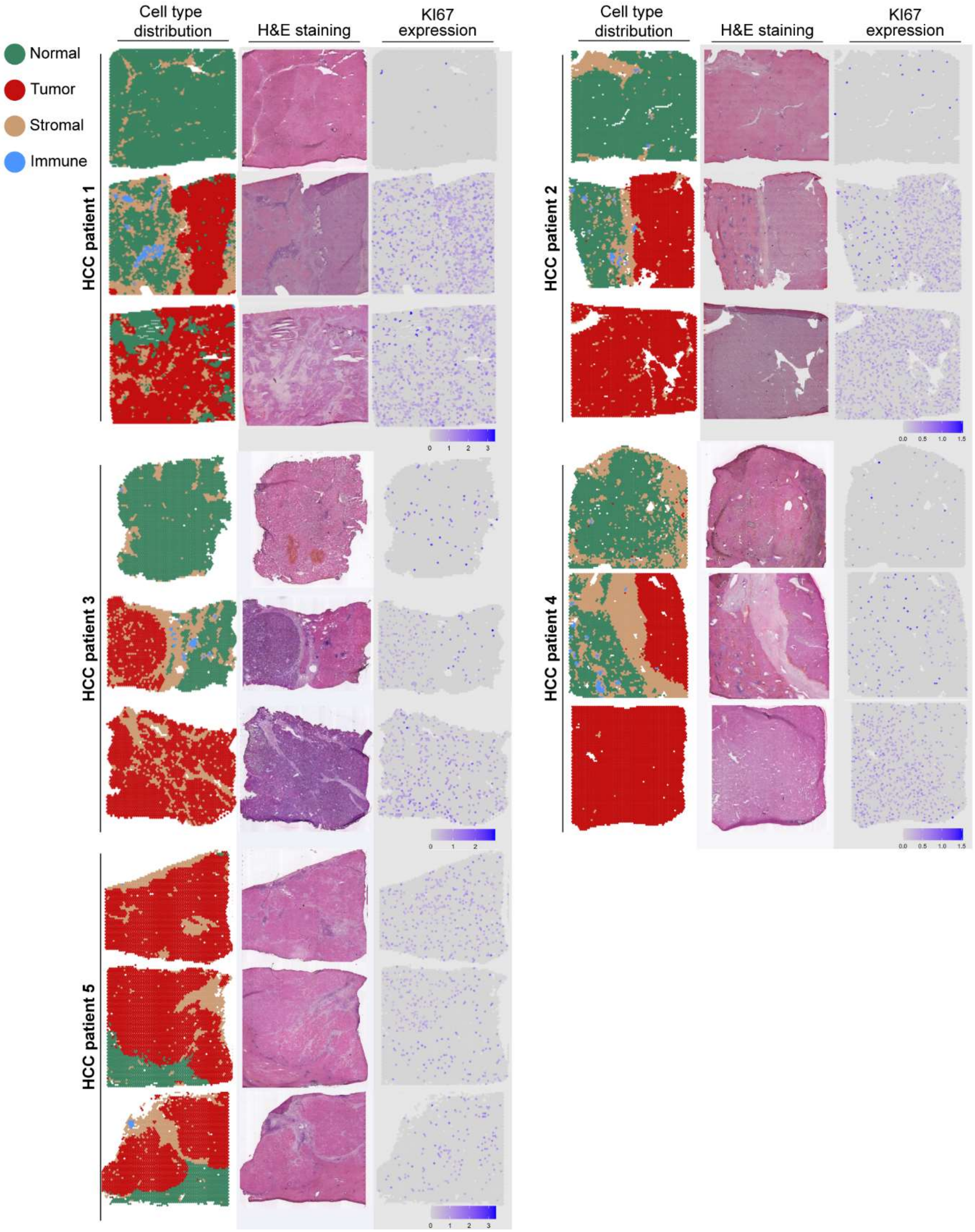

Figure S2. Public spatial transcriptomic data showing cell type annotation (left panels), corresponding H&E staining (middle panels), and Ki67 expression (right panels) in liver samples from HCC patients. Patient identifiers correspond to those described in Figure S1. The cell type distribution panels of patients 1 and 2 were previously presented in Figure 1B, whereas those for patients 3, 4, and 5 were previously presented in Figure S1.

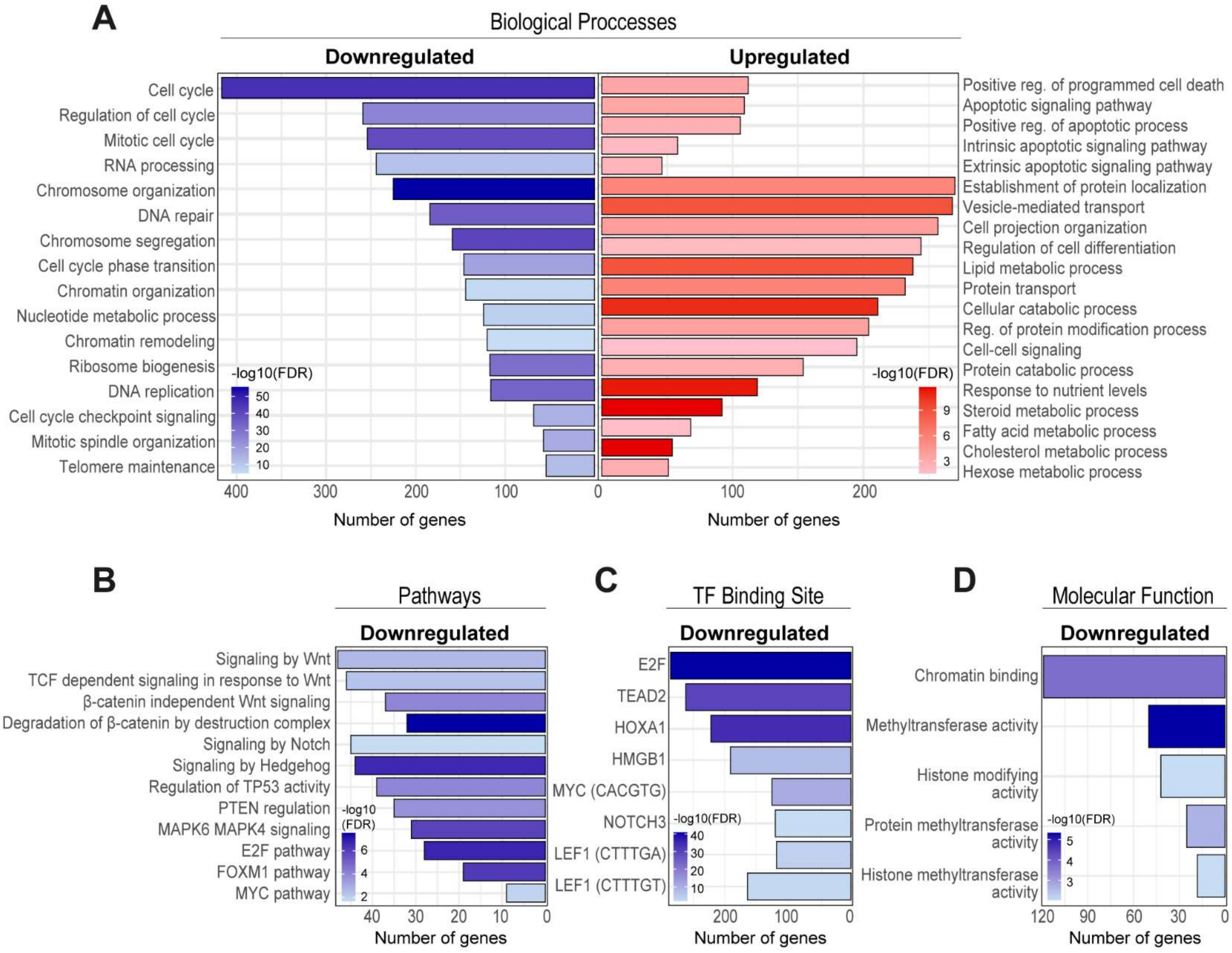

Figure S3. Functional enrichment analysis of DEGs in HuH7 cells treated with LLY-507. GO and pathways analysis of (A) biological processes, (B) pathways, (C) transcription factor binding sites, and (D) molecular function of upregulated or downregulated genes identified by RNA-seq.

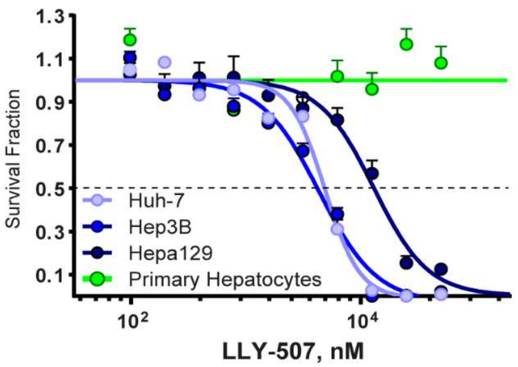

Figure S4. Dose-response curves of human (Huh7, Hep3B) and murine (Hepa129) HCC cell lines, and primary hepatocytes treated with LLY-507 for 72 h. Viability was assessed by MTT. Curves were fitted by non-linear regression and are representative of 2 independent experiments (4 replicates each, shown as mean ± SEM).

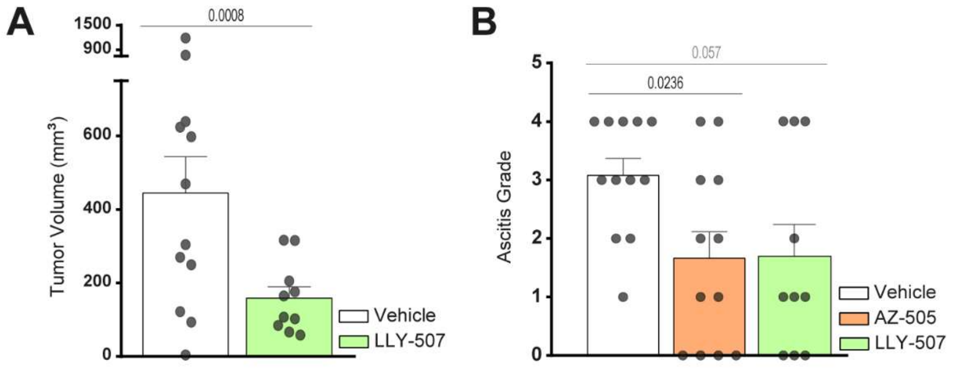

Figure S5. AZ-505 and LLY-507 reduce tumor burden and ascites in an orthotopic Hepa129 HCC model. (A) Tumor volume and (B) ascites grade (assessed visually) at endpoint in C3H/HeN mice bearing Hepa129 tumors treated with vehicle (n = 12), AZ-505 (n = 12) or LLY-507 (n = 10), as described in Fig. 3c. Bars represent mean ± SEM. Statistical analyses were performed using Mann-Whitney (vs. vehicle). Exact p-values are shown in the graphs.

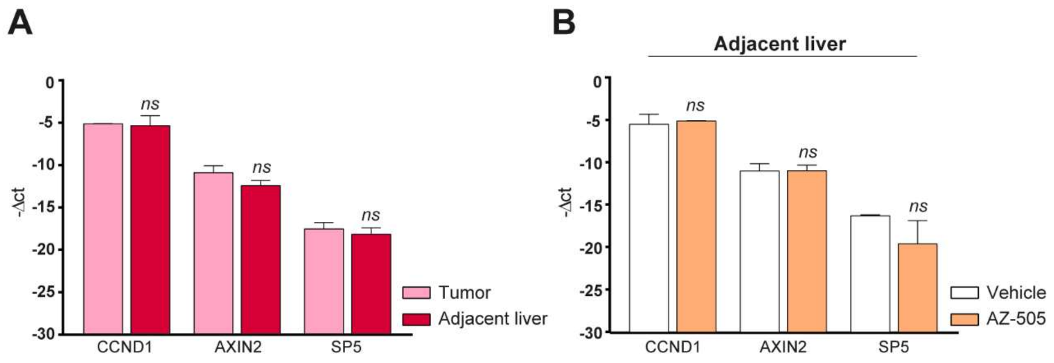

Figure S6. Wnt/β-catenin target gene expression in Hepa129 tumors and adjacent liver tissue. ΔCt values for Ccnd1, Axin2, and Sp5 measured by qPCR in (A) tumors and paired adjacent liver tissue, and (B) adjacent liver tissue from Hepa129-bearing mice treated with vehicle or AZ-505. Bars represent mean ± SEM. ns, not significant (t-test).

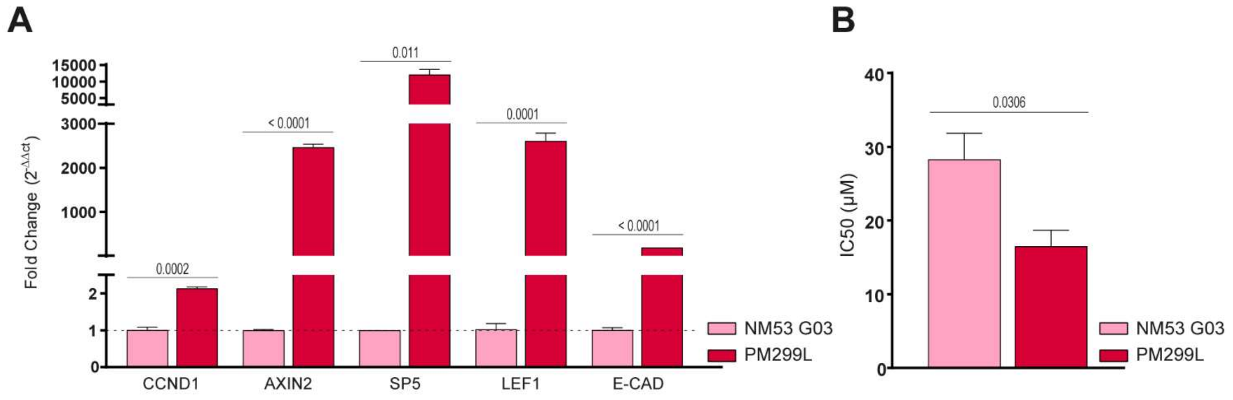

Figure S7. Basal expression of Wnt/β-catenin target genes and sensitivity to AZ-505 in murine HCC cell lines. (A) mRNA expression of Ccnd1, Axin2, Sp5, Lef1, and E-cadherin was assessed by qRT-PCR in PM299L and NM53 G03 cells under basal conditions. Data are shown as fold change (2^−ΔΔCt) relative to NM53 G03. Bars represent mean ± SEM of technical triplicates. (B) IC50 values were determined by MTT assay after 72 h of AZ-505 treatment in PM299L and NM53 G03 cells. Bars represent mean ± SEM of three (NM53 G03) or four (PM299L) independent experiments, each performed in technical quadruplicates. Statistical analyses were performed using t-test (vs. NM53 G03). Exact p-values are shown in the graphs.

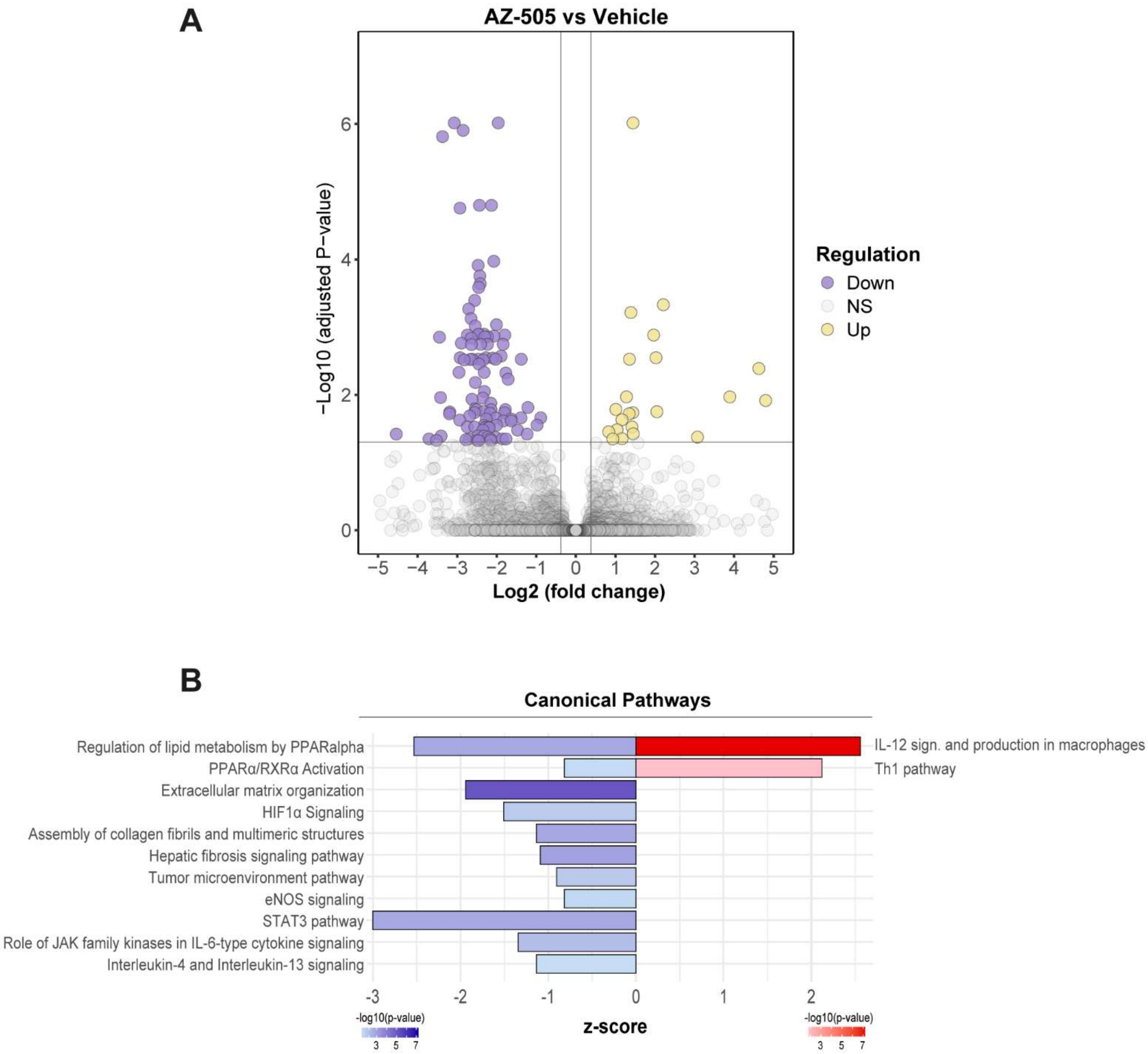

Figure S8. RNA-seq analysis of AZ-505-treated PM299L tumors. (A) Volcano plot showing differentially expressed genes (log2(FC)>|0.378|, FDR<0.05) in PM299L tumors from mice treated with vehicle or AZ-505. Yellow and purple dots indicate significantly up- or downregulated genes, respectively; gray dots indicate non-significant genes. (B) IPA-based pathway analysis of DEGs in PM299L tumors treated with AZ-505 versus vehicle.

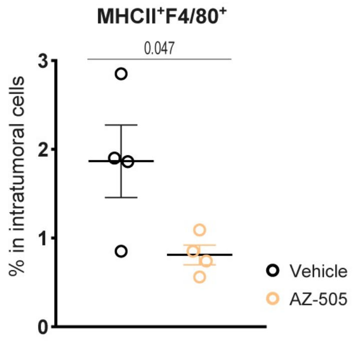

Figure S9. Frequency of MHCII^{+}F4/80^{+} cells within the intratumoral compartment. Tumors from PM299L-bearing mice treated with vehicle or AZ-505 were dissociated and analyzed by flow cytometry. Bars represent mean ± SEM. Statistical analyses were performed using unpaired t-test (vs. vehicle). Exact p-values are shown in the graphs.

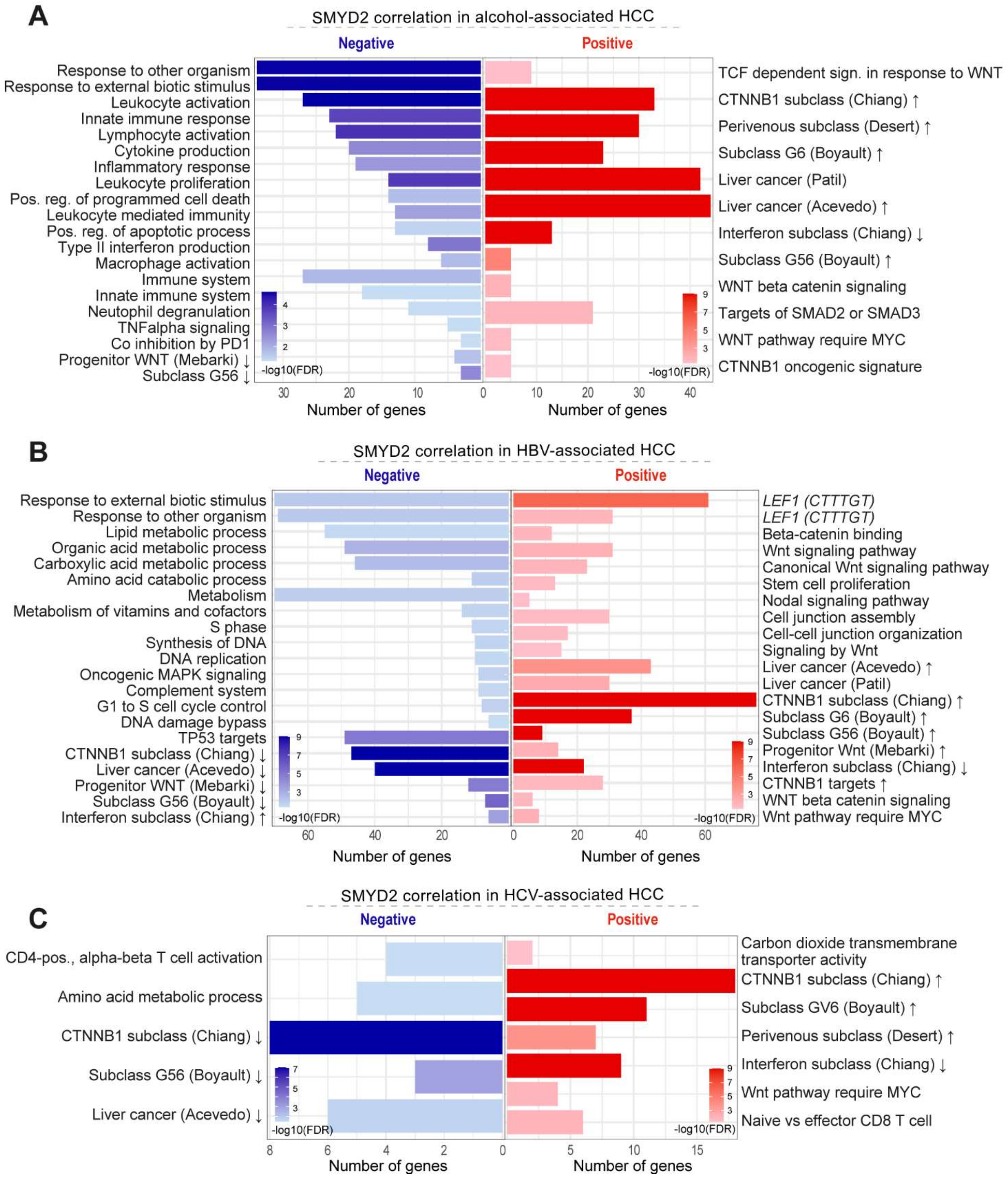

Figure S10. SMYD2-associated transcriptional programs across HCC etiologies. (A–C) Functional enrichment analysis of genes positively (red) and negatively (blue) correlated with SMYD2 expression in TCGA-LIHC patients stratified by etiology: alcohol-associated (A), HBV-associated (B), and HCV-associated HCC (C). Bar plots

show the number of genes contributing to each enriched term, with color intensity reflecting statistical significance (−log10 adjusted p-value) and arrows denoting regulation trends. Terms in *italics* indicate “Transcription Factor Binding Site” annotations; others belong to “Coexpression”, “Biological process” and “Pathway” categories.

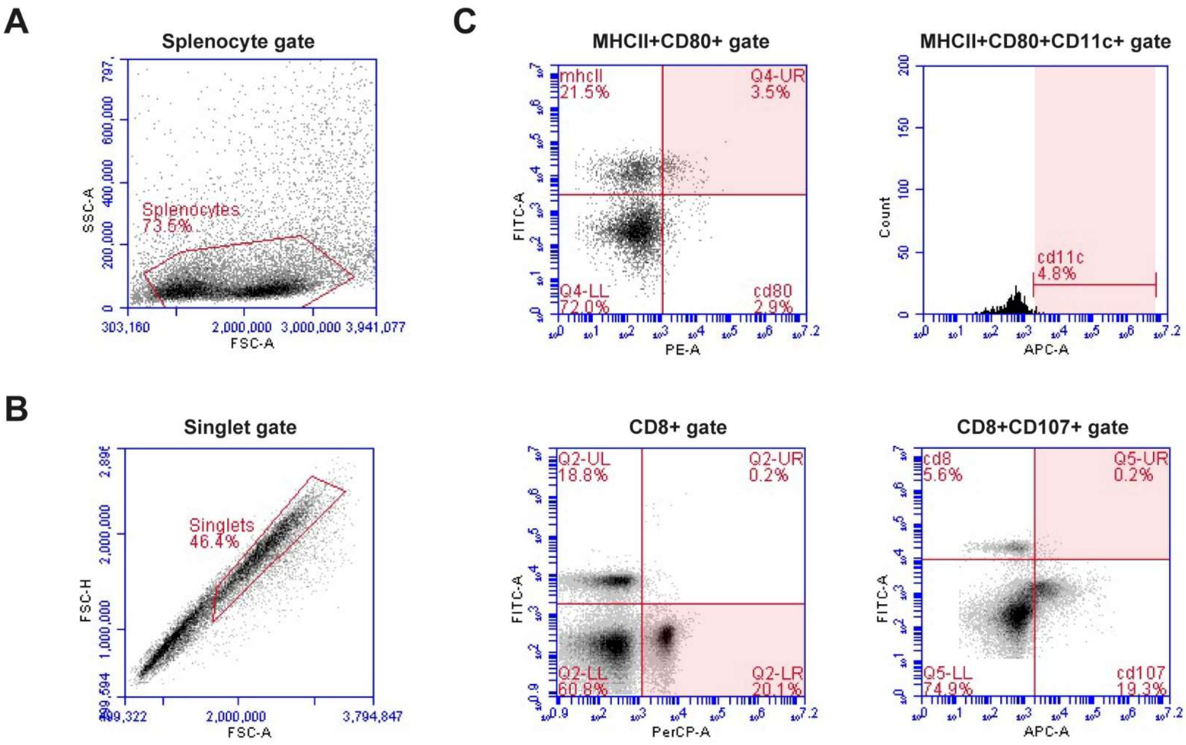

Figure S11. Gating strategy for flow cytometry analysis of splenocytes. (A) Initial gating based on FSC-A vs. SSC-A to select total splenocyte population. (B) Singlets were gated using FSC-A vs. FSC-H, applying a conservative gate to enrich for viable, single cells. (C) Representative dot plots used to define splenocyte subpopulations. Upper left: cells co-expressing MHCII and CD80 were identified. Upper right: within this population, $CD11c^{+}$ cells were gated. Lower left: $CD8^{+}$ T cells were identified; CD4 was also included in the staining panel but not analyzed. Lower right: $CD8^{+}CD107a^{+}$ cells were quantified from a separate set of splenocytes stimulated ex vivo. Red rectangles indicate the gated populations used for quantification.

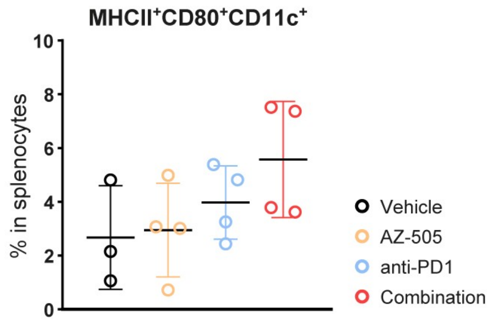

Figure S12. Frequency of splenic MHCII⁺CD80⁺CD11b⁺ antigen-presenting cells. Tumors from PM299L-bearing mice treated with vehicle, AZ-505, anti-PD-1, or the combination were dissociated and analyzed by flow cytometry.

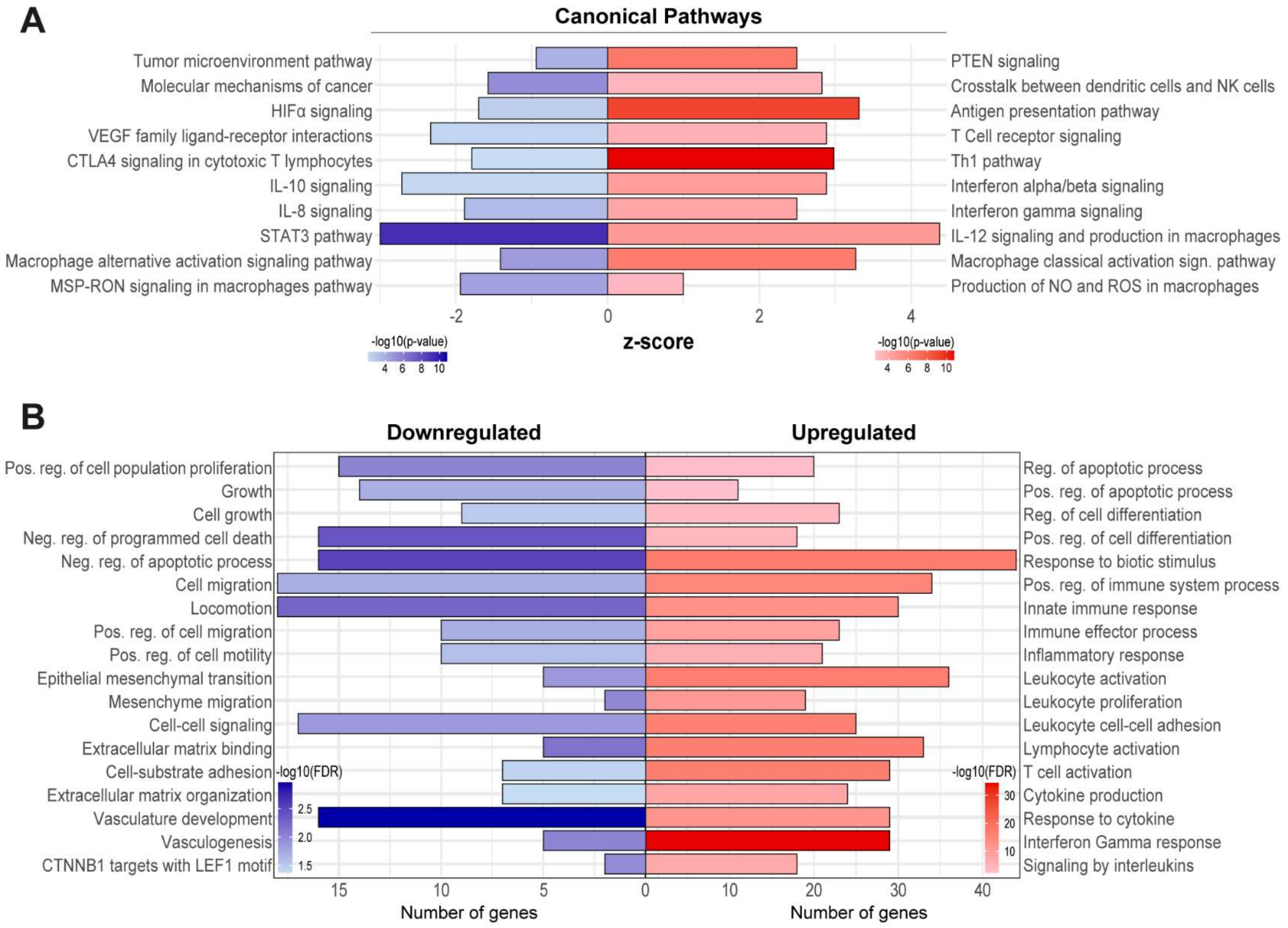

Figure S13. (A) IPA-based pathway analysis of DEGs in PM299L tumors treated with combination therapy versus vehicle. (B) Gene Ontology (GO) enrichment analysis (biological process and pathway categories) of upregulated and downregulated genes.

Supplemental tables

Table S1: oligonucleotides sequences for quantitative real-time PCR assays.

Gene	Forward	Reverse
GAPDH	5'GGGGCTGCCCAGAACATCAT	5'GCCTGCTTCACCACCTTCTTG
ACTB	5'CACTGTCGAGTCGCGTCC	5'CCTTCTGACCCATTCCCACC
SMYD2	5'TGGAATCCTTCGGAGACTGTG	5'CATTACAGTTCCACCTGGGCG
IFNγ	5'GGAACTGGCAAAAGGATGGTGA	5'TGACGCTTATGTTGTTGCTGA
IL-1β	5'TGACAGTGATGAGAATGACCTGTTC	5'TTGGAAGCAGCCCTTCATCT
CCL5	5' CCTGCTGCTTTGCCTACCTCTC	5' ACACACTTGGCGGTTCCTTCGA
TGFβ	5'ACCAACTATTGCTTCAGCTC	5'TGTTGGTTGTAGAGGGCAAG
CD11c	5'ACGTCAGTACAAGGAGATGTTGGA	5'ATCCTATTGCAGAATGCTTCTTTACC
CD8	5'CCGTTGACCCGCTTTCTGT	5'CGGCGTCCATTTTCTTTGGAA
CD4	5'AGGTGATGGGACCTACCTCTC	5'GGGGCCACCACTTGAACTAC
iNOS	5'AAGATGGCCTGGAGGAATGC	5'TGCTGTGCTACAGTTCCGAG
ARG1	5'CAGAAGAATGGAAGAGTCAG	5'CAGATATGCAGGGAGTCACC
CCND1	5'TCAAGTGTGACCCGGACTGC	5'CCTTGGGGTCGACGTTCTG
AXIN2	5'AATAAGCAGCCGTTCGCGATG	5'AGCCTCCTCTCTTTTACAGCAA
SP5	5'TCCAGACCAACAAACACACCA	5'AGTTTGCCGCTACCCAATCA
c-MYC	5'ATGCCCCTCAACGTGAACTTC	5'GTCGCAGATGAAATAGGGCTG
LEF1	5'GCCACCGATGAGATGATCCC	5'TTGATGTCGGCTAAGTCGCC

References

1. Cantero MJ, Bueloni B, Gonzalez Llamazares L, Fiore E, Lameroli L, Atorrasagasti C, et al. Modified mesenchymal stromal cells by in vitro transcribed mRNA: a therapeutic strategy for hepatocellular carcinoma. Stem Cell Res Ther. 2024 Jul 11;15(1):208.
